# Unravelling highly oxidized nickel centers in the anodic black film formed during the Simons process by *in situ* X-ray absorption near edge structure spectroscopy†

**DOI:** 10.1039/d3sc06081k

**Published:** 2024-02-14

**Authors:** Gene Senges, Ana Guilherme Buzanich, Tilen Lindič, Tyler A. Gully, Marlon Winter, Martin Radtke, Bettina Röder, Simon Steinhauer, Beate Paulus, Franziska Emmerling, Sebastian Riedel

**Affiliations:** a Freie Universität Berlin, Fachbereich Biologie, Chemie, Pharmazie, Institut für Chemie und Biochemie – Anorganische Chemie Fabeckstrasse 34/36 14195 Berlin Germany s.riedel@fu-berlin.de; b BAM Federal Institute for Materials Research and Testing Richard-Willstätter-Str. 11 12489 Berlin Germany; c Freie Universität Berlin, Fachbereich Biologie, Chemie, Pharmazie, Institut für Chemie und Biochemie – Theoretische Chemie Arnimallee 22 14195 Berlin Germany

## Abstract

The Simons process is an electrochemical fluorination method to prepare organofluorine compounds. Despite the wide application, the underlying mechanism is still unclear. We report the investigation of the black film formed on the surface of the anodes in aHF by an *in situ* Ni K-edge X-ray absorption near edge structure (XANES) investigation. An electrochemical cell for *in situ* X-ray absorption spectroscopy (XAS) is presented.

## Abstract 2

The electrofluorination after Simons has been used for the last century to produce everyday life materials. An *in situ* XANES investigation of the controversially debated black film apparent in the Simons process revealed high-valent nickel centers.

## Graphical abstract

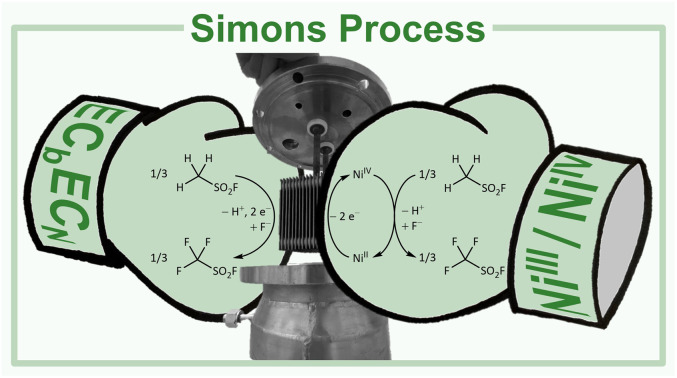

The Simons process is an electrochemical method with an exceptionally high functional group tolerance for the generation of industrially important compounds such as triflic acid or perfluorobutane sulfonic acid (PFBS) from their non-fluorinated derivatives.^1–6^ PFBS, the follow-up and potentially less toxic homologue^6,7^ of perfluorooctane sulfonic acid (PFOS; annual production of up to 4500 t),^8^ was similarly used to protect fabrics from water, soil and stain.^1,3,6^ A row of functionalized perfluorocarbons including perfluorooctyl bromide (PFOB) are of medical interest as oxygen carriers or “blood substitutes”.^1,9^ Within the broad range of applications for triflic acid,^10,11^ it depicts the starting material for the production of lithium bis(trifluoromethanesulfonyl)imide (LiTFSI), which is commonly used in electrolytes of Li-ion batteries.^11,12^ Given the value of the fluorinated compounds produced,^1,3,5,6,13^ the Simons process is performed with abundant resources, using anhydrous hydrogen fluoride (aHF) as fluoride source, electricity, nickel electrodes, and simple organic starting materials.^1,3,14–16^ However, in this process also partially fluorinated species are obtained, while simultaneously concurring fragmentation of the products leading to, *e.g.*, CF_4_, is observed.^2,17^

The reason for the formation of these species and the mechanism of the Simons process are still under debate.^1–3,17,23,31^ In principle, two mechanisms have been proposed: (I) either the fluorination proceeds *via* the electrochemical oxidation of the substrate and consecutive reaction with HF following the EC_b_EC_N_ mechanism (explanation in ref. 32; Fig. 1, left).^3,18–23^ (II) Alternatively, high potential oxidizers have been proposed (NiF_3_, NiF_4_, NiF_2_⋅F_2_, or fluorine radicals) to be generated electrochemically, which are reacting consecutively with the organic substrates to the corresponding fluorinated products (Fig. 1, right).^1,2,4,17,24–31^ Both mechanisms have been investigated in the last decades, while indications were found for both of them.^1,3,23,31,33,34^ The presence of cationic intermediates as proposed for the EC_b_EC_N_ mechanism is supported by the observation of products that are typical of cationic isomerization processes.^2,3,22,23,34^ However, as even ammonium salts such as [NMe_4_]^+^ are fluorinated a reaction *via* the EC_b_EC_N_ mechanism of those species would require a dicationic intermediate, which is unlikely to exist.^17^ The mechanism of electrochemical fluorination (ECF) mediated by nickel-based high potential oxidizers is supported by the fact that nickel electrodes previously anodized under the conditions of the Simons process are able to convert organic molecules to the corresponding fluorinated species even when no electric potential is applied (open circuit conditions).^1,2,26,31^ This indicates that in the electrochemical process oxidizing nickel agents are involved, mediating the fluorination.^1,2,26,31^ Further evidence for this hypothesis is provided by Bartlett and coworkers demonstrating that both under Simons conditions and by using the highly pure R-NiF_3_ acetonitrile is fluorinated yielding similar products.^1,2,35,36^ Ignat'ev and coworkers observed a black film on the anodes that decomposed rapidly to a yellow-green substance. They proposed that this black film consists of black NiF_3_ which was reduced to yellow-green NiF_2_.^1,2,36–38^ When Ni surfaces exposed to gaseous or liquid HF or after Simons electrochemical fluorination were examined by *ex situ* X-ray photoelectron spectroscopy (XPS), only NiF_2_ was detected.^39,40^ The behavior of nickel anodes was studied in a broad range of hydrogen fluoride based media, but the black film was not investigated *in situ* and the presence of higher oxidized nickel species during the Simons process remains still unclear.^1,29,39,41^ Nevertheless several proposed mechanisms use the interaction of an NiF_3_ surface with the substrate to rationalize the observed products.^1^ The elucidation of the anodic surface may help to further optimize the Simons process in the future.

**Fig. 1:**
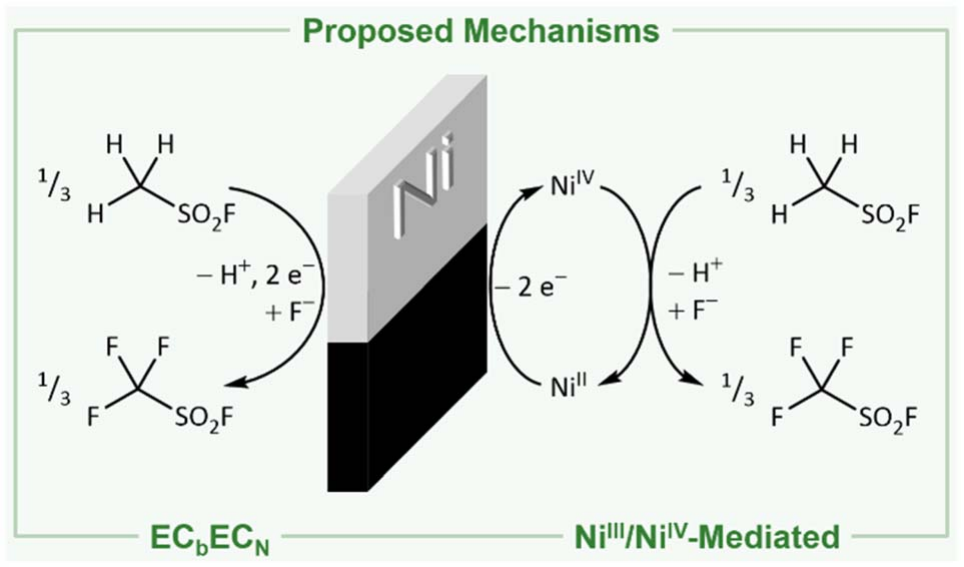
Net reactions for the production of trifluoromethyl sulfonic acid fluoride following the direct electrochemical oxidation *via* the EC_b_EC_N_ mechanism (left)^3,10,18–23^ and the fluorination mediated by electrochemically generated Ni^III^/Ni^IV^ (right).^1,2,4,10,17,24–31^

Aided by solid state quantum-chemical calculations and reference spectra obtained from the powders of K_2_NiF_6_, NiF_3_ and – for the first time – NiF_4_, we report an unprecedented *in situ* X-ray absorption near edge structure (XANES) investigation to reveal the existence of high-valent Ni centers on the surface of the anode in the Simons process.

The Simons process was investigated under conditions closely matching the industrial ones (aHF, Ni electrodes, cell potential of +4.5 to +7.0 V, current density of 0.5 to 3.0 A dm^−2^).^1,3,15,16^ We speculated that the black film could be degraded by organic substrates, which could complicate its spectroscopic identification. However, it has been demonstrated by Ignat'ev and coworkers that the organic substrates are fluorinated even when the electrochemical cell is disconnected from the power supply. This means that an oxidizer was produced on the anode before the organic substrate is added, which renders the fluorination independent of the anodic process.^26^ This justifies the investigation of the anodic process in the absence of an organic substrate.

In a preliminary experiment, we studied the formation of the black film in dependence on the applied cell voltage. As already shown by our group, there is only one oxidation feature of the nickel anode at +3.57 V *vs.* Pt-QRE (platinum quasi-reference electrode), which was attributed to the formation of a higher oxidized nickel species, while at higher potentials no other discriminable oxidation peaks were found in the cyclic voltammogram.^42^ In several independent experiments we could show that this oxidation process leads to the formation of the black film, as already emphasized by Ignat'ev and coworkers.^1,2^ After preconditioning a nickel anode at +6.0 V cell voltage, we decreased the potential in 0.1 V steps and observed the stepwise degradation of the black film by chronoamperometric measurements, implying that its layer thickness depends on the voltage applied (Fig. S24 and S25†). As our aim is the *in situ* characterization of the black film by XANES, it is important to have a layer thickness of at least 50 μm to achieve a sufficient signal-to-noise ratio. Therefore, we polarized a nickel anode at +6.0 V cell voltage for 120 min, removed it from the electrochemical cell and analyzed it by scanning electron microscopy (Fig. S28†). On the anode's surface, particles with an average size of 50 to 100 μm were found, indicating this procedure sufficient to form a black film thick enough for XANES spectroscopy (see the ESI†).

Based on these preliminary analyses, we designed and constructed a setup enabling *in situ* XANES measurements of the anode's surface in aHF. This setup had to meet certain requirements: (I) resistance against gaseous and liquid aHF, (II) a cell temperature maintained at 0 °C to limit the HF vapor pressure, (III) a window as close as possible to the anode's surface to reduce X-ray absorption by the electrolyte. Therefore, we designed an electrochemical cell, consisting of a polychlorotrifluoroethylene (PCTFE) body with closed circuit cooling, a fluorinated ethylene propylene copolymer (FEP) window (50 μm-foil), planar nickel electrodes embodied in polytetrafluoroethylene (PTFE), and fluorine kautschuk material (FKM) O-ring sealed perfluoroalkoxy alkane (PFA) tubings (Fig. 2, top, and S44 to S51†). The cell was characterized by cyclic voltammetry and showed only one discriminable oxidation feature at +5.25 V cell voltage (Fig. S41†). This deviation from the literature accounts for the overpotential caused by the electrode arrangement required for the planned *in situ* XANES measurements.^42^ In a first experiment, the cell was filled with pre-cooled aHF and the surface of the anode was examined by *in situ* XANES without applying an external potential, revealing that the surface consists of Ni^0^ and small quantities of NiF_2_ (Fig. 3 and 4), readily formed by the contact of the nickel electrode with aHF, as previously observed by Scherson and co-workers.^40^ Then, we applied a cell potential stepwise rising from +5.5 V to +8.9 V and observed the formation of the black film (Fig. 2, bottom, Fig. 3). To further increase the film thickness, an additional conditioning phase at a cell voltage of +8.7 V was maintained for 127 min (Fig. S5 and S7†). After the XANES characterization of the black film its decomposition was further monitored under open-circuit conditions (Fig. S8 and S9†).

**Fig. 2:**
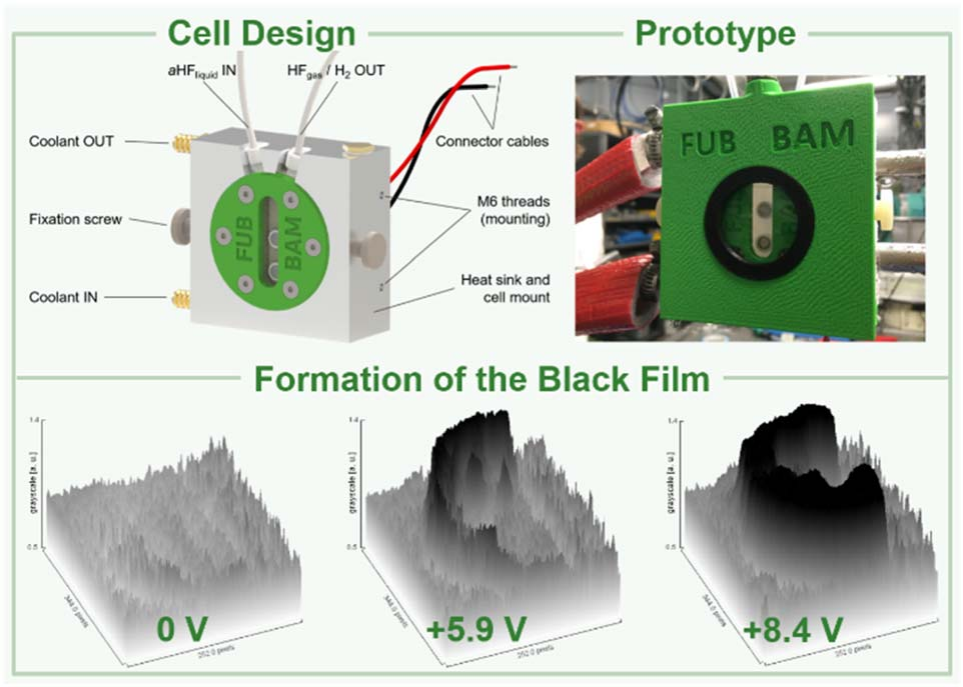
Design and prototype of an electrochemical *in situ* XAS cell (top) and the propagation of the black film at different cell potentials (greyscale *versus* electrode area derived from photographs, bottom).

**Fig. 3:**
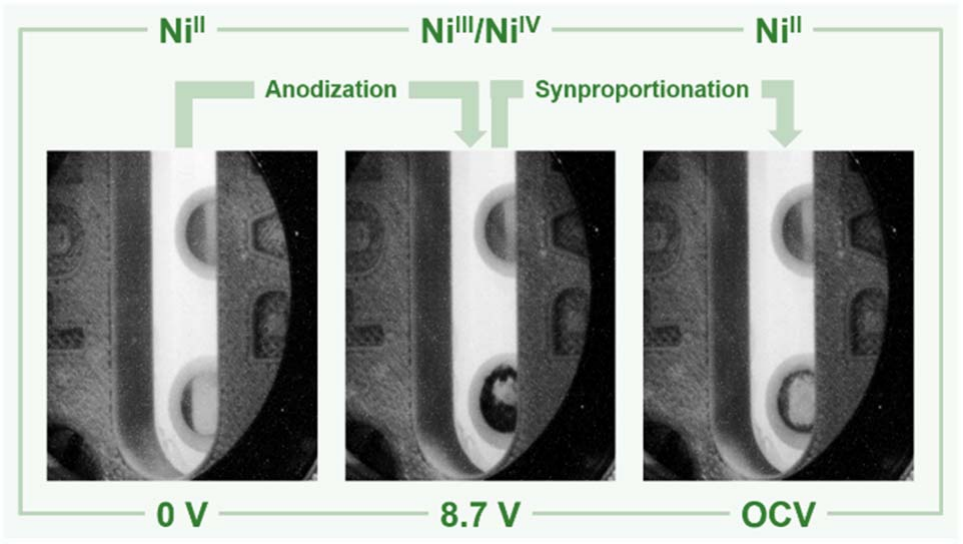
Nickel anode (bottom) and cathode (top) in liquid anhydrous hydrogen fluoride before applying a potential (left), at +8.7 V (center), and after decomposition of the black film (OCV = open circuit voltage, right). The color of the anode's surface changes from silvery to black upon applying a potential of +8.7 V and decolorizes during the OCV measurement, while the cathode remains visibly unchanged.

Even though we polished the electrodes carefully to a mirror finish, the center of the anode was slightly elevated, resulting in a shorter distance to the FEP foil, and consequently, at this spot no black film could be observed. Therefore, we focused the beam on the ‘blackest’ region of the anode (Fig. S10†).

In the XANES spectrum of the black film we observe a shoulder at 8337.0 eV, and the broader shape right after the edge suggests the contribution of features at 8352.5 and 8354.0 eV. The absorption edge at 8337.0 eV can be attributed to Ni^0^ of the underlying bulk material of the electrode and the peak at 8352.5 eV is assigned to Ni^II^ centers, as confirmed by comparison to a sample of NiF_2_. More importantly, the absorption maximum at 8354.0 eV is characteristic for highly oxidized nickel centers,^38,43^ as the XANES spectra of the reference substances K_2_NiF_6_, NiF_3_ and NiF_4_ feature coinciding white lines at 8354.6 eV (Fig. S35†). The spectra of NiF_3_ and K_2_NiF_6_ measured in transmission resemble the features observed in the spectra in fluorescence (see Fig. S38†). Notably, the XANES spectrum of NiF_4_, which is the first spectral data obtained from this thermally unstable compound,^37^ features another peak at 8369.1 eV, while for NiF_3_ and the black film no maximum was found in this area. These reference spectra were recorded with a low temperature XAS cell (Fig. S52 and S53†).

In order to support these experimental findings, we optimized the structures of binary nickel fluorides and K_2_NiF_6_ (Table 1) within the periodic density functional theory (DFT) framework (see the ESI†). Two structures were evaluated for the stoichiometric composition NiF_3_, one with equal (NiF_3_, space group *R*3̄*c*) and one with different Ni–F distances (Ni^II^[Ni^IV^F_6_], space group *R*3̄). With respect to the different Ni–F distances, a strong distortion of the first F-coordination shell was found for Ni^II^[Ni^IV^F_6_], in contrast to NiF_3_. The necessary use of different *U* values for Ni^II^, Ni^III^ and Ni^IV^ centers renders a direct comparison of the energies difficult. However, the energies of NiF_3_ and Ni^II^[Ni^IV^F_6_] are comparably large, thus the different synthetic methods for NiF_3_ – chemically or electrochemically – might force the formation of the monovalent or the mixed-valent species. The comparison of the averaged Ni–F distances of 1.88 and 1.83 Å for Ni^III^ and Ni^IV^, respectively, with 2.02 Å in NiF_2_, supports our assignment of the features in the XANES spectra. In agreement with these theoretical results and the literature,^38,44–46^ we determined Ni–F distances of 1.75 (Ni^IV^) and 1.88 Å (Ni^II^) for NiF_3_ and 1.80 Å for K_2_NiF_6_ by their EXAFS (Fig. S40 and Table S2†). These computational results indicate that the black film contains a high-valent nickel fluoride with nickel centers in an oxidation state larger than +II. To further elucidate these observations, we stopped applying a cell potential, thereby initiating the decomposition of the black film while measuring XANES spectra for additional 120 min to monitor the chemical processes behind (Fig. 4). Within 120 min the intensity of the peak at 8352.0 eV was found to significantly increase, while also a peak at 8370.0 eV appears (Fig. 3). As both peaks are indicative for NiF_2_, it can be assumed that the decomposition of the black film leads to an increase of the NiF_2_ content. Additionally, we observed a substantial decrease of the absorption at 8337.0 eV (Ni^0^) comparing the black film and the decomposed film. Thus, simultaneously to the formation of NiF_2_ the amount of Ni^0^ decreases, revealing a synproportionation of the high-valent nickel fluoride and the electrode material Ni^0^ to the decomposition product NiF_2_.^2^ This development of the anodic black film is confirmed by linear combination fitting analysis of the spectra (Fig. S18 to S23†). Upon its decomposition at open circuit conditions, the black film decolorized (Fig. 4, right) and the *in situ* open circuit voltage (OCV) scan showed a residual cell potential of about +2.0 V decreasing over 90 min to approximately 0 V (Fig. S6†). This can be explained by the synproportionation of highly oxidized nickel centers and Ni^0^ to Ni^II^ leading to an anode with an NiF_2_ surface layer of increased thickness. As the cathode is covered with NiF_2_ due to passivation by aHF as well,^40^ after the synproportionation two similar electrodes are obtained, having an electrochemical potential of approximately 0 V.

**Table 1:** Calculated space groups (SG), magnetic phases (MP, AF = antiferromagnetic, D = diamagnetic), formal oxidation states of the nickel centers (OS), Ni–F distances (*d* given in Å, av = average), and energies normalized to NiF_2_ (*E*_norm_, given in eV, calculated with an averaged *U* value of 6.6 eV)

	NiF_2_^a^	Ni_2_F_5_^a^		NiF_3_^a^	Ni^II^[Ni^IV^F_6_]		NiF_4_	K_2_NiF_6_
SG	*P*4_2_/*mnm*	*C*2/*c*		*R*3̄*c*	*R*3̄		*P*2_1_/*c*	*Fm*3̄*m*
MP	AF	AF		AF	AF		AF	D
OS (Ni)	+2	+2	+3	+3	+2	+4	+4	+4
*d*(Ni–F)	2.024	1.930	1.847	1.883	2.009	1.829	1.860	1.777
	2.024	2.154	1.834	1.883	1.987	1.828	1.860	1.777
	2.024	2.171	1.941	1.883	1.950	1.860	1.933	1.777
	2.024	2.154	1.834	1.883	1.986	1.817	1.836	1.777
	2.016	2.171	1.941	1.883	1.970	1.817	1.933	1.777
	2.016	1.930	1.847	1.883	2.003	1.824	1.836	1.777
*d*_av_(Ni–F)	2.021	2.085	1.874	1.883	1.984	1.829	1.876	1.777
*E*_norm_	0	+0.3297		+0.2302	+0.5674		+1.0091	—

a The data were taken from the ref. 42, 43 and 44 respectively.

**Fig. 4:**
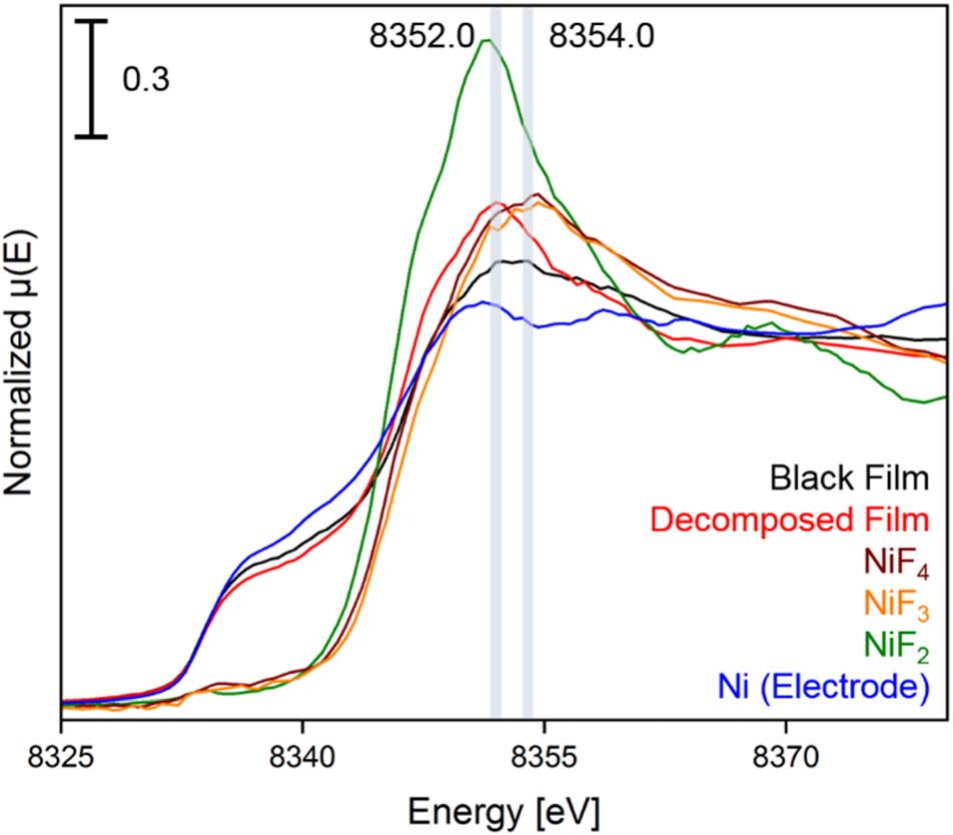
Ni K-edge spectra of the black film (black), the decomposed film (red), and the reference compounds NiF_4_ (brown), NiF_3_ (orange), NiF_2_ (green), and Ni (bulk anode exposed to HF, blue) with an acquisition step size of 0.5 eV. The positions of the maxima of the white lines are highlighted for the black and the decomposed film. The first derivatives of the spectra and time dependent spectra of the black film are shown in Fig. S11 to S17.†

## Conclusions

In conclusion, we investigated the black film formed on the surface of the nickel anodes in the Simons process by means of XAS. As it has been speculated for decades that the active species of the Simons process has to be found in the black film, we analyzed the anode's surface employing a tailor-made XAS electrochemical cell. This cell consists of perfluorinated materials and electrodes closely adjustable to the FEP window, allowing for the use of aHF as an electrolyte and enabling *in situ* Ni–K edge XANES measurements of the anode's surface with a good signal-to-noise ratio. This cell design is not limited to the use of HF and nickel electrodes, but might be adapted for the investigation of numerous other electrochemical processes by XAS or even other methods. Only Ni^0^ and Ni^II^ centers were apparent on the anode prior to the application of a cell potential, while at high potentials XANES spectroscopy revealed the existence of high-valent Ni centers in the black film. Based on our results, the mechanistic understanding and thus the efficiency of the Simons process can be improved.

## Data availability

Most of the informations is already in the ESI.† However more data is available by the corresponding author.

## Author contributions

G. S. and S. R. formulated and coordinated the project. G. S. conceptualized the experiments including designs of the XAS cells, with contributions from A. G. B., M. R., B. R., S. S., F. E. The experiments have been performed by G. S. with contributions from M. W., T. A. G. T. L. has performed and analyzed the DFT calculations. B. P. has critically evaluated the computational data and discussed with all coauthors. G. S. visualized experimental data and the graphical representation of the XAS cells with contributions from A. G. B. and B. R. G. S. wrote the first draft of the manuscript. All authors discussed the result of the different disciplines and proofread the publication.

## Conflicts of interest

There are no conflicts to declare.

## Supplementary Material

SC-015-D3SC06081K-s001
