# Supplementary material for: Unravelling highly oxidized nickel centers in the anodic black film formed during the Simons process by in situ X-ray absorption near edge structure spectroscopy
Source: Chem Sci. 2024 Feb 14;15(12):4504–9. doi: 10.1039/d3sc06081k (PMC10952067; doi:10.1039/d3sc06081k)
Supplement: SC-015-D3SC06081K-s001 [file SC-015-D3SC06081K-s001.pdf]

Electronic Supplementary Material (ESI) for Chemical Science.
This journal is © The Royal Society of Chemistry 2024

Supporting Information

Unravelling Highly Oxidized Nickel Centers in the Anodic Black Film Formed During the Simons Process by In-Situ X-Ray Absorption Near Edge Structure Spectroscopy

Gene Senges, Ana Guilherme Buzanich, Tilen Lindič, Tyler A. Gully, Marlon Winter, Martin Radtke, Bettina Röder, Simon Steinhauer, Beate Paulus, Franziska Emmerling, and Sebastian Riedel*

Table of Contents

1 Experimental details 4

1.1 Electrochemical measurements 4

1.1.1 Electrochemical measurements (ex-situ) / preliminary results 4

1.1.2 Electrochemical measurements (in-situ) 4

1.2 XANES measurements 5

1.3 EXAFS measurements 6

1.4 Syntheses of the reference compounds 6

1.5 PXRD measurements 6

1.6 Surface morphology (SEM) 6

1.7 Data analysis and manipulation 6

2 In-situ characterization of the black film 8

2.1 In-situ generation and decomposition of the black film 8

2.2 Additional in-situ XANES data 15

2.3 Linear combination fitting of the in-situ XANES data 23

3 Ex-situ characterization and standards 27

3.1 Generation and decomposition of a black film (ex-situ) 27

3.1.1 PXRD data of a decomposed film 29

3.1.2 SEM images of a decomposed film 30

3.1.3 Ex-situ XANES data of a decomposed film 31

3.2 Higher nickel fluorides NiF_4 and NiF_3 35

3.2.1 Sample productions 35

3.2.2 XANES data 35

3.2.3 EXAFS data 39

3.3 Electrochemical characteristics of the in-situ XAS cell 42

4 XAS cells – construction and functional principles 45

4.1 In-situ XAS cell 45

4.2 Low temperature XAS cell for reference measurements of NiF_4 and NiF_3 53

5 Computational details 55

5.1 Benchmarking the Davidson *U* value for Ni^{4+} 55

5.2 Calculating the structure of mixed-valent NiF_3 ($Ni^{II}[Ni^{IV}F_6]$) 55

5.3 Calculation of the solid state structure of NiF_4 56

5.4 Calculation of the NiF_2-normalized energies 56

6 References 56

1 Experimental details

1.1 Electrochemical measurements

All electrochemical experiments – open circuit voltage scan (OCV), chronoamperometry (CA) and cyclic voltammetry (CV) – were performed using a *Bio Logic Science Instruments Pvt. Ltd.* SAS model SP-300 potentiostat and the software *EC-Lab®* using two-electrode setups consisting of nickel working and counter electrodes in open (preliminary setup, Section **3**) or closed cell designs (in-situ XAS cell, Section **2** and Section **4**).

1.1.1 Electrochemical measurements (ex-situ) / preliminary results

The nickel plate (99.72 %, *institute's stock*) electrodes for the generation of scanning electron microscopy (SEM) and ex-situ X-ray absorption near-edge structure (XANES) samples were welded to nickel wires (99.98 %, *ChemPur*), which were isolated in PFA tubing to only allow the contact of liquid anhydrous hydrogen fluoride (*a*HF) and to prevent the contact of the electrodes with gaseous HF (Fig. S22). The plate electrodes were immersed in nitric acid (65 %) for 1 min, immediately rinsed with deionized water and dried in air prior to use.

Safety warning: Anhydrous HF can cause deep tissue damage and exhibits systemic toxicity. Safe handling of HF demands personal protective clothing and dedicated first aid equipment and training. Do not attempt the use of HF without training and specialized equipment.

Anhydrous hydrogen fluoride was obtained from *GHC Gerling Holz & Co. Handels GmbH* and used as received for preliminary electrochemical experiments (Section **3.1**). The required amount of *a*HF (ca. 10 ml) was condensed in a PFA (perfluoroalkoxy alkanes) reservoir with a PTFE (polytetrafluoroethylene) seal plug with drilled holes for HF inlet and outlet at −196 °C by usage of a stainless-steel high vacuum line. The *a*HF was then transferred via PFA tubing into the electrochemical cell (PFA tube (Ø = 2.54 cm, flame-sealed bottom) equipped with a PTFE seal plug with drilled holes for the HF inlet, the gas outlet (H_2, HF_{gas}) and the electrodes by applying a slight overpressure of argon at −80 °C. During the experiments, the temperature of the electrolyte *a*HF was maintained at 0 °C by an ice/water bath.

1.1.2 Electrochemical measurements (in-situ)

The planar electrodes used for the in-situ studies were made from nickel rods (Ø = 5 mm, 99.99 %, *ChemPur*) embodied in PTFE (Ø = 10 mm) and were polished to a mirror finish with diamond (1 µm) and alumina (0.05 µm) polishing pastes by using corresponding polishing pads from an electrode polishing kit supplied by *Zimmer & Peacock*, sonicated in an isopropanol bath for 3 min after each polishing step and dried in air.

The *a*HF was dried over K_2NiF_6 (99 %, *ABCR*) prior to the experiments employing the in-situ XAS cell and the production of the NiF_4/NiF_3 samples. The in-situ XAS cell was filled with *a*HF in analogy to the ex-situ experiments (Section **1.1.1**), while the PFA transfer-tube was flame sealed after the filling was completed. The in-situ XAS cell was cooled by a closed-cycle cryostat (coolant: ethanol) with the temperature set at −5 °C. Since the aluminum cooling body of the in-situ XAS setup was always colder than the electrochemical cell, the water only condensed on the aluminum without impairing the XANES measurements even after more than ten hours.

The electrochemical experiments during the in-situ characterization of the species on the anode were performed in a non-stop program with the consecutive steps:

1) OCV scan for 10 min (Fig. S3)
2) CA at +5.5 to +8.9 V, increased in 0.1 V steps per 10 min (Fig. S4)
3) CA at +8.7 V for 127 min (Fig. S5)
4) OCV for 120 min (Fig. S6)

1.2 XANES measurements

XANES measurements were carried out at the Ni K-edge (8333 eV) at the BAM*line*,[1] located at BESSY II (Berlin, Germany). The incident energy was tuned by a double crystal monochromator in a Si(111) arrangement ($\Delta E/E = 2 \cdot 10^{-4}$), in 10 eV steps until 20 eV before the edge, followed by 0.5 eV steps until 30 eV above the edge and 1 eV steps until 200 eV above the edge. The beam size was 2 mm (H) x 1 mm (V). Owed to the irregular sample surface and X-ray thick sample environment, the measurements were performed in fluorescence mode, where 4-element silicon drift detectors are collecting the signal emitted backscattered and focusing on a point at about 10 mm in front of the detector’s head.

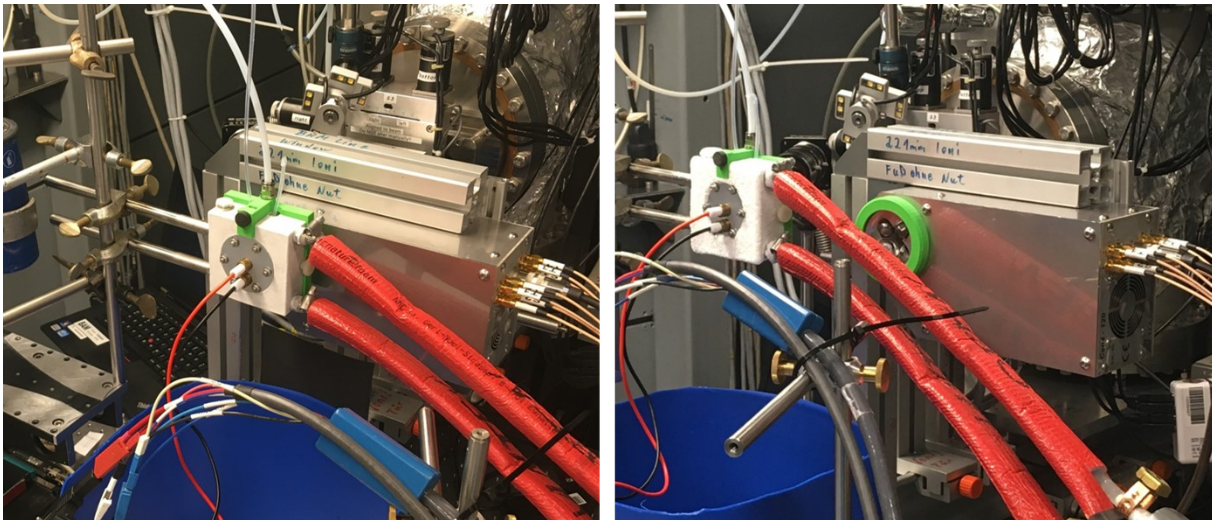

Figure S1: Setup of in-situ XAS cell in XANES measurement (left) and photography (right) positions. The detector was moved aside after each XANES measurement and a photo was taken from the sample, respectively.

XANES spectra were recorded at the specific temperatures where the phases are reportedly stable (Table S1).[2]

Table S1: Sample temperatures during XANES measurements.

Sample	ϑ_{sample}
NiF_4	< −60 °C
In-situ electrode	−5 °C
NiF_3	0 °C
K_2NiF_6	r. t.
NiF_2	r. t.
Ex-situ electrode	r. t.
Ni foil	r. t.

1.3 XAFS measurements in transmission

XAFS measurements containing both XANES and EXAFS were performed in transmission of Ni foil, NiF_3 and K_2NiF_6. This allows us to corroborate the features of the XANES region obtained previously in fluorescence, as well as to elucidate the local coordination environment by looking at the EXAFS part of the spectrum. These measurements were performed at the same beamline as the ones performed in the in-situ measurements in fluorescence mode (cf. Section 1.2). Two ionization chambers were used to measure the I_0 signal (before the sample) and the I_1 signal (after the sample). For I_0 and I_1 a 5 cm and 15 cm long chamber was used, respectively. The chambers were filled with air. The measurement protocol was the following: 10 eV steps until 20 eV before the edge, followed by 0.5 eV steps until 17 eV above the edge and 2 eV steps until 200 eV above the edge. From then on equidistant k-steps were taken (every 0.06 Å) until 16 Å. The data evaluation and treatment were performed by using the ATHENA program from the DEMETER package.[3] The EXAFS signal was Fourier-transformed between 2–12 Å with a Hanning-type window, to obtain a radial distribution-like information (Fig. S40). Schematic drawings of the corresponding XAS cell that was used for the measurements of NiF_3 and K_2NiF_6 are depicted in Fig. S54 and Fig. S55. Further EXAFS data evaluation was performed using the ARTEMIS program from the DEMETER package. In this case, the experimental data were fitted to diffraction data of literature-known structures.[4,5] The results from this fit are shown in Table S2. In the case of NiF_3 the fit was performed between 1 and 3.7 Å, while for K_2NiF_6 between 1 and 3.6 Å.

1.4 Syntheses of the reference compounds

NiF_4 was synthesized by dispersing K_2NiF_6 (0.5 g, 2 mmol; 99 %, *ABCR*) in liquid *a*HF (3 ml) below −65 °C in a λ-shaped reactor made from FEP tubing (fluorinated ethylene-propylene copolymer; Ø =9 mm, 500 µm wall thickness). A light brown/tan colored precipitate formed instantly as BF_3 (*institute's stock*) was added stepwise at up to 1.5 atm, until the suspension (agitated with a Mini Vortex Mixer PV-1) did not absorb further BF_3 and the residual K_2NiF_6 was converted. The NiF_4 was purified in a procedure adapted from the literature[2] by decanting the supernatant solution of KBF_4 in *a*HF and distilling back the HF to wash the NiF_4 (repeated six times) while maintaining a temperature below −65 °C throughout the experiment. In order to save larger quantities of the NiF_4 sample, the *a*HF was removed under reduced pressure very slowly because of boiling retardation especially at temperatures as low as −65 °C and lower. The dry NiF_4 was transported under an argon atmosphere. NiF_4 was quantitatively decomposed to black NiF_3 at 0 °C within 30 min.

NiF_2 (1.5 mg, 0.016 mmol; 99 %, *ABCR*) was intensely ground with BN (52 mg, 2.1 mmol; 99.5 %, *Alfa Aesar*) and filled in a polycarbonate cell covered with 125 µm-Kapton® foil windows.

Ni foil (2 µm, 99.95 % *Goodfellow*) was used as received.

1.5 PXRD measurements

Powder X-ray diffractograms were measured on a *Bruker D8 Venture* diffractometer with a *PHOTON II CMOS* detector at 100(1) K using Mo-K_α irradiation at λ = 0.71073 nm via a 360° Phi scan with an exposure time of 60 s and a detector distance of 120 mm. The data were integrated with the *APEX3* program package (*Bruker*).

1.6 Surface morphology (SEM)

The surface morphology was investigated using a field emission scanning electron microscope (FE-SEM) model SU8030 by Hitachi (Chiyoda, Japan), at an accelerating voltage (V_{ac}) of 20 kV, a current of 20 µA, and a working distance of 8.5 in. The substrates were sputtered with a 5 nm conductive gold layer prior to the SEM measurements, using a CCU-010 sputter machine by Safematic GmbH (Bad Ragaz, Switzerland).

1.7 Data analysis and manipulation

XANES data were evaluated and treated by using the *Athena* program from the *Demeter* package.[3]This includes background removal, energy calibration with a Ni metal foil spectrum, and pre- and post-edge normalization. All phases in the XANES data were quantified by linear combination fitting (LCF) with reference substances also measured experimentally. The goodness of the fit was based on the χ^2 test and *R*-factor values.

All spectra acquired in fluorescence mode are in most cases affected by a so-called “self-absorption effect”. This is when fluorescent radiation created at deeper parts of the sample excites upper layers of the sample when trying to escape, which results in absorption of these photons, hence self-absorption. This hinders the features observed in the XANES curves. However, there are known algorithms that correct this effect, such as the one from Tröger et al.,[6] the so-called self-absorption correction (SA-Fluo). In this algorithm, only an estimate chemical formula of the sample and the geometry of the experiment, with incident and fluorescence angles, need to be introduced. In this work, all XANES spectra are corrected for self-absorption effects using this tool.

XANES and linear combination fitting data thereof, as well as electrochemical data and powder X-ray diffractograms were processed and manipulated with *Origin 2020*.[7] In addition to the visual inspection of the formation of the black film, the color development on the anode was analyzed by the program *ImageJ* (*Fiji*) based on color differences on the photos taken. The sample holder for the XANES measurement of a nickel electrode with a decomposed anodic film was drawn using the program *Fusion 360* (*AutoCAD*).

2 In-situ characterization of the black film

2.1 In-situ generation and decomposition of the black film

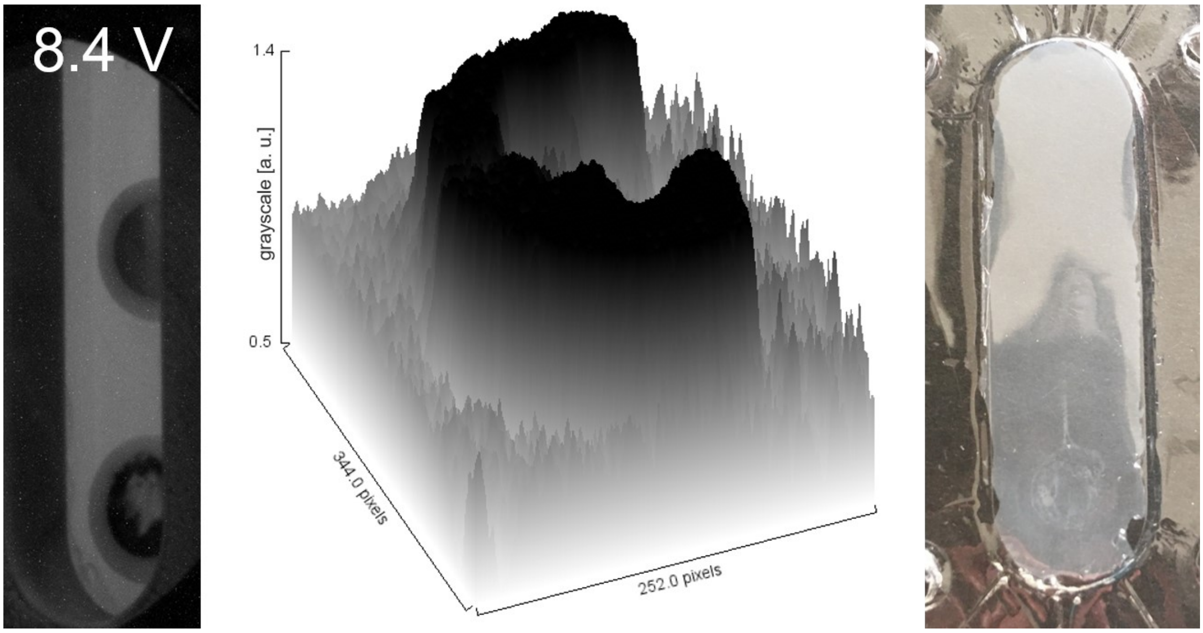

Figure S2: The black film formed on the anode at a cell potential of +8.4 V (left), the color development based on photographs taken during the formation of the black film between cell potentials of 0 V and +8.4 V (center), the FEP window after experiments (right). After the polishing process, the anode was slightly elevated in its center. In order to optimize the quantum yield, the closest possible distance was achieved between anode and FEP window, allowing only a thin layer of *a*HF in-between. Therefore, the center of the electrode was located closer to the window than to the rim. Any higher nickel fluoride formed on the anode's surface in areas very close to the window almost immediately reacted with the cell's window. Because of that, the FEP window was visibly attacked in these areas (in addition to becoming opaque upon the exposition to *a*HF), which is consistent with the non-uniform black surface on the anode. This demonstrates the high oxidation potential of the species apparent on the surface with respect to partial strong degradation of the fully fluorinated FEP material.

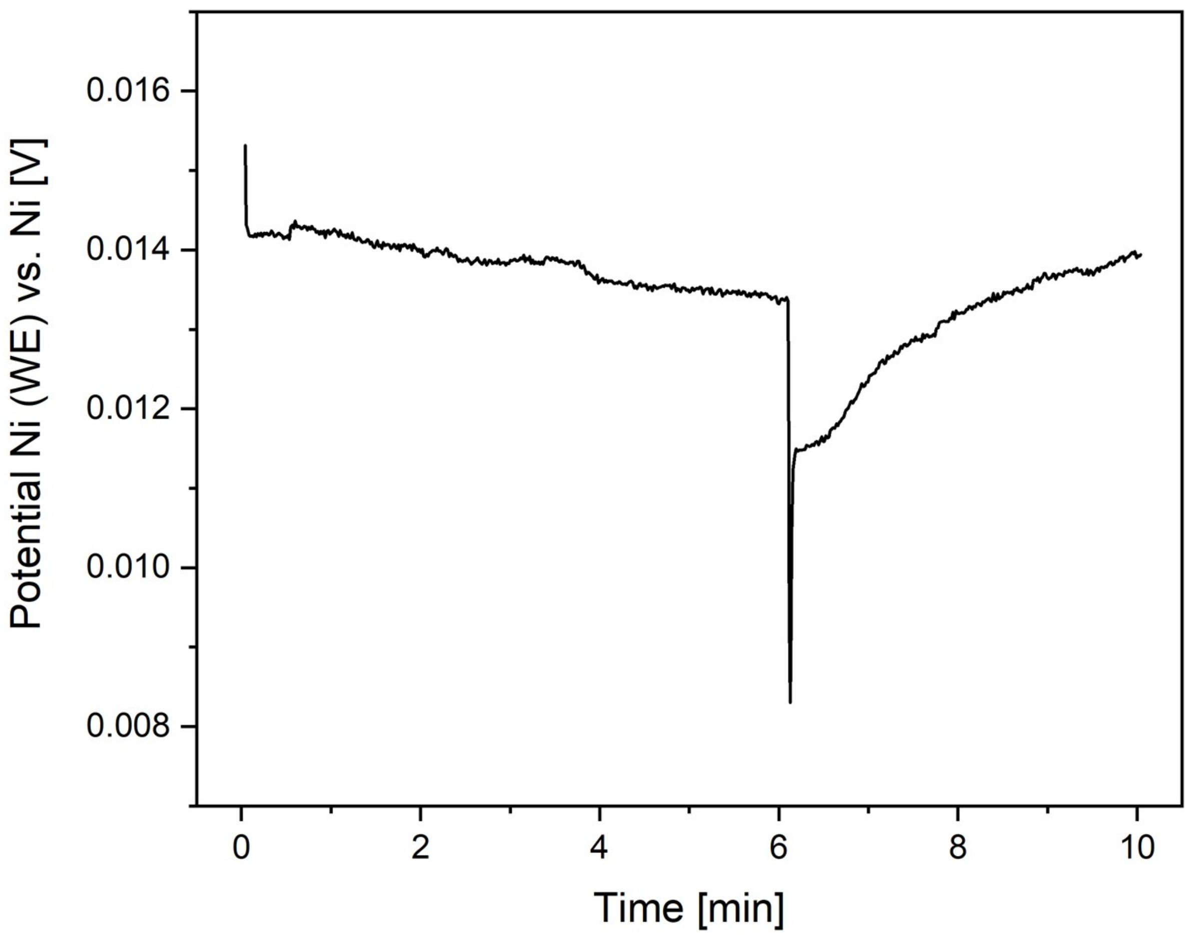

Figure S3: OCV scan in advance of the generation of the black film. The spike in potential is associated with vibrations induced by moving the detector.

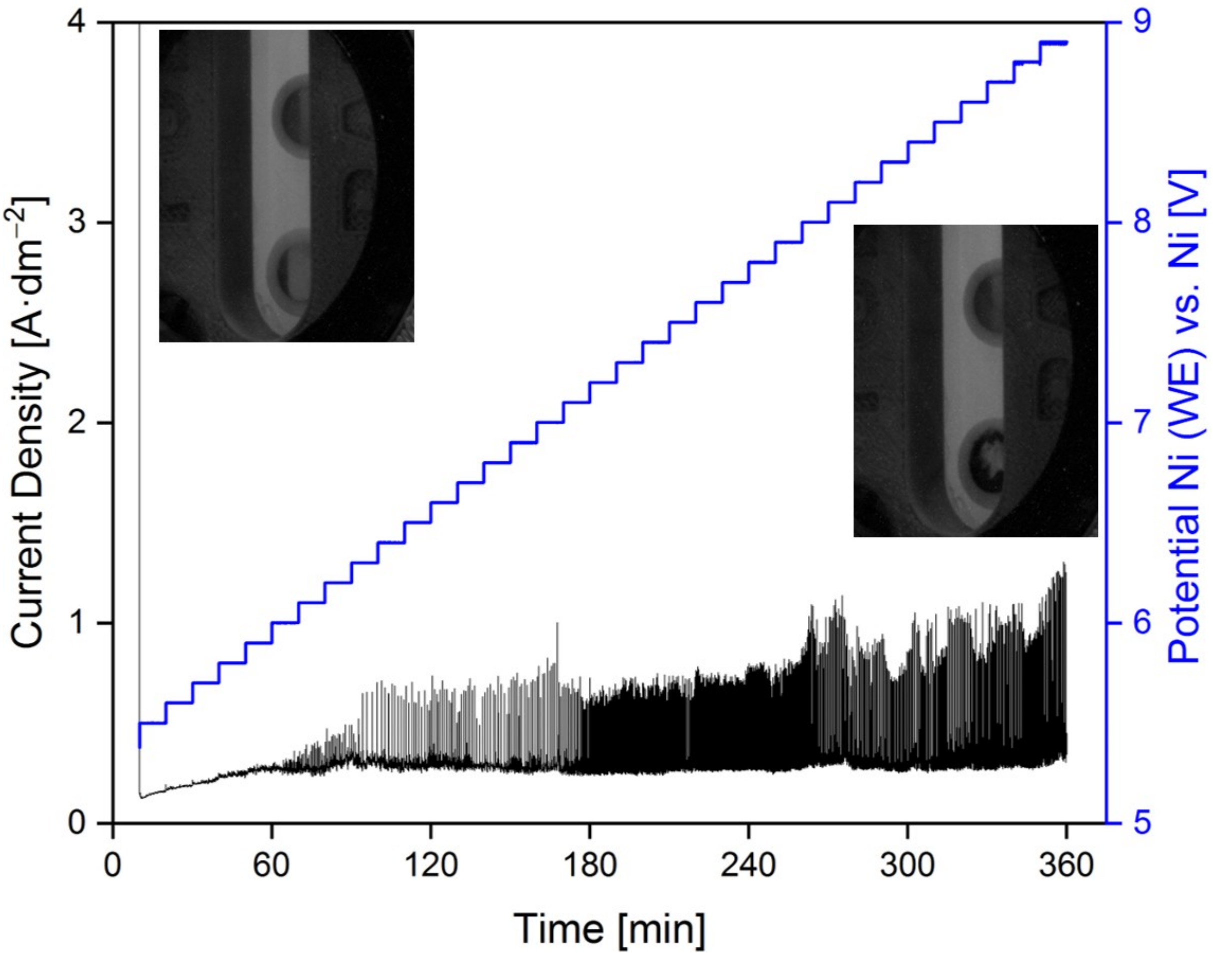

Figure S4: Chronoamperogram (black, left axis) and potential-time curve (blue, right axis) in the cell potential range of +5.5 to +8.9 V of the conditioning of the anode for the in-situ XANES studies. The highly frequent peaks in current density probably stem from the re-oxidation of the black film as a consequence of its reaction with the FEP window material (short anode-window distance, Fig. S3). Referring to the spikes at potentials of +8.8 and +8.9 V, the potential was not stable at cell potentials higher than +8.7 V (blue). The photographs depict the states of the electrodes (top: cathode, bottom: anode) at the start (10 min, left) and at the end (360 min, right).

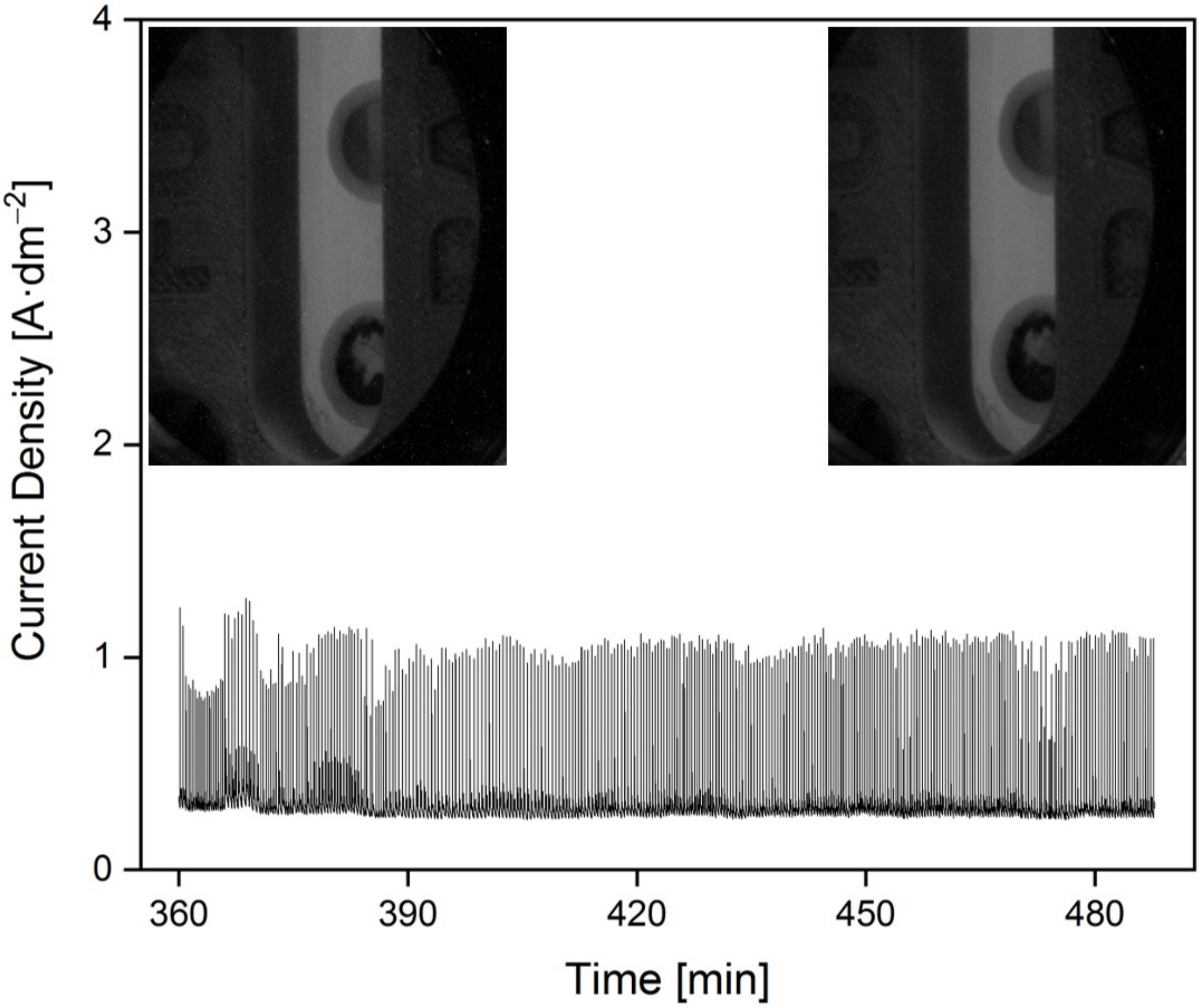

Figure S5: Chronoamperogram at a cell potential of +8.7 V during the prolonged conditioning period of the anode for the in-situ XANES studies. The time scale refers to the absolute run time of the experiment, including the conditioning phase with potential steps. The photographs depict the states of the electrodes (top: cathode, bottom: anode) at the start (360 min, left) and the end state (487 min, right).

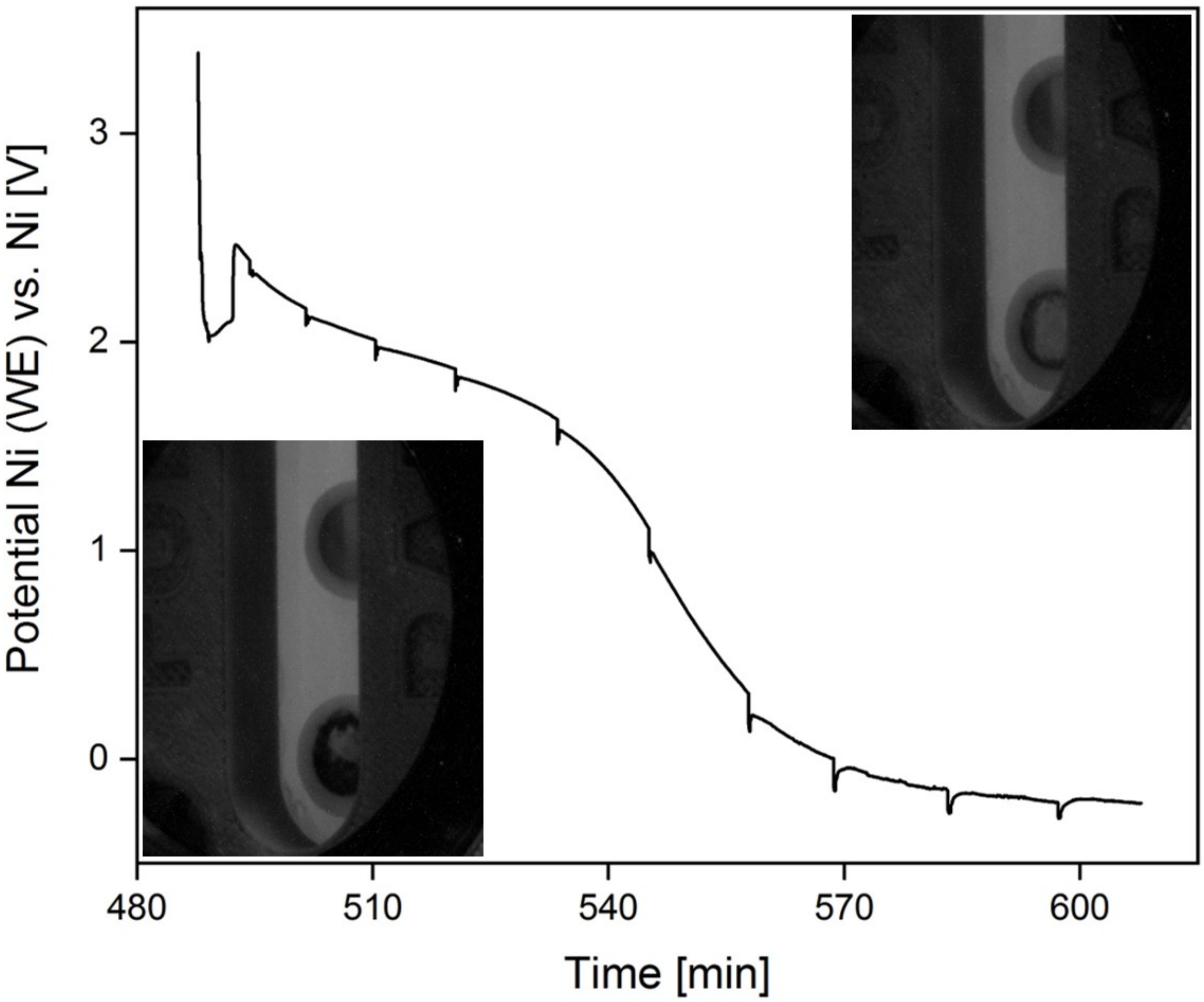

Figure S6: OCV scan of the cell during the in-situ XANES investigation. The time scale refers to the absolute run time of the experiment, including the conditioning phase. The photographs depict the states of the electrodes (top: cathode, bottom: anode) at the start (487 min, left) and the end state (607 min, right). The slowly decreasing residual potential corresponds to the prolonged generation of a thick anodic film and the absence of any reducing agent like H_2O. The potential of the cell reaches a minimum of about −200 mV, which is associated with the formation of films with different thicknesses on anode (thicker) and cathode (thinner). The peaks in the potential-time curve are associated with vibrations of the cell due to the movement of the detector (In order to take photos of the sample, the detector had to be moved away from the measurement position).

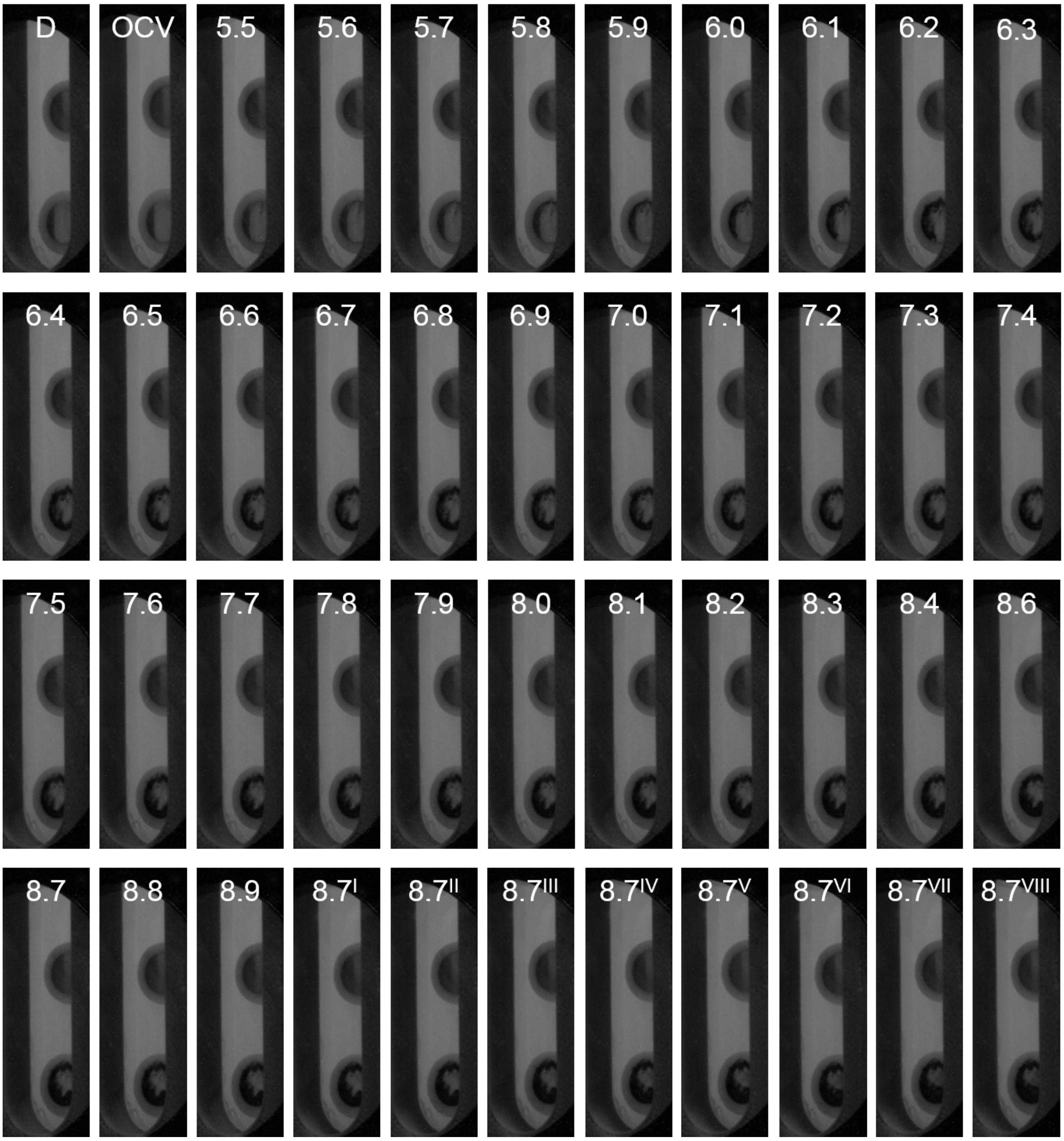

Figure S7: Nickel cathode (top) and anode (bottom) in *a*HF in the electrochemical in-situ XAS cell at stepwise increased cell potentials. D = disconnected, OCV = open circuit voltage, all cell potentials are positive and given in [V]; 8.7^{I} – 8.7^{VIII}: the cell was kept at a constant cell potential of +8.7 V for 127 min and the photos were taken after 40, 54, 64, 77, 90, 103, 115, and 127 min, respectively. The cell potential was increased from +5.5 V to +8.9 V in 0.1 V steps in periods of 10 min and was kept at +8.7 V for an extended conditioning phase of 127 min. The slow formation of the black film started already at +5.5 V and proceeded upon increasing the potential and during the prolonged conditioning period. XANES spectra (Fig. S13 and S14) were measured one minute after applying a new potential with an approximate measurement time of 6 min and the corresponding photos were taken in-between measurements. Throughout the experiment, the cathode remained visibly unchanged.

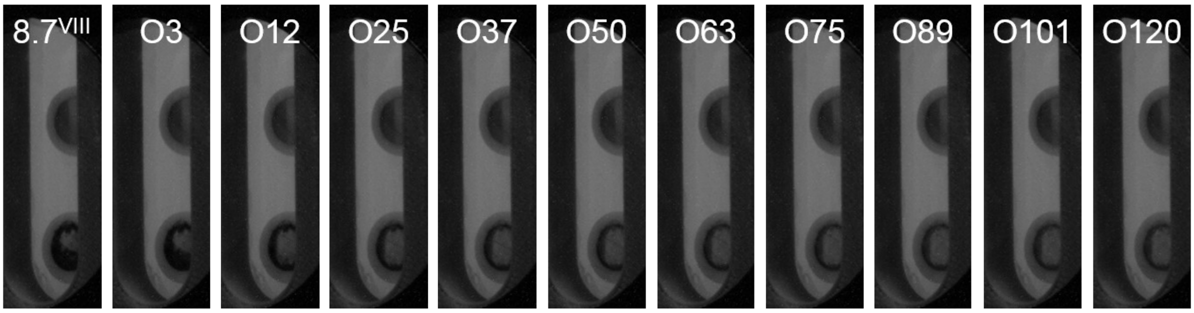

Figure S8: Nickel cathode (top) and anode (bottom) in *a*HF in the electrochemical in-situ XAS cell at a cell potential of +8.7 V (8.7^{VIII}, complete conditioning period) and under open circuit conditions (O3–O120). Photos of the cell during the OCV scan were taken after 3, 12, 25, 37, 50, 63, 75, 89, 101 and 120 min, respectively. The decomposition of the black film on the anode started immediately when the potential applied during the conditioning phase (Fig. S7) was switched off. XANES spectra (Fig. S15 and S16) were measured every 10 min with an approximate measurement time of 6 min. Throughout the experiment, the cathode remained visibly unchanged.

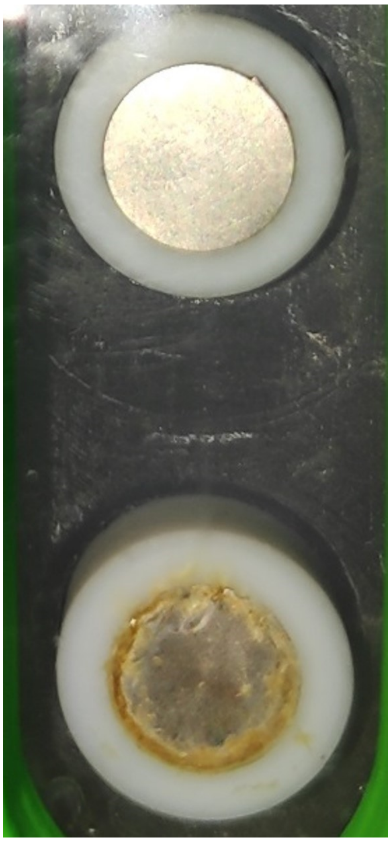

Figure S9: Nickel cathode (top) and anode (bottom) in *a*HF in the electrochemical in-situ XAS cell after decomposition of the black film under open circuit conditions for 120 min. The color of the decomposed film varies between colorless, slightly yellow, and slightly brownish. Throughout the experiment, the cathode remained visibly unchanged.

2.2 Additional in-situ XANES data

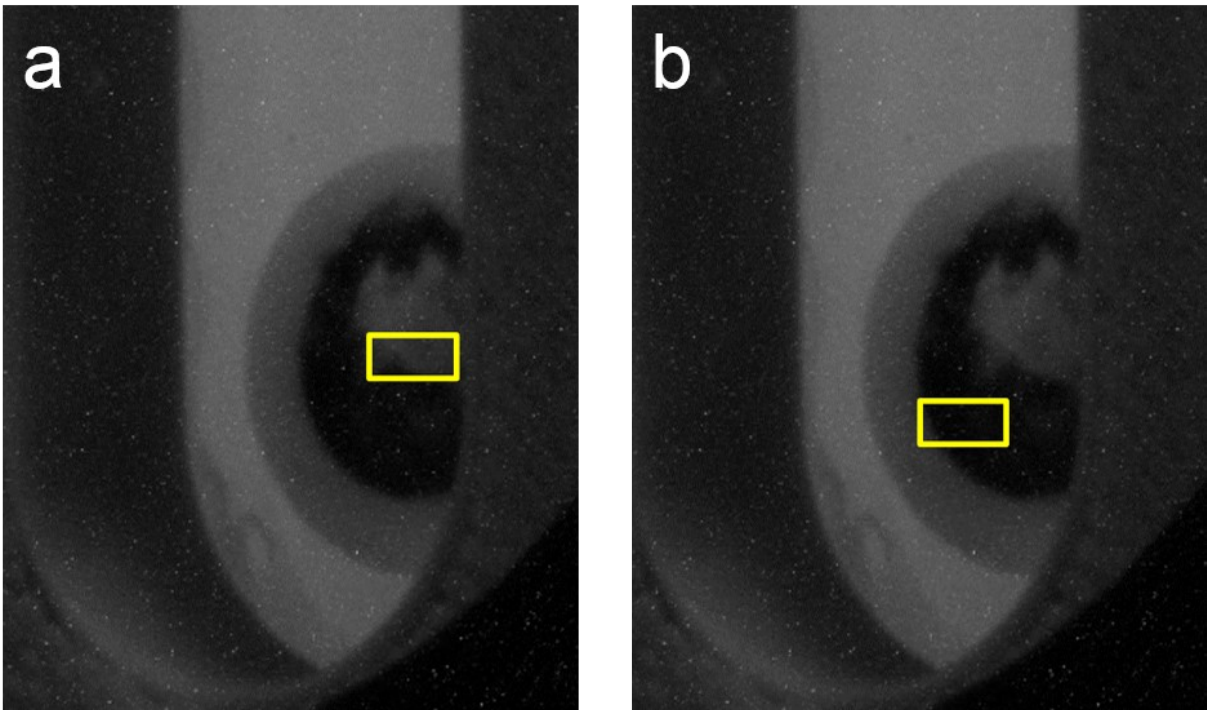

Figure S10: Nickel anode in *a*HF at a cell potential of +8.7 V with the beam spot of the in-situ XAS measurements highlighted (yellow boxes) in a) central and b) signal-optimized positions.

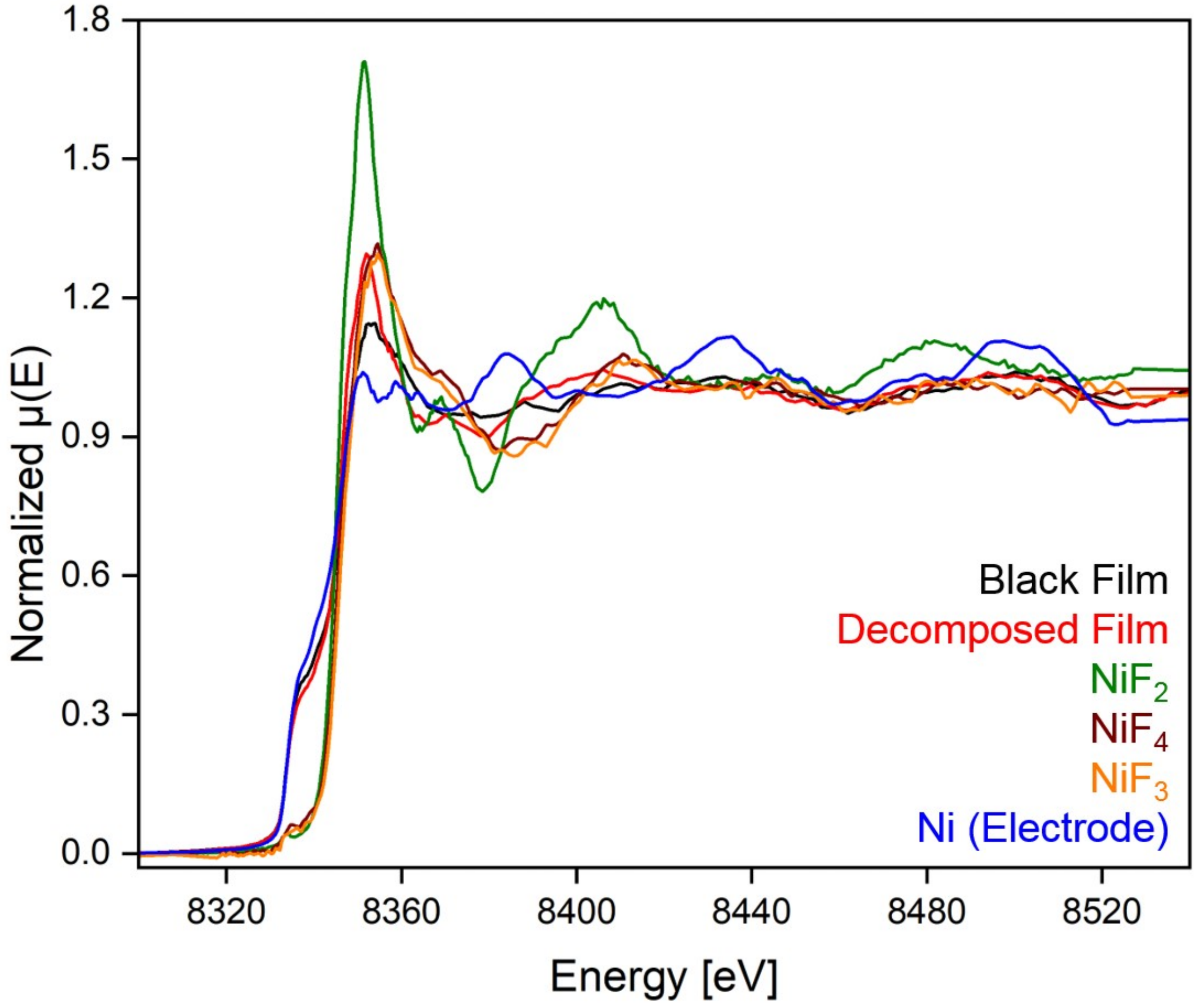

Figure S11: Ni K-edge XANES spectra of the black film (black), the decomposed film (red), and the standards NiF_2 (green), NiF_4 (brown), NiF_3 (orange), and Ni (bulk anode exposed to HF, blue) in the range from 8300 to 8540 eV with an acquisition step size of 0.5 eV.

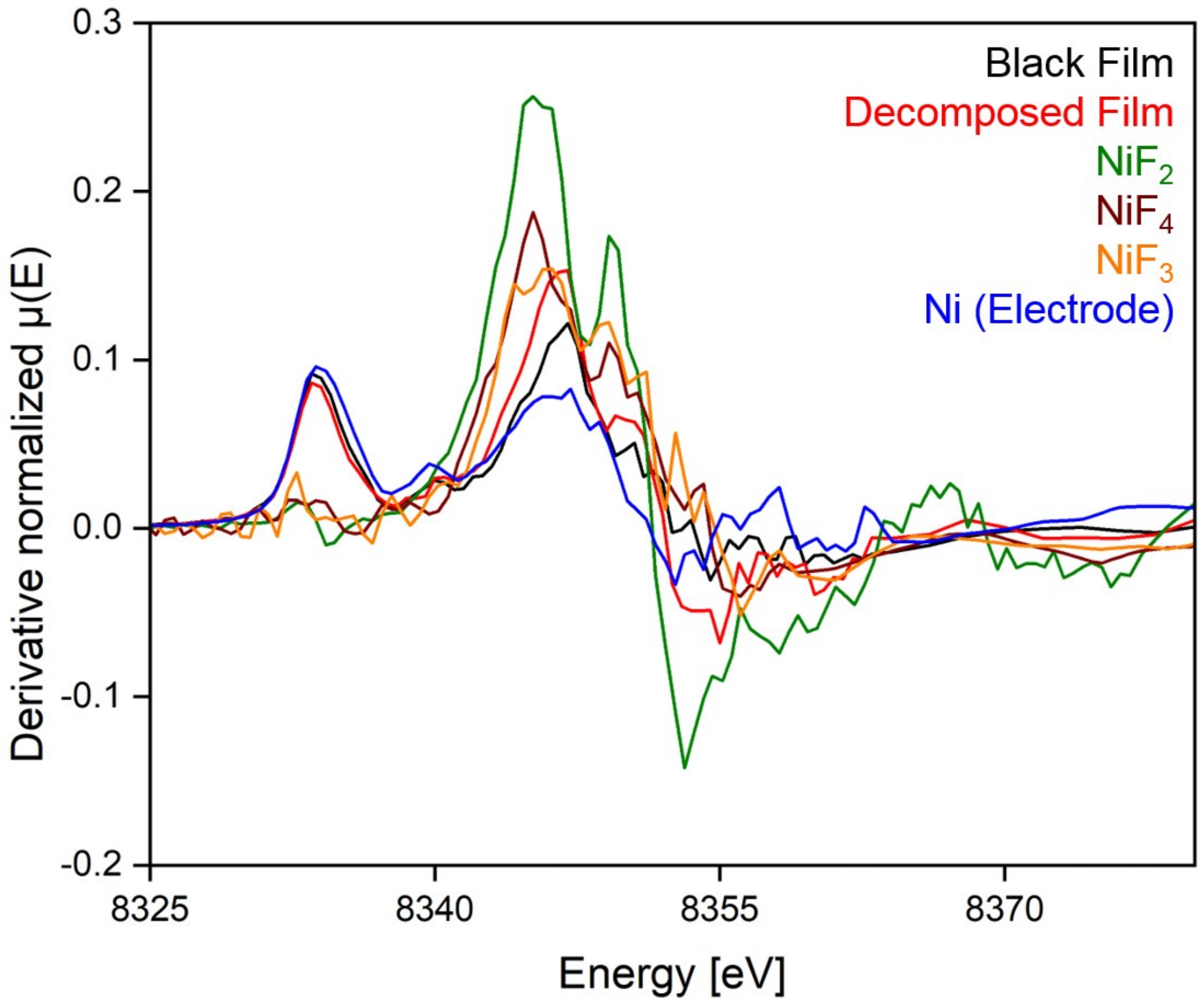

Figure S12: First derivative of Ni K-edge XANES spectra of the black film (black), the decomposed film (red), and the standards NiF_2 (green), NiF_4 (brown), NiF_3 (orange), and Ni (bulk anode exposed to HF, blue) in the range from 8325 to 8380 eV with an acquisition step size of 0.5 eV.

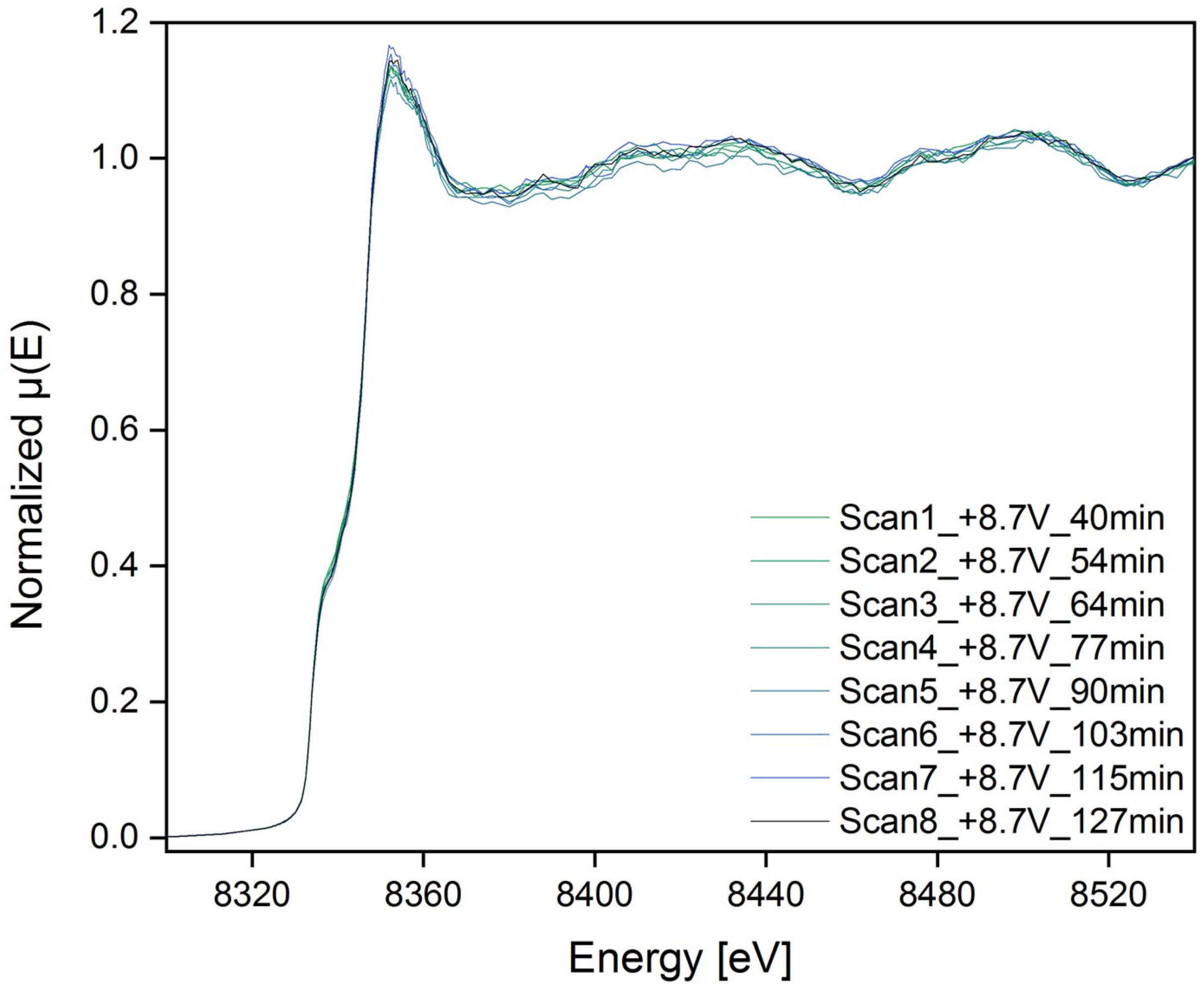

Figure S13: Ni K-edge XANES spectra of the development of the black film during the prolonged conditioning phase at a cell potential of +8.7 V (Fig. S7) in the range from 8300 to 8540 eV with an acquisition step size of 0.5 eV. The spectrum "Scan8_+8.7V_127min" corresponds to the "Black Film" in the Figures 3, S11 and S12. The small changes observed lie within the error range.

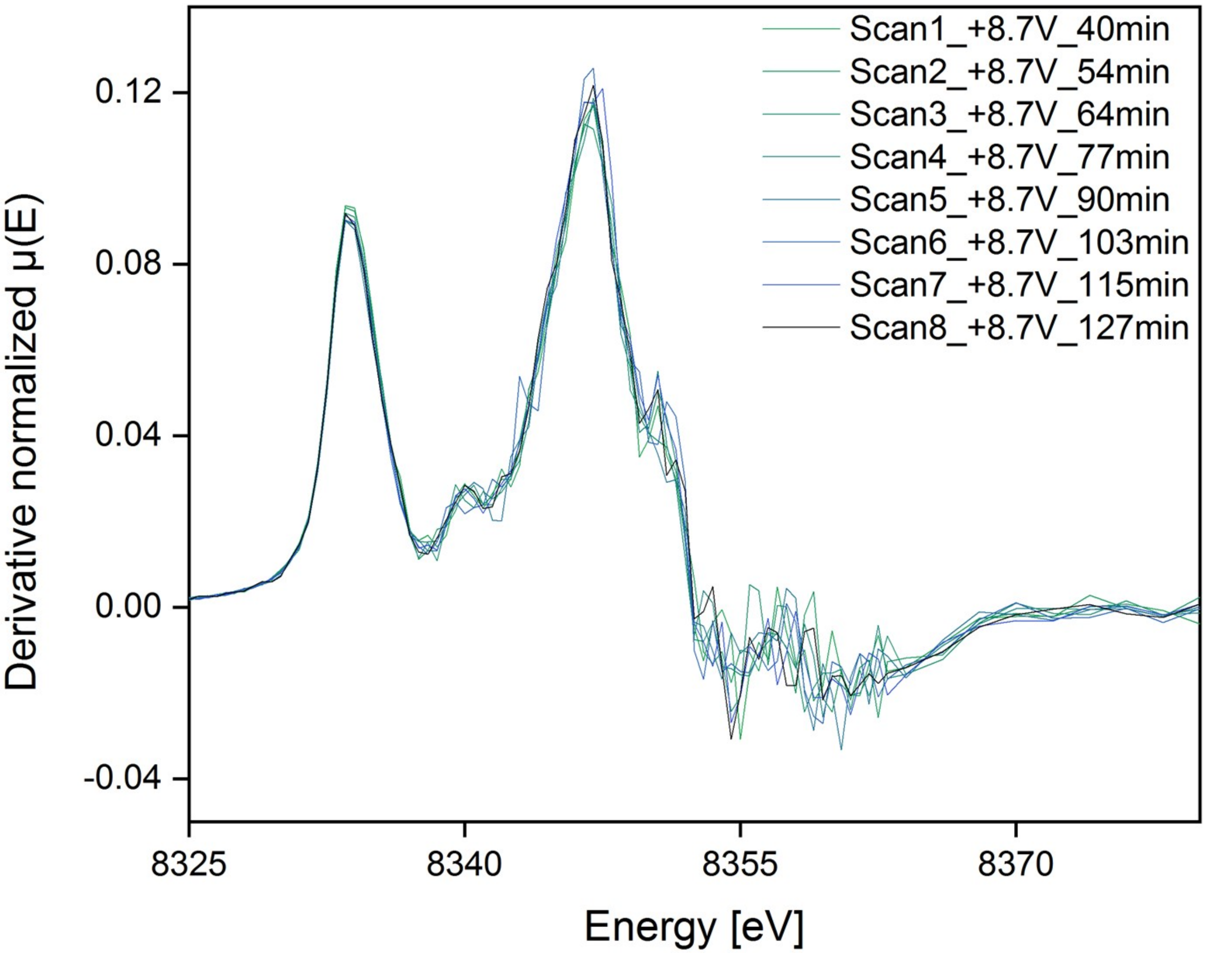

Figure S14: First derivative of Ni K-edge XANES spectra of the development of the black film during the prolonged conditioning phase at a cell potential of +8.7 V (Fig. S7) in the range from 8325 to 8380 eV with an acquisition step size of 0.5 eV. The spectrum "Scan8_+8.7V_127min" corresponds to the "Black Film" in the Figures 3, S11 and S12. The small changes observed lie within the error range.

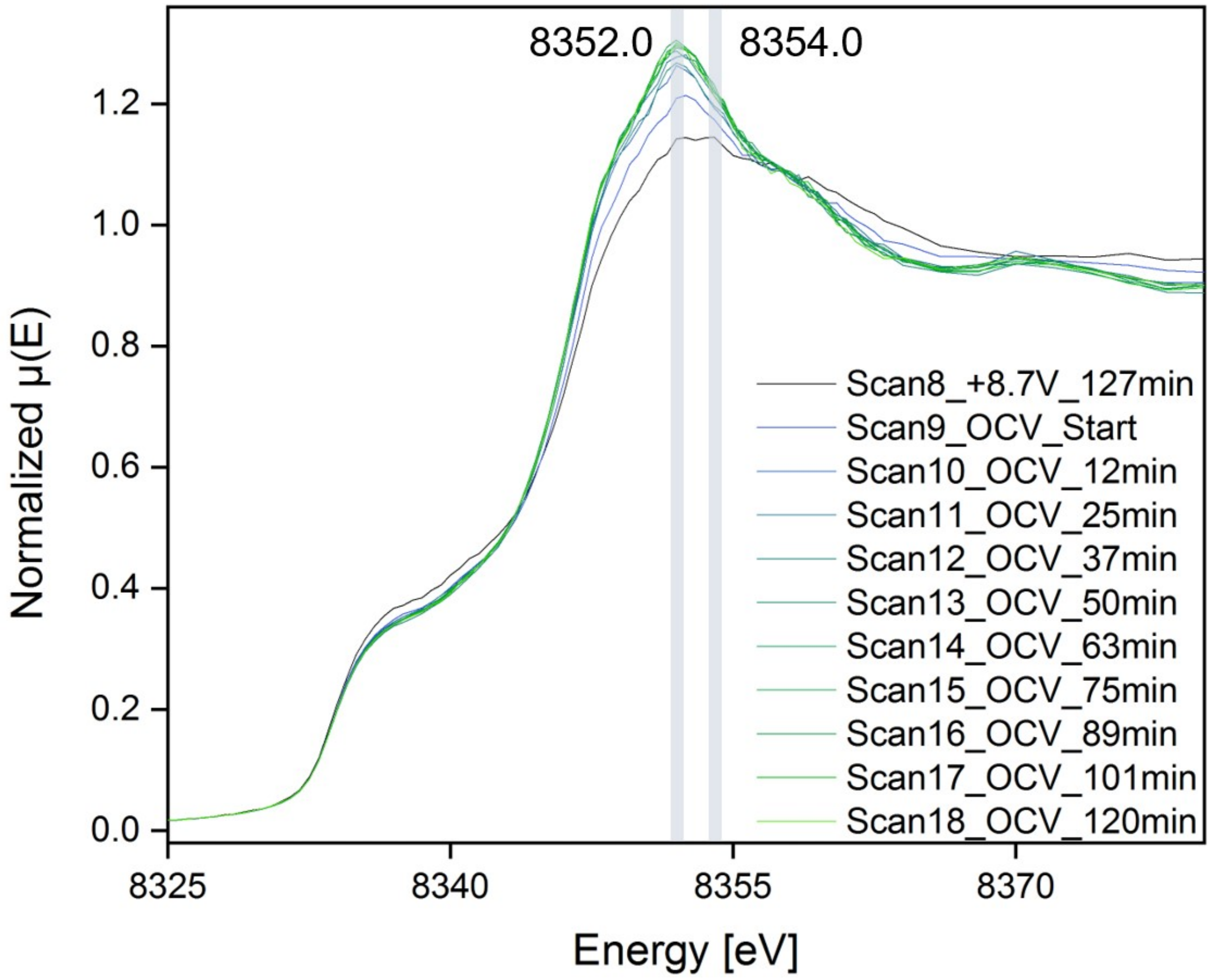

Figure S15: Ni K-edge XANES spectra of the decomposition of the black film at open circuit conditions (Fig. S8) in the range from 8325 to 8380 eV with an acquisition step size of 0.5 eV. The spectrum "Scan8_+8.7V_127min" corresponds to the "Black Film" and the spectrum "Scan18_OCV_120min" refers to the "Decomposed Film" in the Figures 3, S11 and S12, respectively. A redshift of the white line is accompanied by a remarkable increase in its intensity, while also the intensity in the shoulder at 8333 eV decreases and a feature at 8370 eV forms. These observations correspond to the synproportionation of high-valent nickel and Ni^0 and the coincident formation of NiF_2.

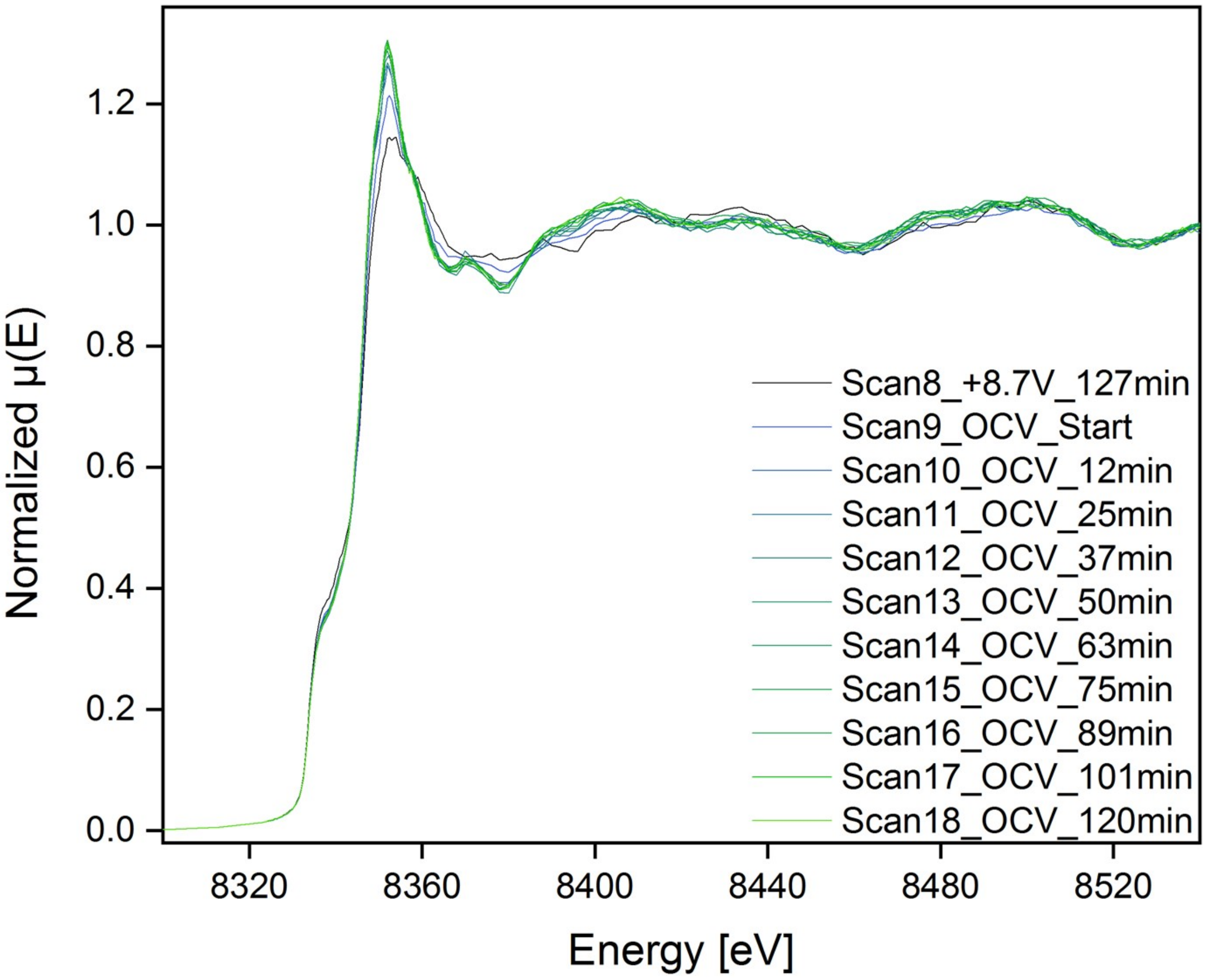

Figure S16: Ni K-edge XANES spectra of the decomposition of the black film at open circuit conditions (Fig. S8) in the range from 8300 to 8540 eV with an acquisition step size of 0.5 eV. The spectrum "Scan8_+8.7V_127min" corresponds to the "Black Film" and the spectrum "Scan18_OCV_120min" refers to the "Decomposed Film" in the Figures 3, S11 and S12, respectively. A shift of the white line is accompanied by a remarkable increase in its intensity, while also the intensity in the shoulder at 8333 eV decreases and a feature at 8370 eV forms. These observations correspond to the synproportionation of high-valent nickel and Ni^0 and the coincident formation of NiF_2.

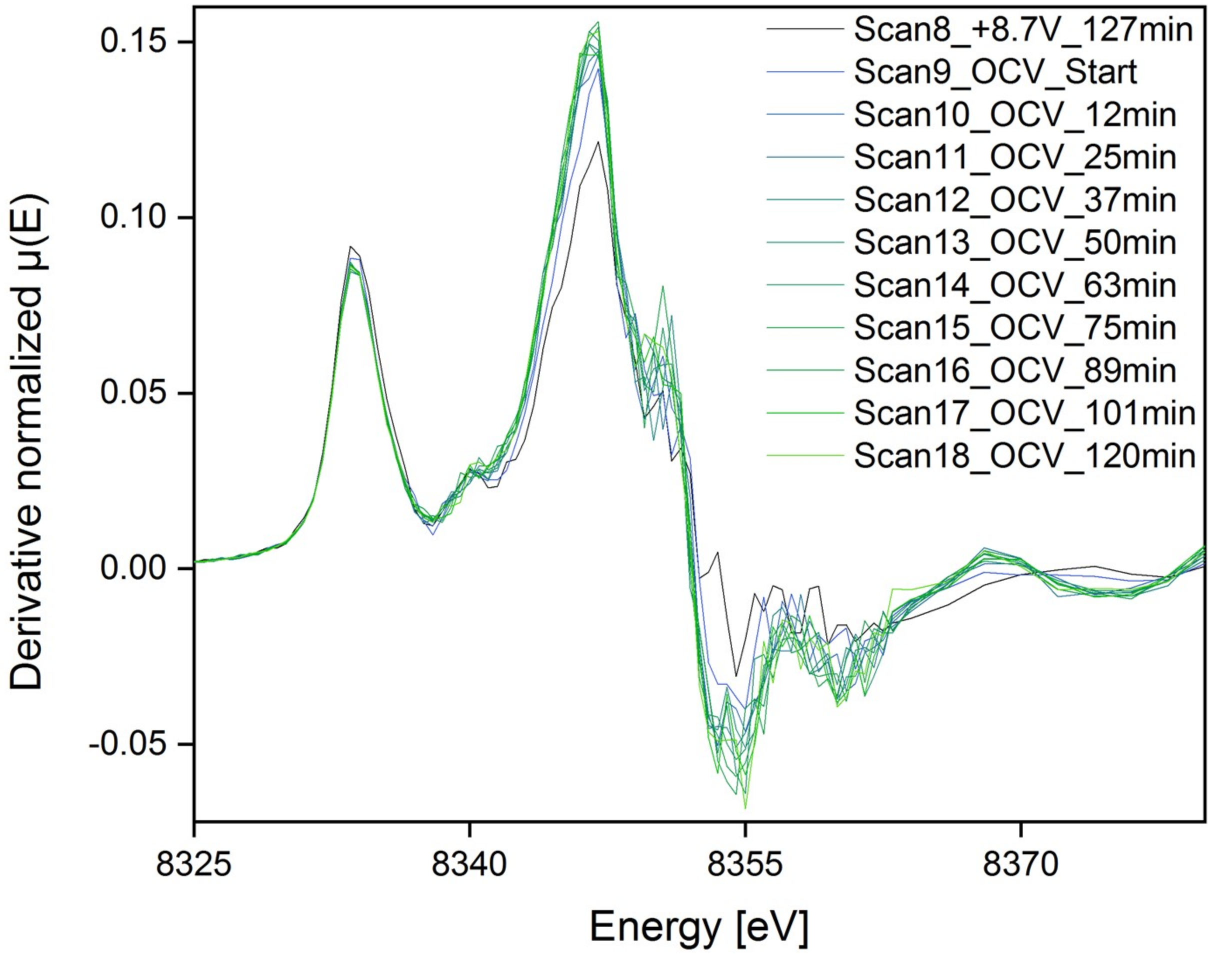

Figure S17: First derivative of Ni K-edge XANES spectra of the decomposition of the black film at open circuit conditions (Fig. S8) in the range from 8325 to 8380 eV with an acquisition step size of 0.5 eV. The spectrum "Scan8_+8.7V_127min" corresponds to the "Black Film" and the spectrum "Scan18_OCV_120min" refers to the "Decomposed Film" in the Figures 3, S11 and S12, respectively.

2.3 Linear combination fitting of the in-situ XANES data

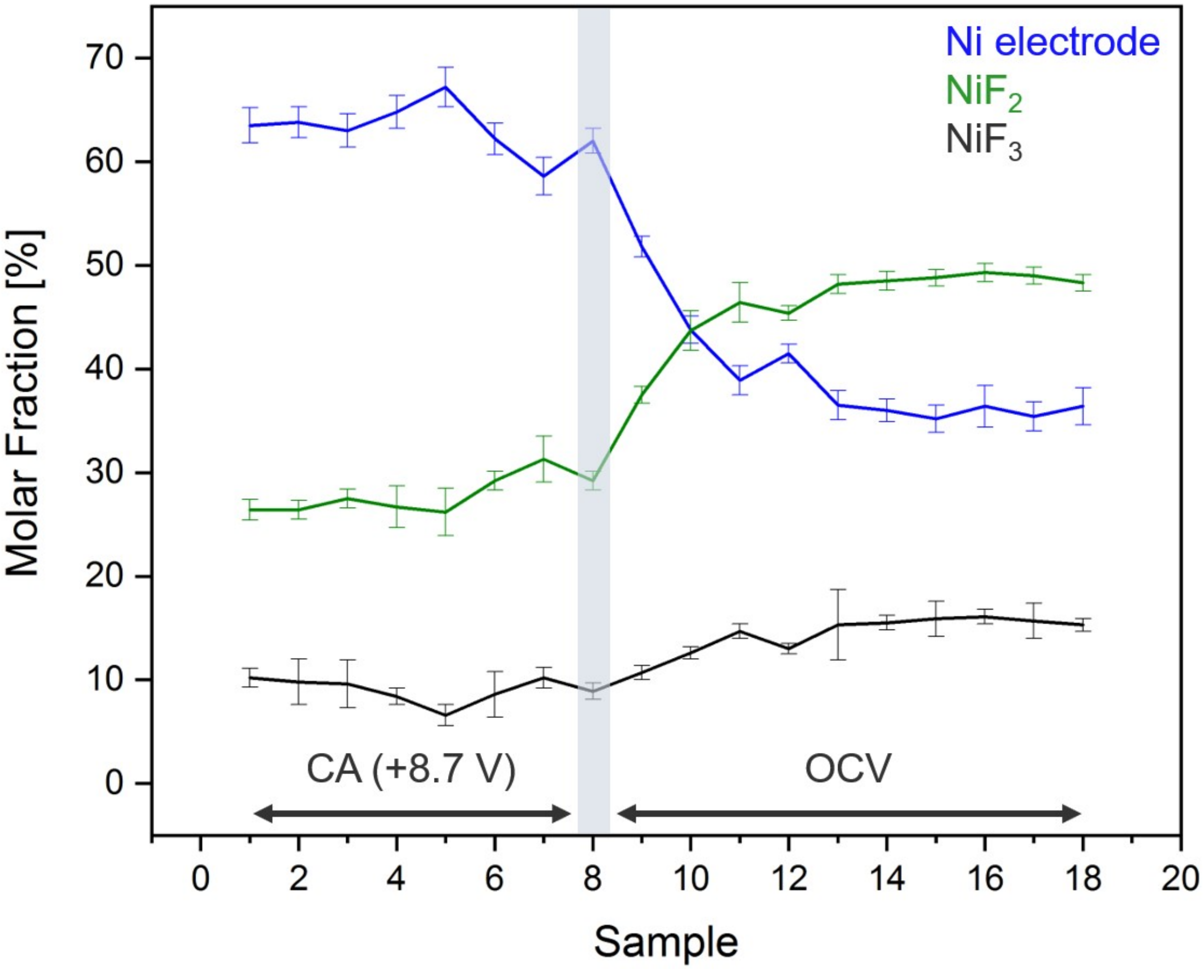

Figure S18: Development of molar fractions on the anode obtained by linear combination fits using the three standards Ni (bulk electrode exposed to HF, blue), NiF_2 (green) and NiF_3 (black). The underlying XANES spectra were recorded during the prolonged conditioning period (CA at a cell potential of +8.7 V, samples 1–8, Fig. S13 and S14) and the decomposition of the black film under open circuit conditions (samples 9–18, Fig. S15-S17). After switching off the external potential (measuring OCV) the thickness of the nickel fluoride layer increased significantly, as it can be concluded by the lower contribution of elemental nickel to the XANES spectrum due to the absorption of the nickel fluoride species. Additionally, the ratio between NiF_2 and NiF_3 increases from 2.6:1 during the CA to 3.2:1 after the external potential has been switched off for 120 min. The development of the molar fractions reflects the spontaneous reduction of the active species. Ni^0 is oxidized by means of a synproportionation reaction by the remaining high-valent nickel centers to Ni^{II}.

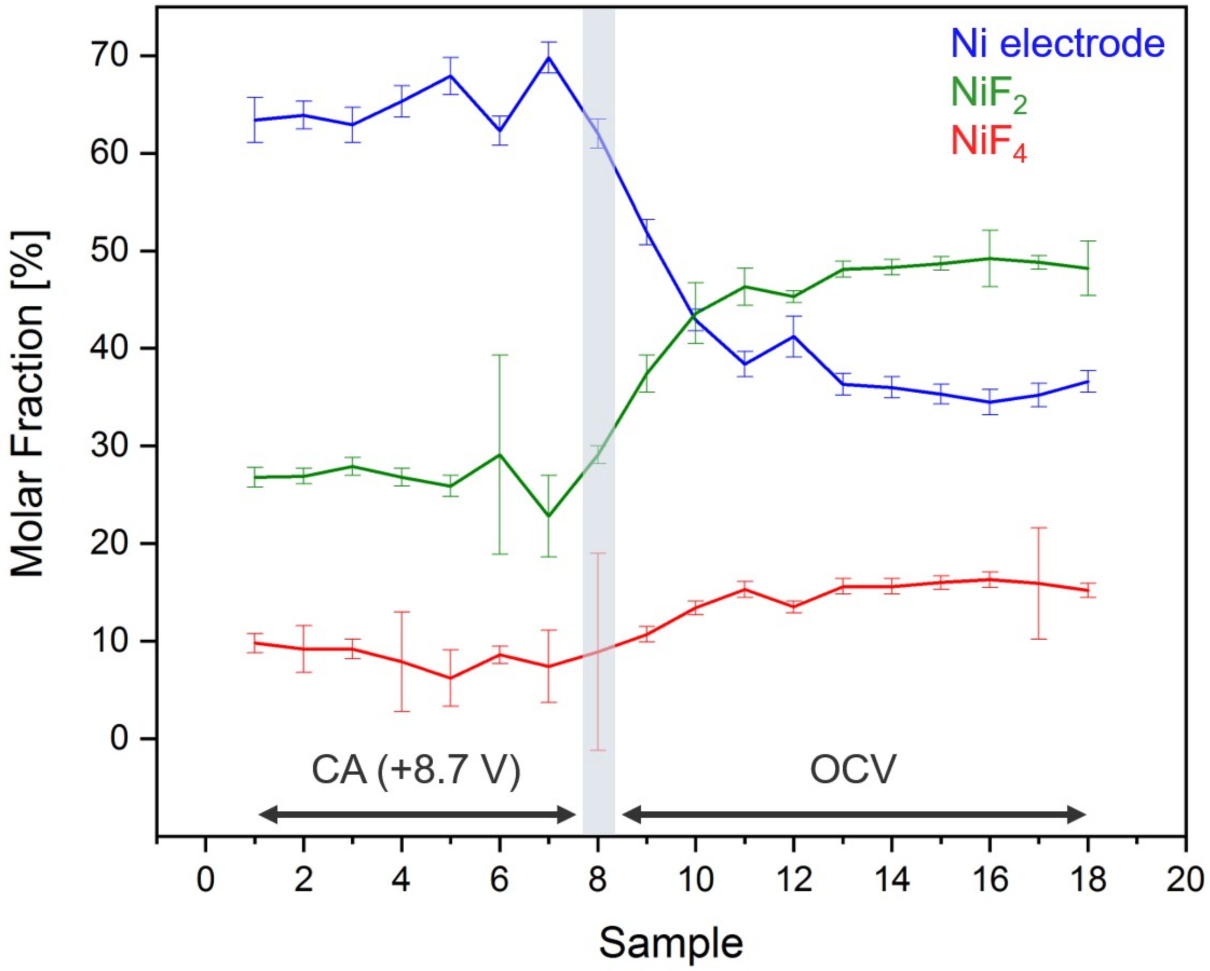

Figure S19: Development of molar fractions on the anode obtained by linear combination fits using the three standards Ni (bulk electrode exposed to HF, blue), NiF_2 (green) and NiF_4 (red). The underlying XANES spectra were recorded during the prolonged conditioning period (CA at a cell potential of +8.7 V, samples 1–8, Fig. S13 and S14) and the decomposition of the black film under open circuit conditions (samples 9–18, Fig. S15-S17). The larger error bars compared to Figure S18 render this fit not suitable for the description of the decomposition of the black film.

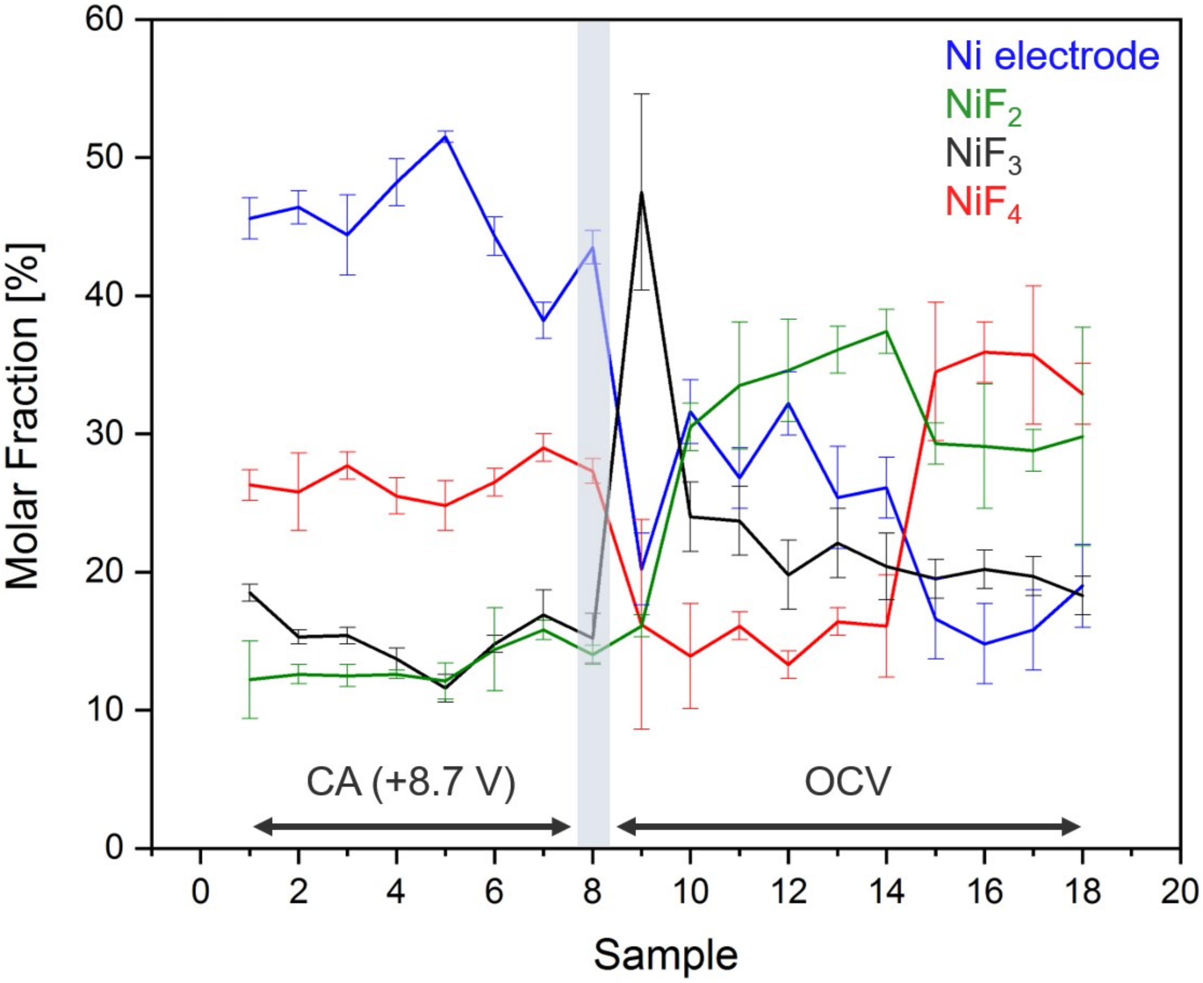

Figure S20: Development of molar fractions on the anode obtained by linear combination fits using the four standards Ni (bulk electrode exposed to HF, blue), NiF_2 (green), NiF_3 (black) and NiF_4 (red). The underlying XANES spectra were recorded during the prolonged conditioning period (CA at a cell potential of +8.7 V, samples 1–8, Fig. S13 and S14) and the decomposition of the black film under open circuit conditions (samples 9–18, Fig. S15-S17). The development of the molar fractions reflects the spontaneous decomposition of high-valent nickel fluorides and reduction to NiF_2 at open circuit conditions, the overall increasing NiF_2 and decreasing Ni (electrode) amounts, since Ni^0 is oxidized by means of synproportionation reaction by the remaining high-valent nickel centers to NiF_2. The 're-increasing' NiF_4 fraction in samples 15 to 18 is considered wrong and the overall higher error ranges render the four-standard LCF not suitable in the description of the decomposition of the black film.

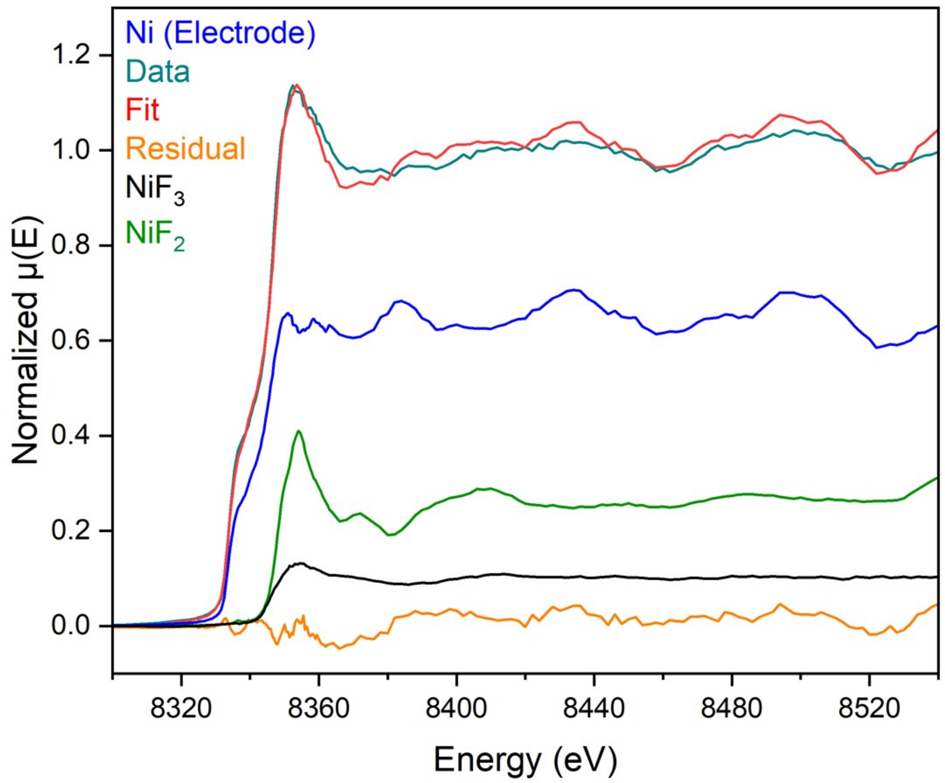

Figure S21: Linear combination fitting (red) of the XANES spectrum of sample 1 (turquoise, cf. Fig. S13 and S18) to the reference standards Ni (blue), NiF_2 (green), NiF_3 (black) and the residual (orange).

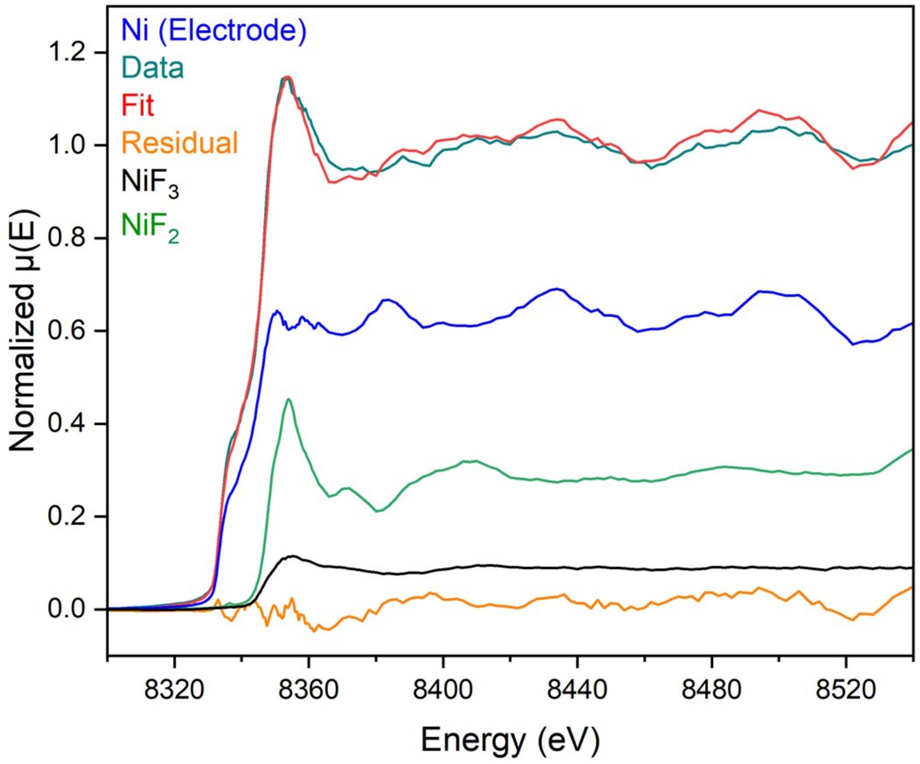

Figure S22: Linear combination fitting (red) of the XANES spectrum of sample 8 (turquoise, cf. Fig. S13, S15 and S18) to the reference standards Ni (blue), NiF_2 (green), NiF_3 (black) and the residual (orange).

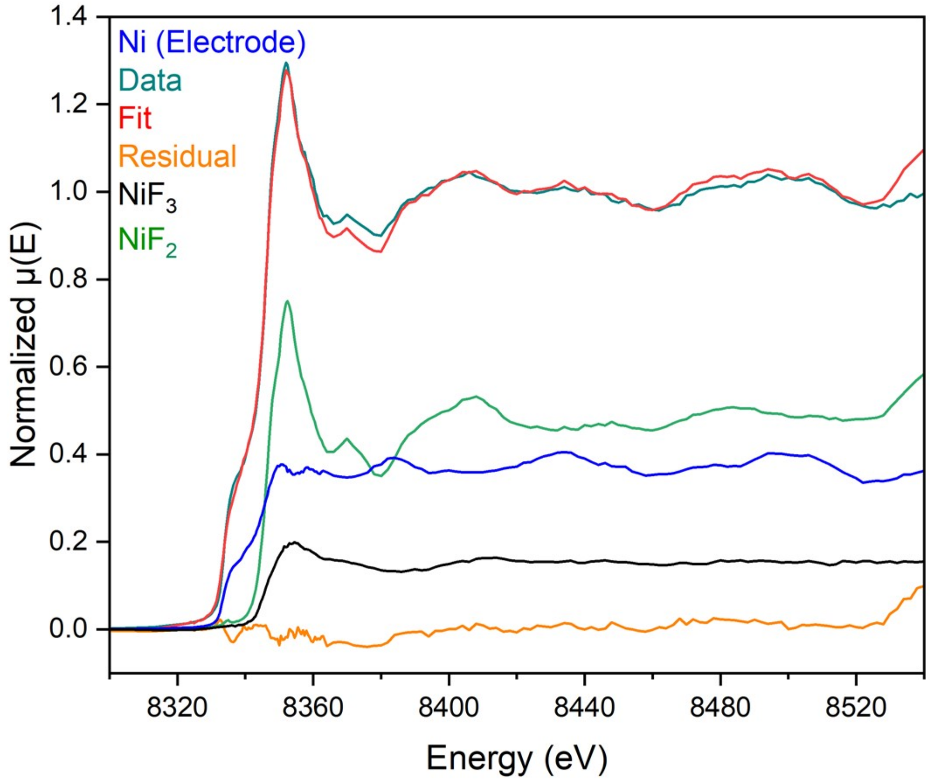

Figure S23: Linear combination fitting (red) of the XANES spectrum of sample 18 (turquoise, cf. Fig. S15 and S18) to the reference standards Ni (blue), NiF_2 (green), NiF_3 (black) and the residual (orange).

3 Ex-situ characterization and standards

3.1 Generation and decomposition of a black film (ex-situ)

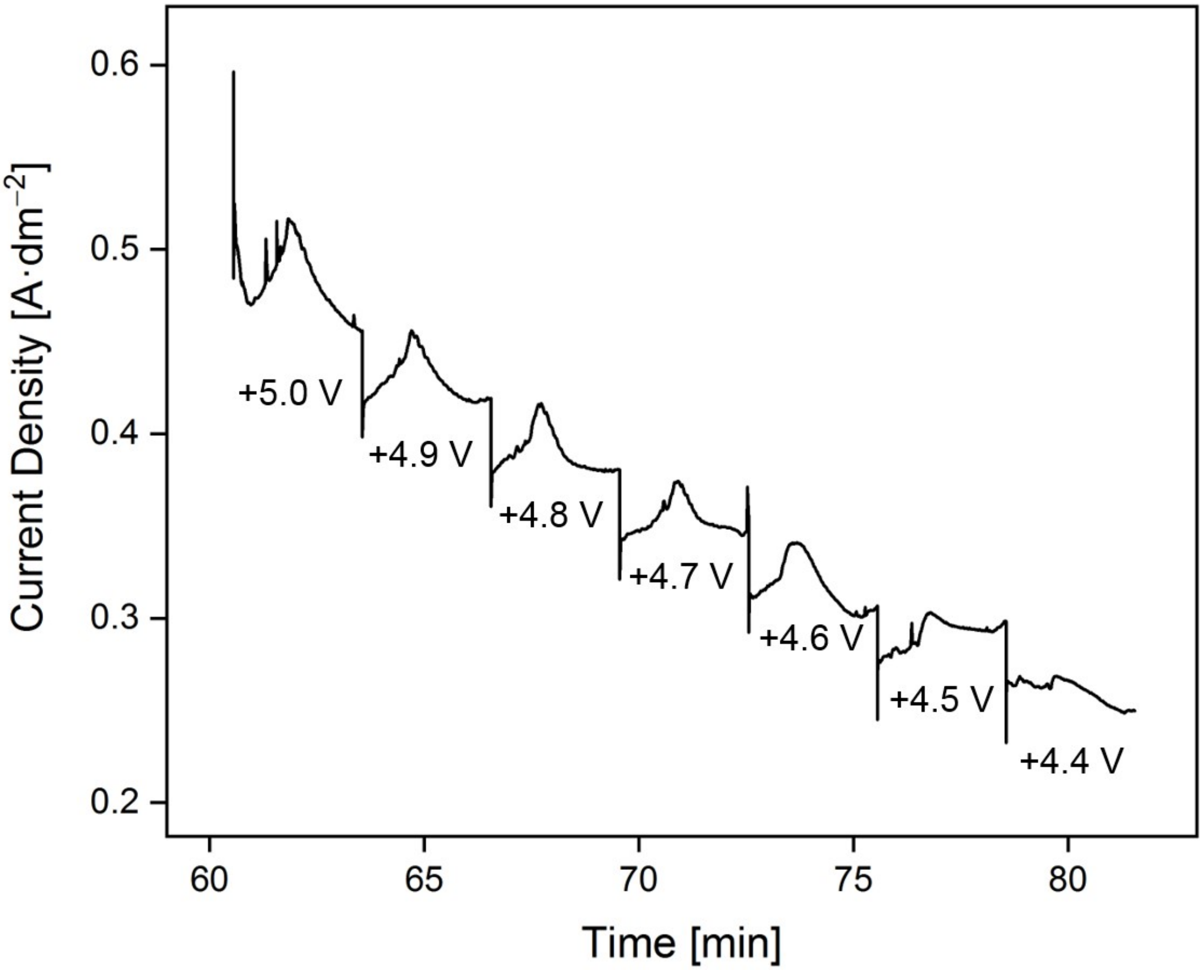

Figure S24: Chronoamperogram with the variation of the cell potential from +5.0 V to +4.4 V in 0.1 V steps, each applied for 3 min, recorded after a conditioning phase of 60 min at a cell potential of +6.0 V. The potential steps between +5.0 and +4.5 V show a current density peak, which is associated with a re-oxidation of the active film after a potential change. No such current density peak was observed at a cell potential of +4.4 V. The time scale refers to the absolute run time of the experiment, including the conditioning phase (see also Fig. S25).

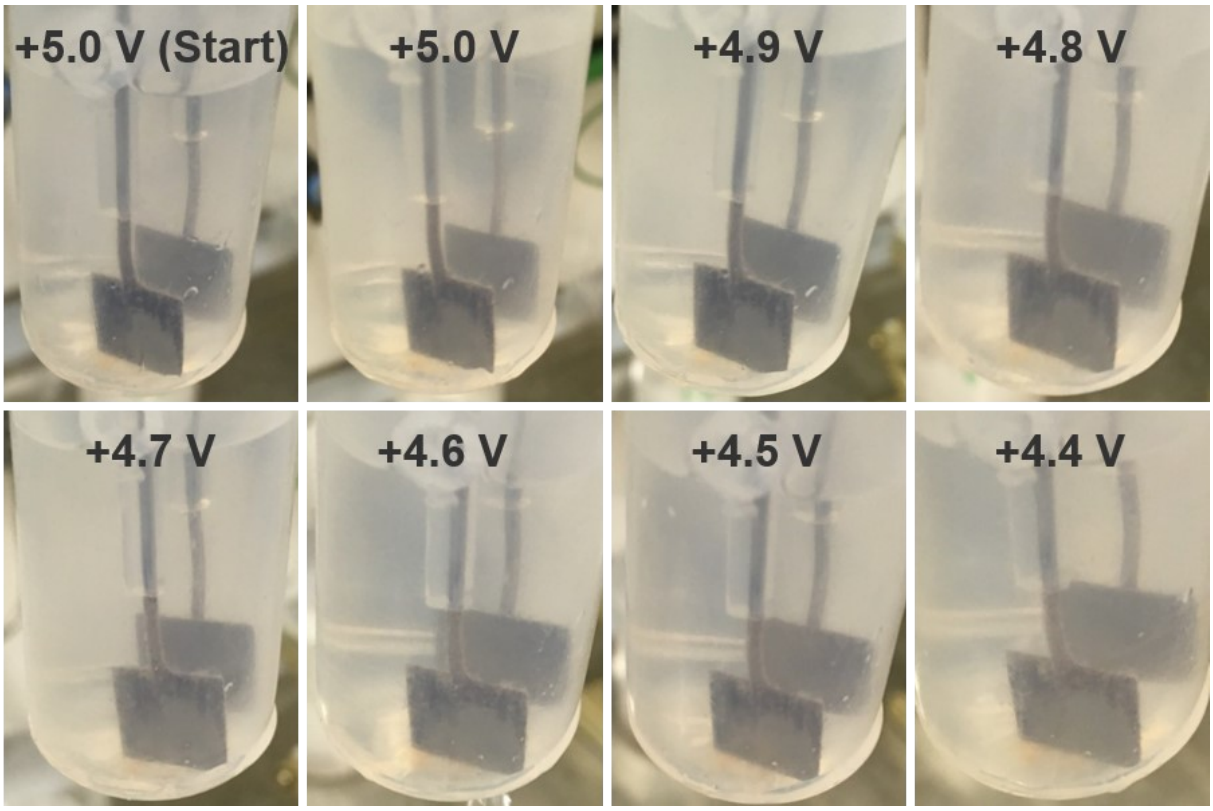

Figure S25: The color development of the anode (front) in *a*HF shows that the blackness gradually decreases upon stepwise lowering of the potential. At +4.4 V, no black color was observed anymore, which coincides with the absence of a peak in current density (Fig. S24). The photographs were taken at the end of each potential step.

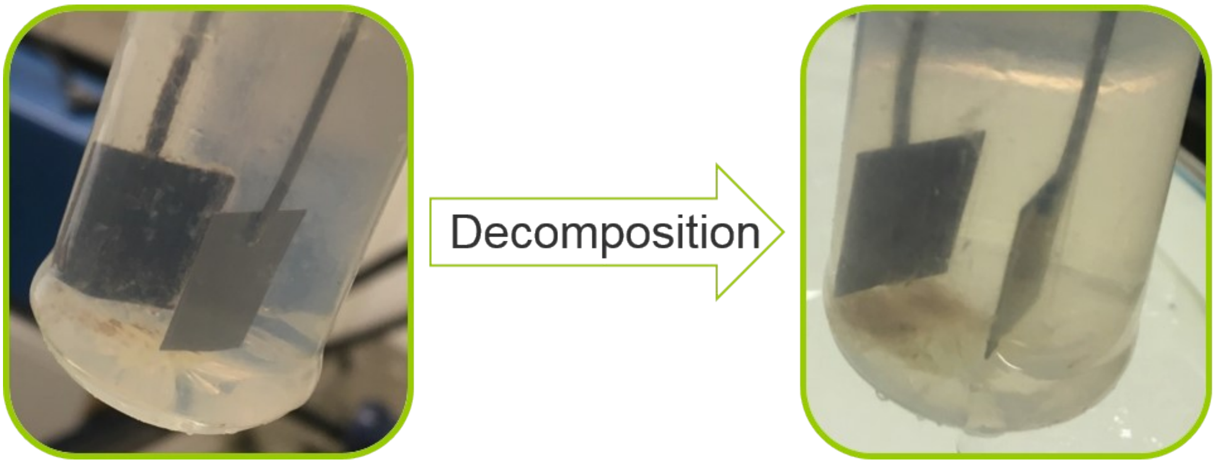

Figure S26: Nickel electrodes in liquid *a*HF for sample preparation for SEM and ex-situ XANES investigations after conditioning for 120 min at a cell potential of +6.0 V (left) and after decomposition at open circuit conditions (right). The anode, which is depicted on the left in both photos, loses substantial amounts of NiF_2 upon decomposition, which is found on the ground of the cell.

3.1.1 PXRD data of a decomposed film

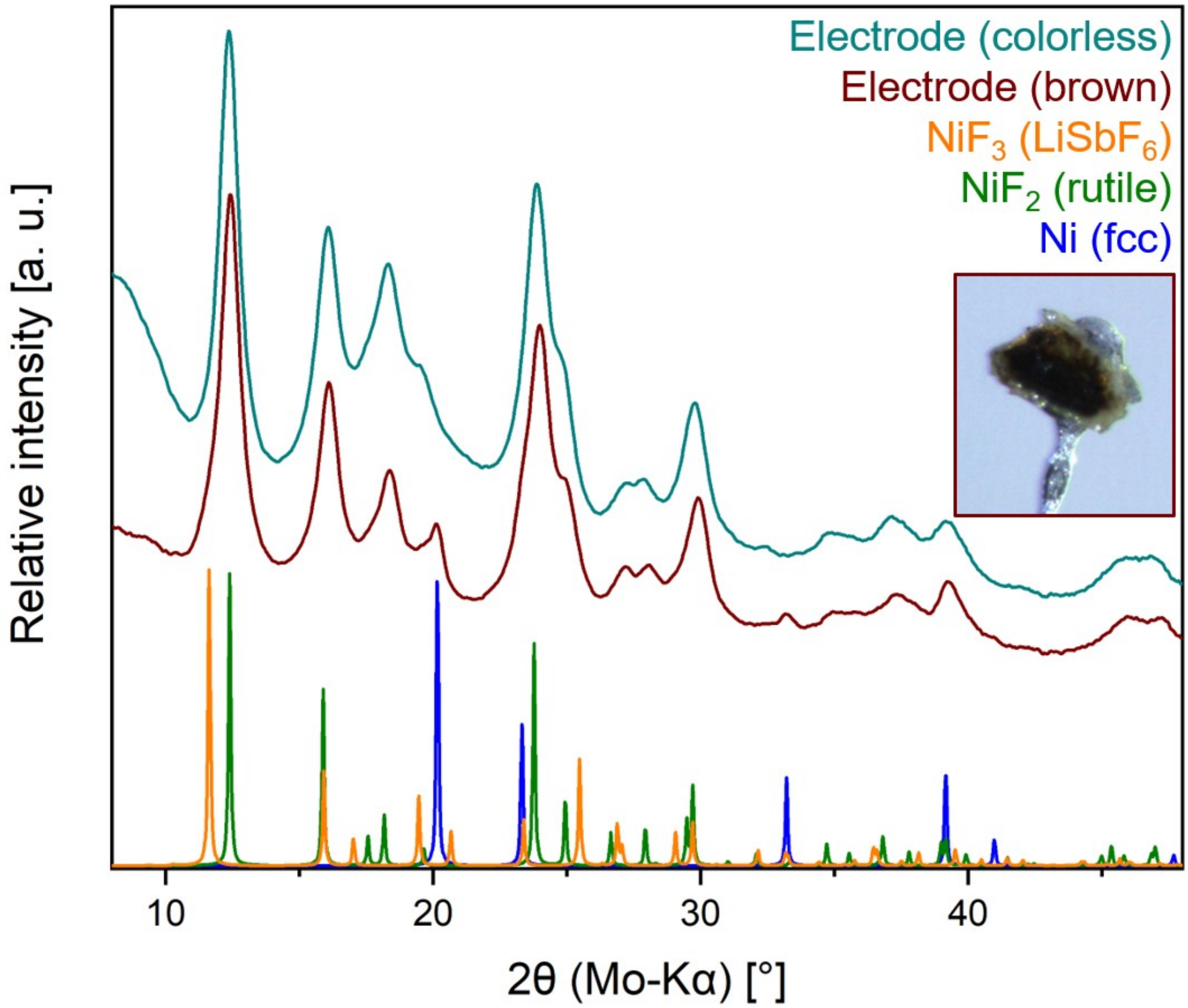

Figure S27: Powder X-ray diffractograms (PXRD) of colorless (turquoise) and brown (brown, photo) fragments found on the decomposed surface of a nickel anode prepared under conditions of the Simons process in comparison with simulated data of NiF_3 ($LiSbF_6$ type, orange)[5], NiF_2 (rutile, green)[8] and Ni (fcc, blue)[9]. Regardless of the color (colorless or brown), only NiF_2 and traces of elemental nickel were found on the surface. The PXRD samples were produced as follows: After the generation of a black film at ϑ = 0 °C, the power supply was switched off and the electrode (a nickel wire) was removed from the *a*HF, which had been cooled to ϑ < −60 °C by the end of the production of the black film, and immersed into perfluorinated ether oil (ϑ < −60 °C) three minutes after the system was disconnected from the power supply. Fragments were scratched off the electrode using a scalpel and subjected to PXRD measurements.

3.1.2 SEM images of a decomposed film

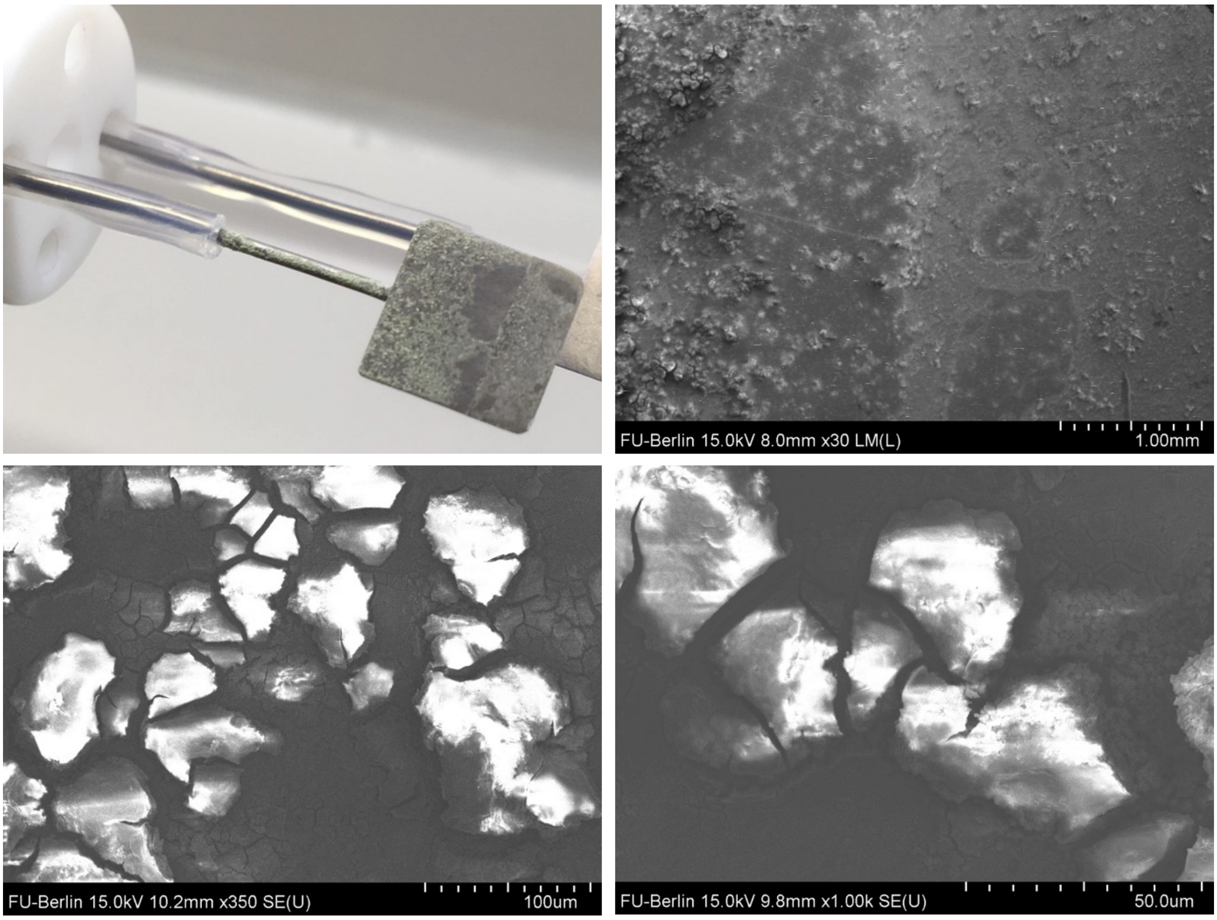

Figure S28: Scanning electron microscopy images of a nickel anode with a black film generated at a cell potential of +6.0 V for 120 min in liquid *a*HF and decomposed afterwards under open circuit conditions revealing particles averagely sized 50–100 µm. In order to create a visible step between passivated and non-passivated surface, the lower 30 % of the electrode was covered with PTFE tape. The partial decomposition of the PTFE during the conditioning phase led to gas evolution and thereby unveiling the larger central areas with metallic luster.

3.1.3 Ex-situ XANES data of a decomposed film

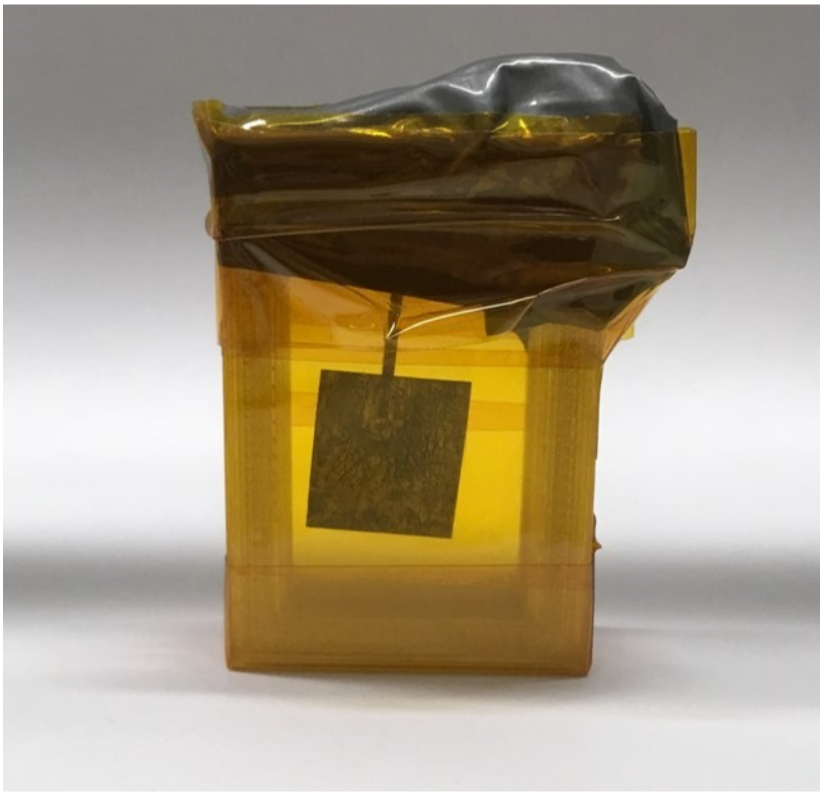

Figure S29: Decomposed anodic film on a nickel plate electrode in a 3D-printed sample holder for XANES measurements in fluorescence covered with 125 µm-Kapton® foil. The black film had been generated at a cell potential of +6.0 V for 120 min in liquid *a*HF and decomposed in air (Section **1.1.1**).

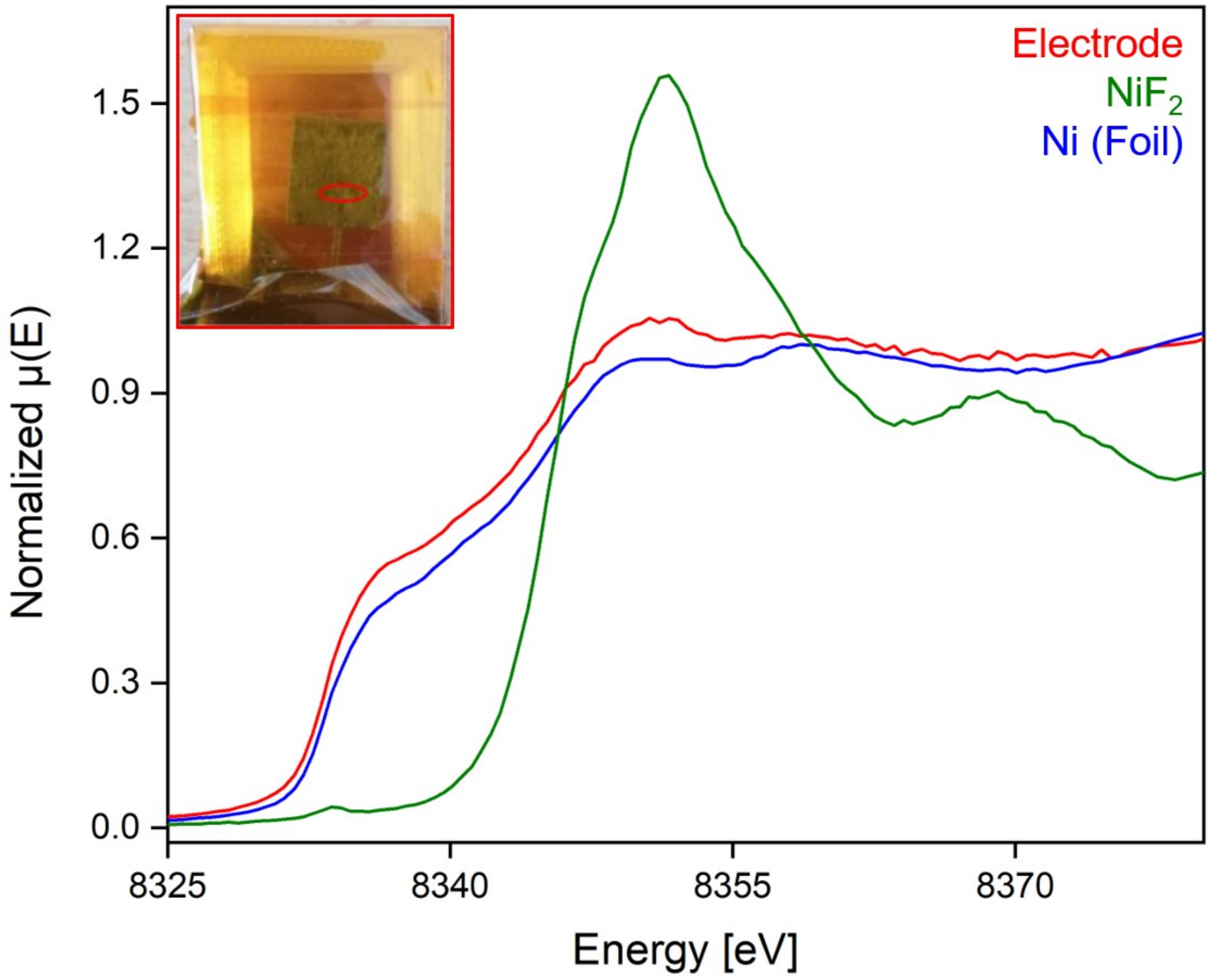

Figure S30: Ni K-edge XANES spectra of a decomposed nickel electrode (purple, photo with the measurement position highlighted in red) and standards NiF_2 (green), and Ni (foil, transmission measurement, turquoise) in the range from 8325 to 8380 eV at a spectral resolution of 0.5 eV.

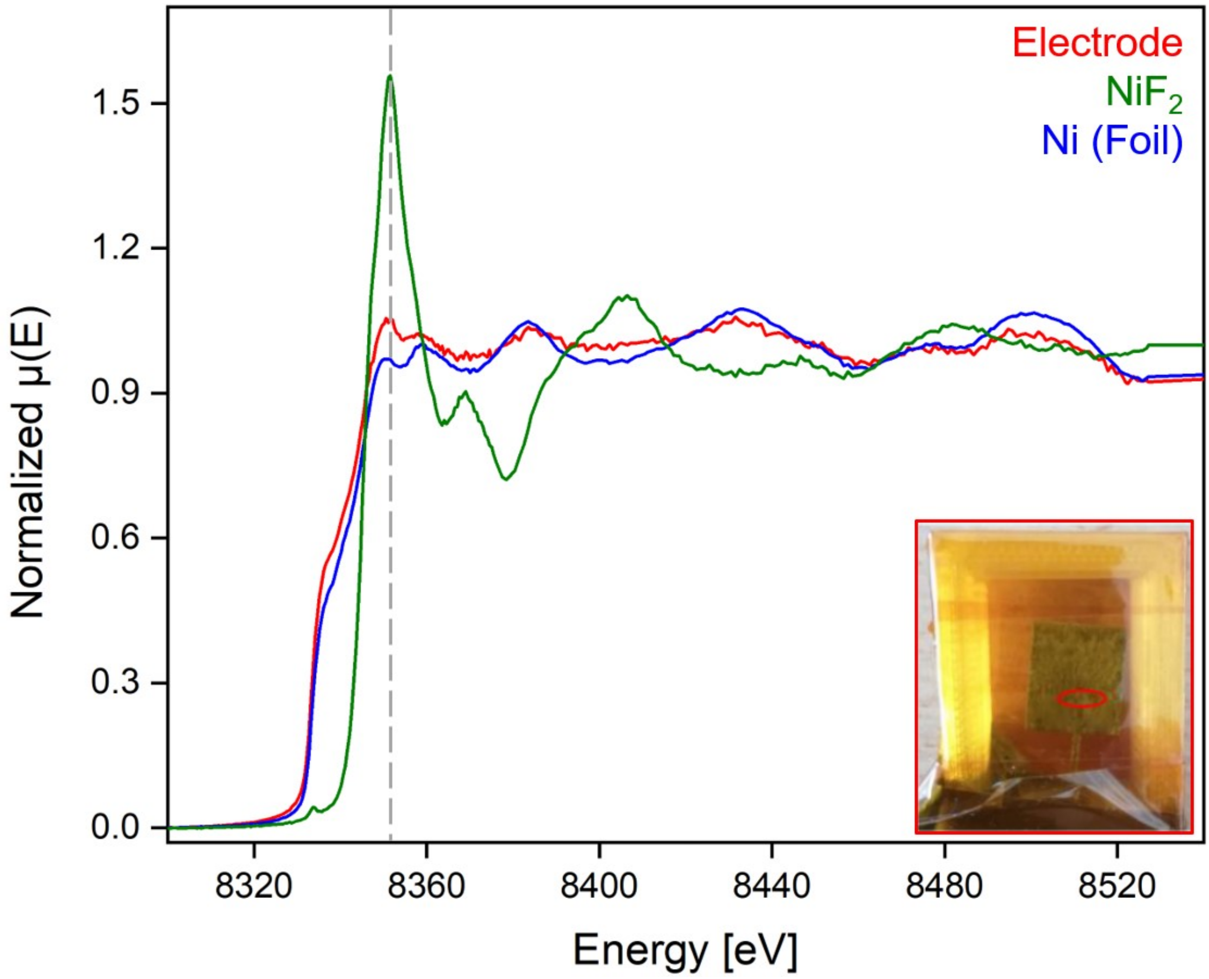

Figure S31: Ni K-edge XANES spectra of a decomposed nickel electrode (purple, photo with the measurement position highlighted in red) and standards NiF_2 (green), and Ni (foil, transmission measurement, turquoise) in the range from 8300 to 8540 eV at a spectral resolution of 0.5 eV. The maximum of absorbance of NiF_2 at 8351.6 eV was highlighted (grey dashed line).

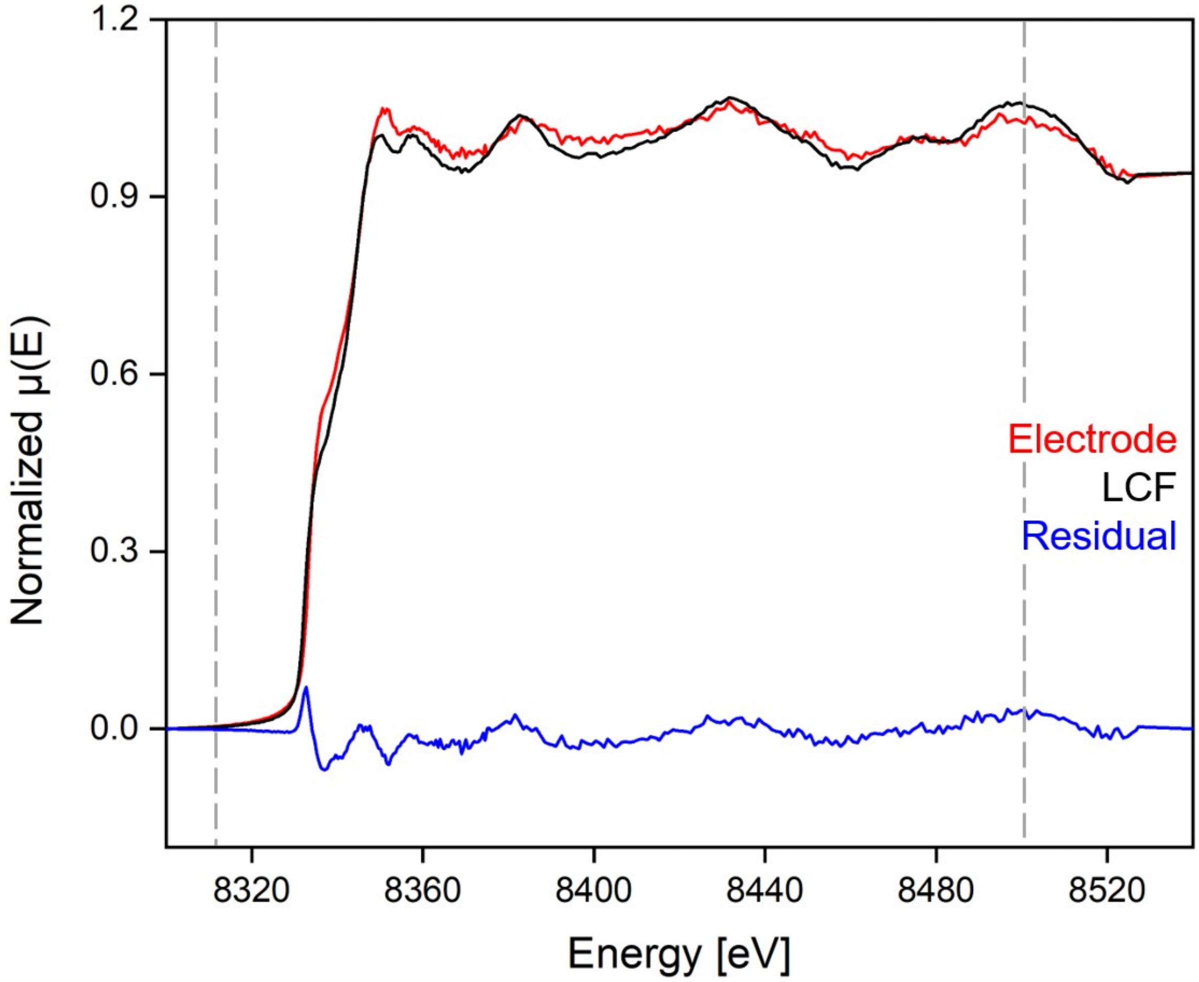

Figure S32: Ni K-edge XANES spectrum of a decomposed nickel electrode (red) and a linear combination fit using the standards NiF_2 and Ni (black, see also Fig. S31) as well as the difference of both (residual, blue) in the range from 8300 to 8540 eV at a spectral resolution of 0.5 eV. The region of the fit (8313.21 to 8503.21 eV) was highlighted (grey dashed lines). The mass fractions were found to be 4.2 % (± 0.9 %) for NiF_2 and 95.8 % (± 0.9 %) for elemental nickel.

3.2 Higher nickel fluorides NiF_4 and NiF_3

3.2.1 Sample productions

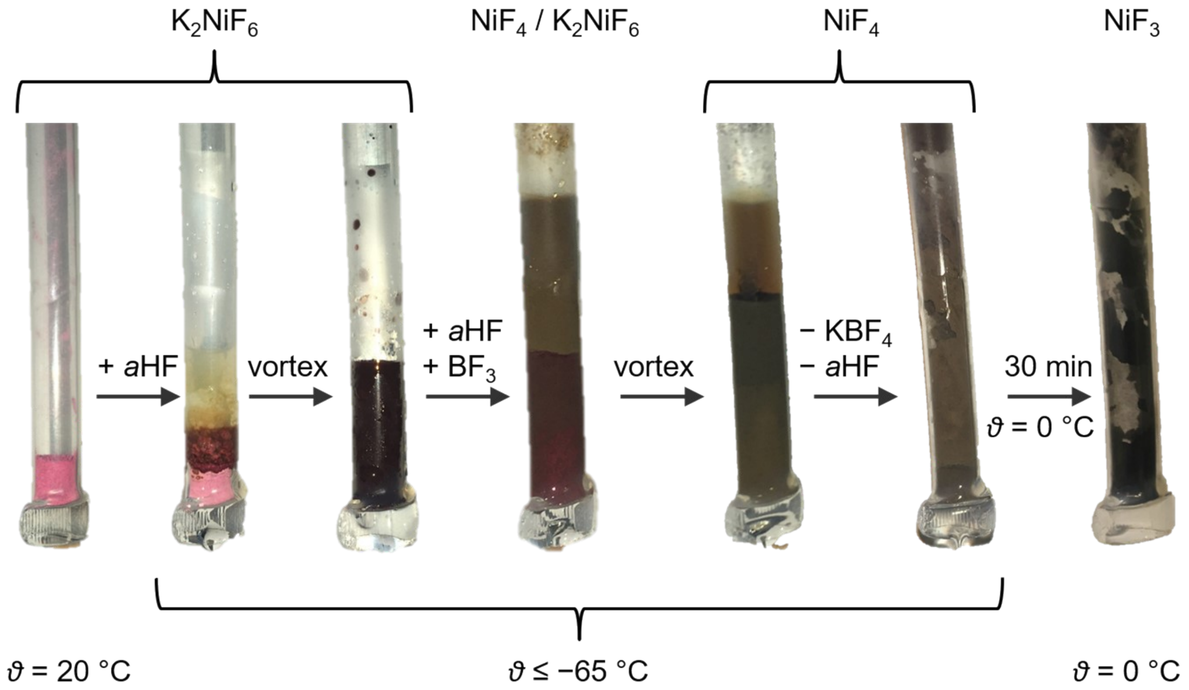

Figure S33: Preparation of an NiF_4 sample and its decomposition to NiF_3 (Section **1.3**) for XANES measurements in a λ-shaped reactor made from FEP tubing with only the main arm shown.

3.2.2 XANES data

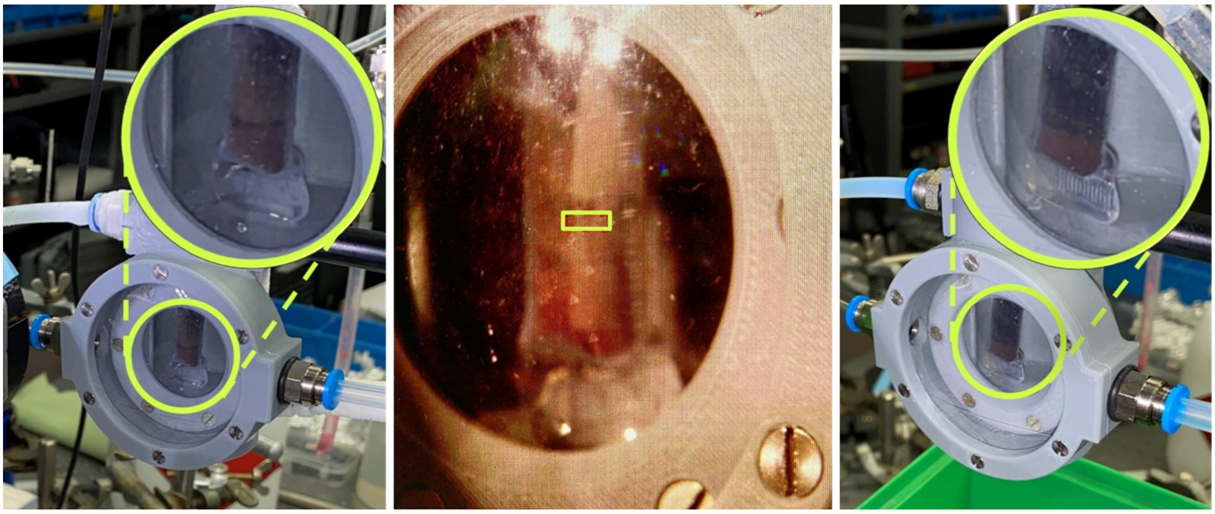

Figure S34: NiF_4 (left, $\vartheta < -60$ °C) and NiF_3 (right, $\vartheta = 0$ °C) samples in a cooled FEP tube and the beam spot (center, yellow highlighted box) for XANES reference measurements.

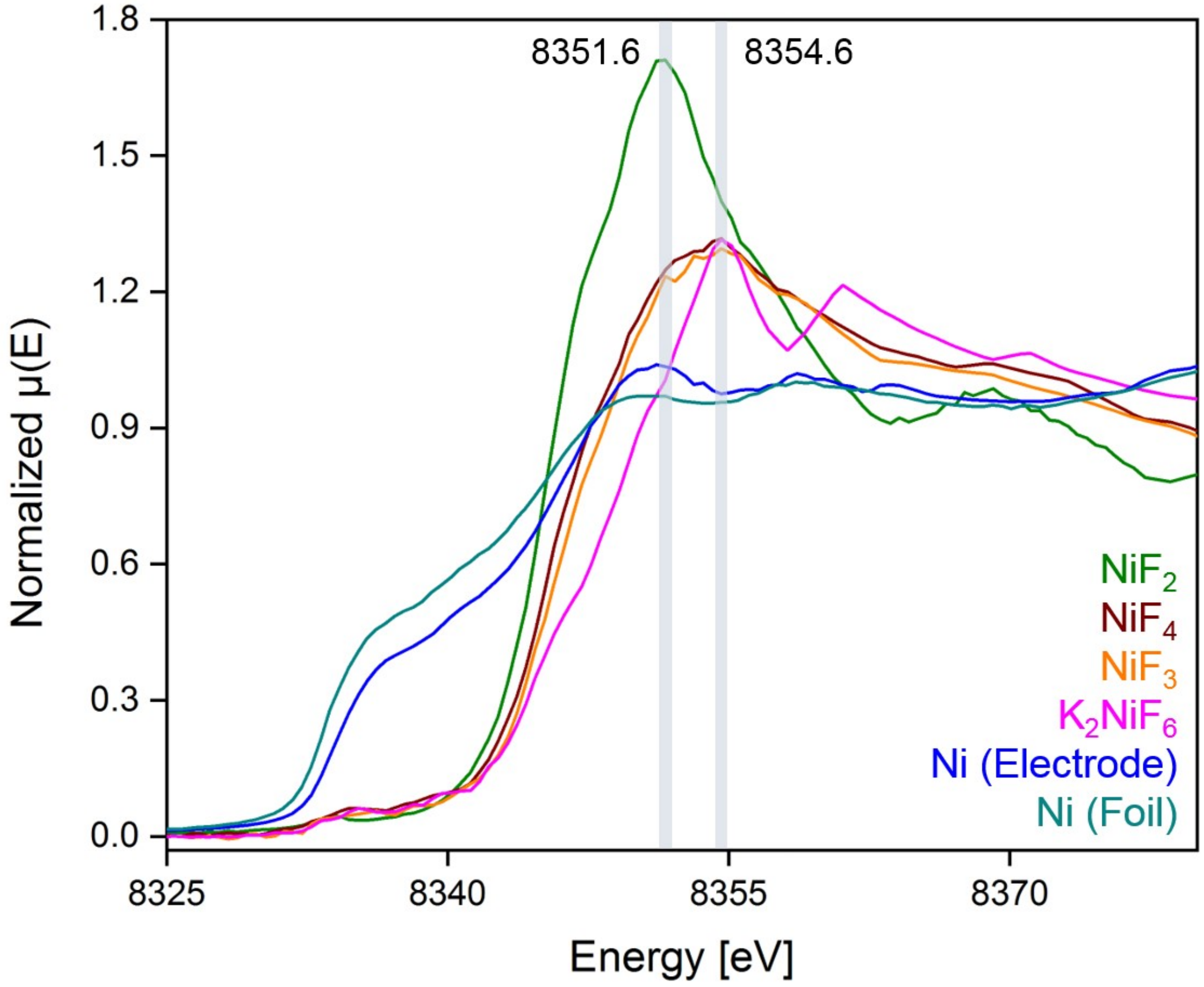

Figure S35: Ni K-edge XANES spectra of the standards NiF_2 (green), NiF_4 (brown), NiF_3 (orange), K_2NiF_6 (pink), Ni (bulk anode exposed to HF, blue) and Ni (foil, transmission measurement, turquoise) in the range from 8325 to 8380 eV at a spectral resolution of 0.5 eV. The maxima of absorption were highlighted. A quantitative conversion of K_2NiF_6 to the binary reference materials NiF_4 and NiF_3 is indicated by the different post-edge features.

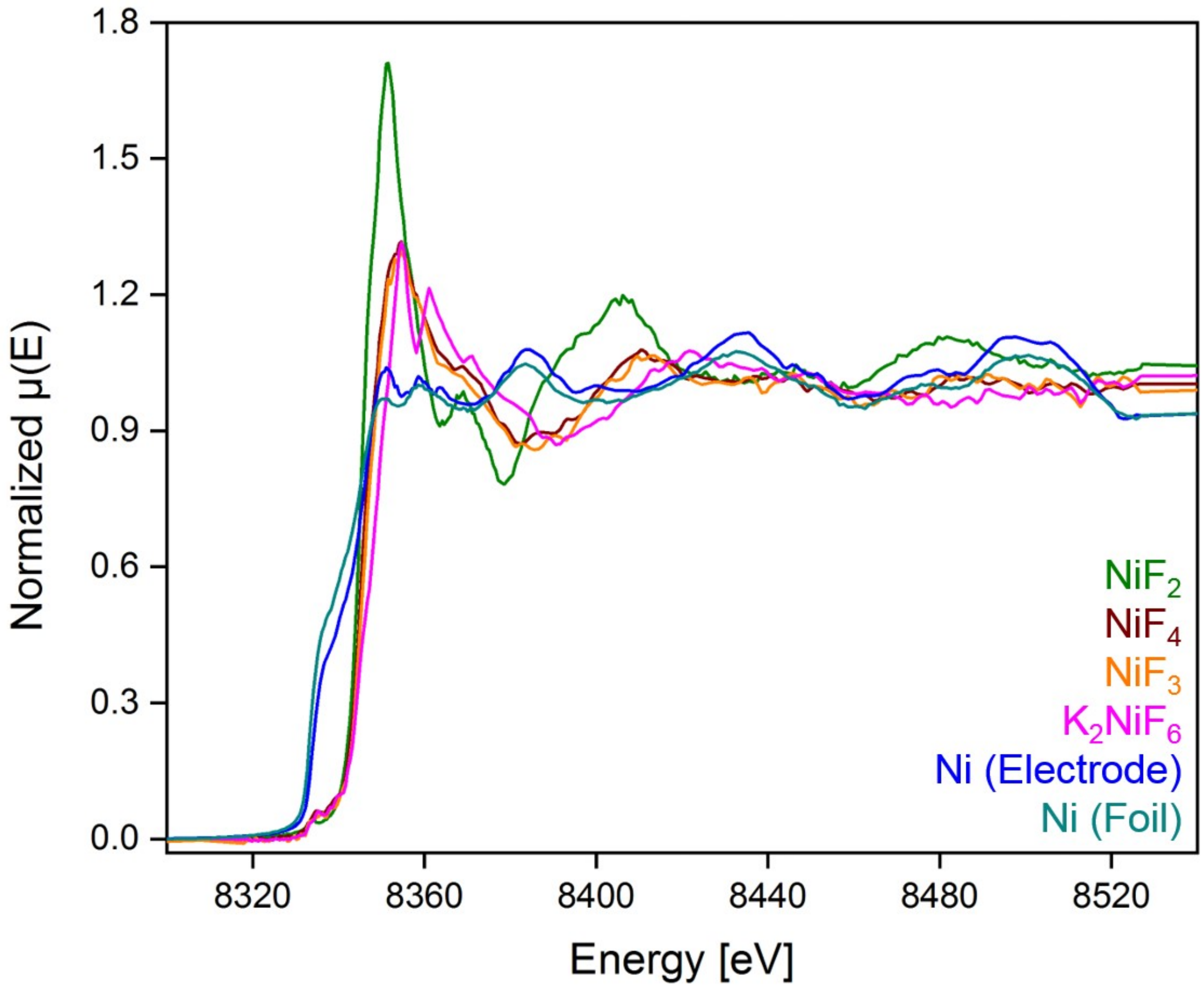

Figure S36: Ni K-edge XANES spectra of the standards NiF_2 (green), NiF_4 (brown), NiF_3 (orange), K_2NiF_6 (pink), Ni (bulk anode exposed to HF, blue) and Ni (foil, transmission measurement, turquoise) in the range from 8300 to 8540 eV at a spectral resolution of 0.5 eV.

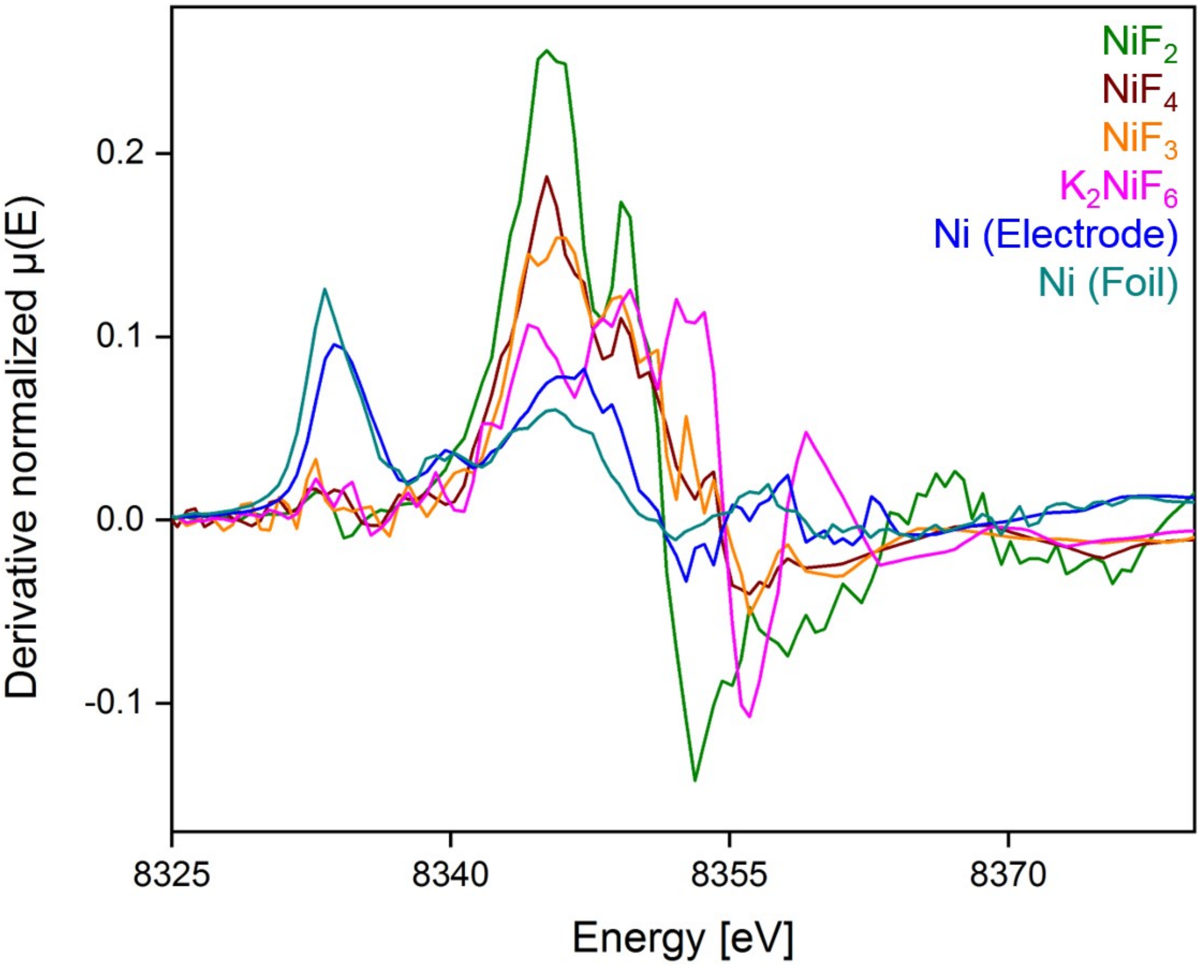

Figure S37: First derivative of Ni K-edge XANES spectra of the standards NiF_2 (green), NiF_4 (brown), NiF_3 (orange), K_2NiF_6 (pink), Ni (bulk anode exposed to HF, blue) and Ni (foil, transmission measurement, turquoise) in the range from 8325 to 8380 eV at a spectral resolution of 0.5 eV.

3.2.3 EXAFS data

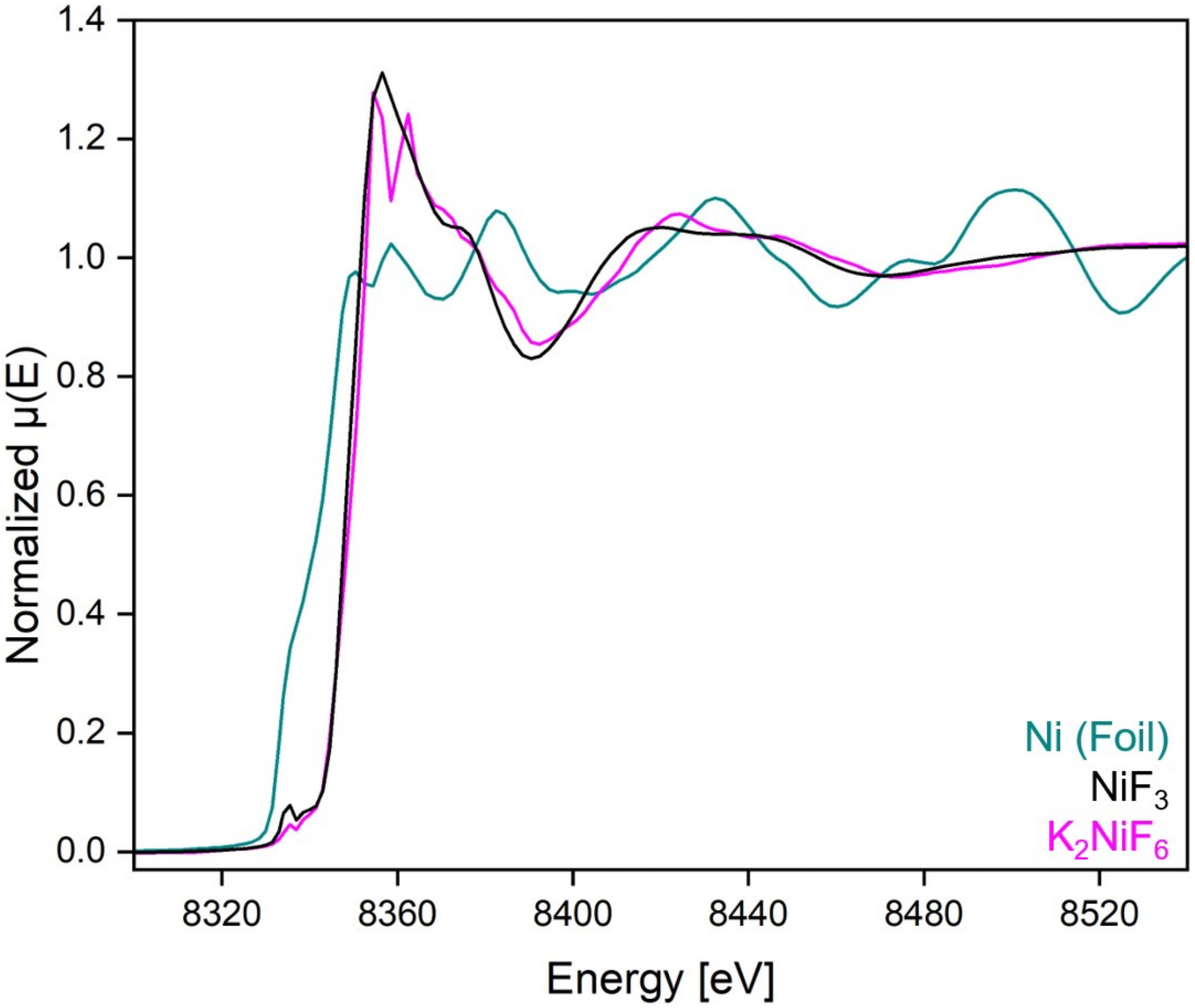

Figure S38: Ni K-edge XANES spectra acquired in transmission of Ni (in turquoise), NiF_3 (in black), and K_2NiF_6 (in magenta) at a spectral resolution of 0.5 eV.

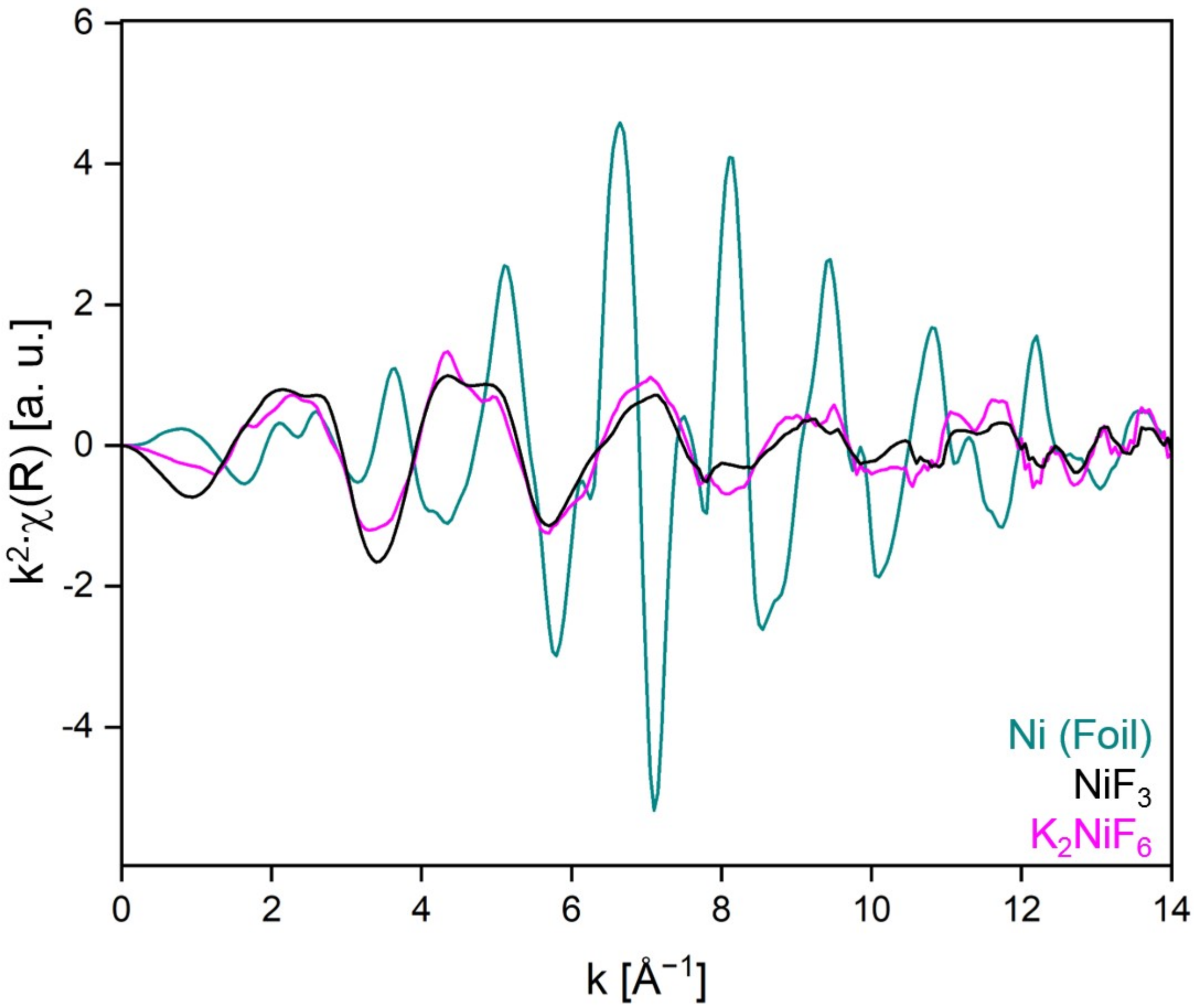

Figure S39: Visualization of the k^2-weighted EXAFS oscillations (see also Fig. S38) in k-space of Ni (in turquoise), NiF_3 (in black), and K_2NiF_6 (in magenta).

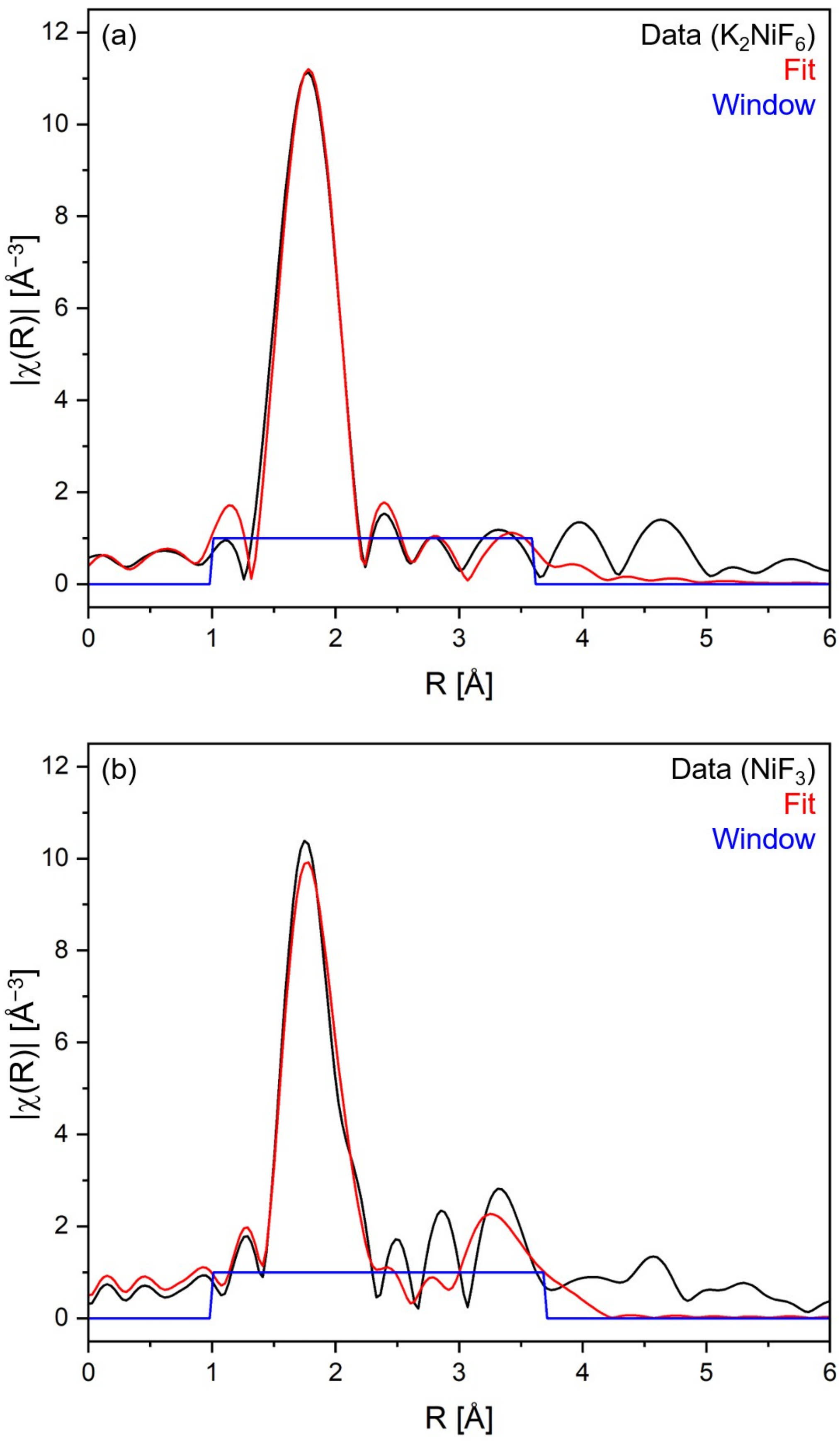

Figure S40: EXAFS oscillations (see also Fig. S38) as a function of the distance *R* obtained by Fourier transformation of a) K_2NiF_6 (in black) and the fitted data (in red, structure taken from reference [4]) in the window from 1.0 to 3.6 Å (in blue), and of b) NiF_3 (in black) and the fitted data (in red, structure taken from reference [5]) in the window from 1.0 to 3.7 Å (in blue).

Table S2: Ni K-edge EXAFS data of NiF_3 ($LiSbF_6$ type) and K_2NiF_6 (K_2PtCl_6 type) fitted to the neutron diffraction data taken from the literature.[4,5] Data in the table show the results from the EXAFS fits, where the guess parameters were calculated. Colum as 'd' is the bond distances from the model, 'd_{fit}' is the calculated bond distance after fitted to the model, 'sigma^2' is the calculated debye-waller factor, and delR is the difference between d and d_{fit}.

**Fit to NiF3 and NiF3_LiSbF6**							
**k-range**	**2 – 12 Å^{-1}**						
**R-range**	**1 – 3.7 Å**						
**Independent points**	**23.13**						
**Number of variables**	**15**						
Red. χ^2	349						
R-factor	0.014						
**guess parameters**	**amp**	**0.74 ± 0.05**					
	**enot**	**-1.2 ± 0.1**					
	**delr_F1.1**	**-0.075 ± 0.008**					
	**ss_F1.1**	**0.0013 ± 0.0005**					
	**delr_F1.2**	**-0.05 ± 0.01**					
	**ss_F1.2**	**0.0025 ± 0.0004**					
	**delr_Ni**	**0.063 ± 0.004**					
	**ss_Ni**	**0.017 ± 0.007**					
**Sample**	**C.S.**[a)]	**Path [Model]**	**C.N.**[b)]	***d* [Å]**	**d_{lit}[Å]**	**sigma^2 [Å^2]**	**delR**
NiF_3	1	F1.1 [Ni^{IV}–F; NiF3_LiSbF6]	3	1.75	1.82	0.00126	−0.075
	1	F1.1 [Ni^{II}–F; NiF3]	3	1.88	1.95	0.00126	−0.075
	2	F1.2 [Ni^{IV}–F; NiF3_LiSbF6]	3	3.45	3.51	0.00247	−0.053
	2	F1.2 [Ni^{II}–F; NiF3]	3	3.35	3.40	0.00247	−0.053
	3	Ni2.1 [Ni–Ni; NiF3;LiSbF6]	6	3.59	3.53	0.01688	0.063
**Fit to K2NiF6_K2PtCl6**							
**k-range**	2 – 10 **Å^{-1}**						
**R-range**	1 – 3.7 **Å**						
**Independent points**	13						
**Number of variables**	6						
Red. χ^2	1369.4						
R-factor	0.015						
**guess parameters**	**amp**	0.71 ± 0.04					
	**enot**	-3.5 ± 0.2					
	**delr_F**	0.021 ± 0 009					
	ss_F	0.0009 ± 0.0003					
	delr_K	0.12 ± 0.05					
	ss_K	0.021 ± 0.009					
**Sample**	**C.S.**[a)]	**Path [Model]**	**C.N.**[b)]	***d* [Å]**	**d_{lit}[Å]**	**sigma^2 [Å^2]**	**delR**
K_2NiF_6	1	F1.1 [Ni–F]	6	1.80	1.78	0.00088	0.021
	2	K1.1 [Ni–K]	8	3.63	3.51	0.02136	0.120

a) C.S. = coordination sphere; b) C.N. = coordination number

3.3 Electrochemical characteristics of the in-situ XAS cell

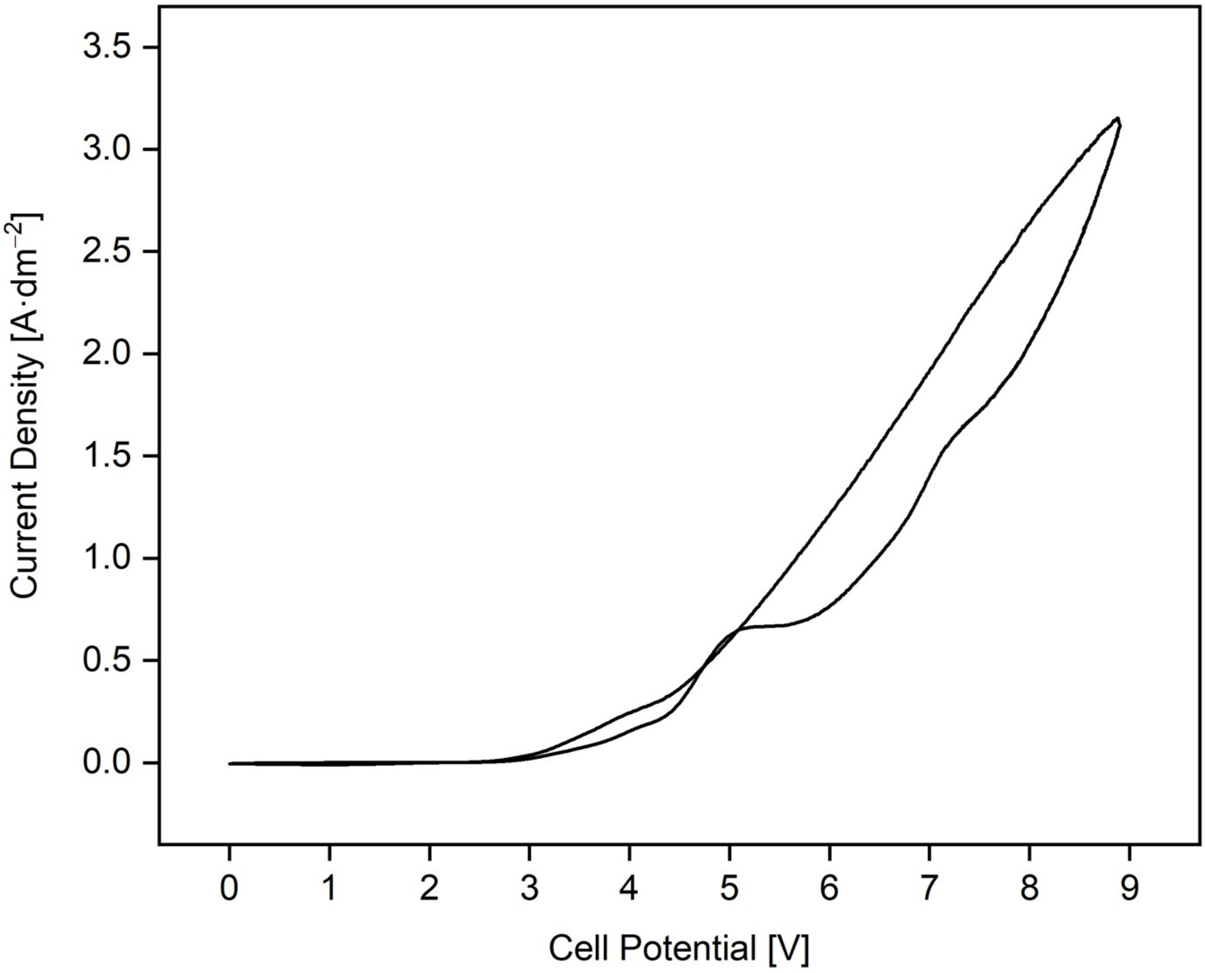

Figure S41: Cyclic voltammogram of nickel anode and cathode in liquid *a*HF in the 2-electrode in-situ XAS cell in the potential window from 0 to +9.0 V. Only one distinct oxidation feature was observed at a cell potential of +5.25 V. The electrode arrangement in this setup is seen to cause an increase of the oxidation potential by +0.7 V compared to earlier findings[10], as well as the crossing[11] of the backward and the forward scan.

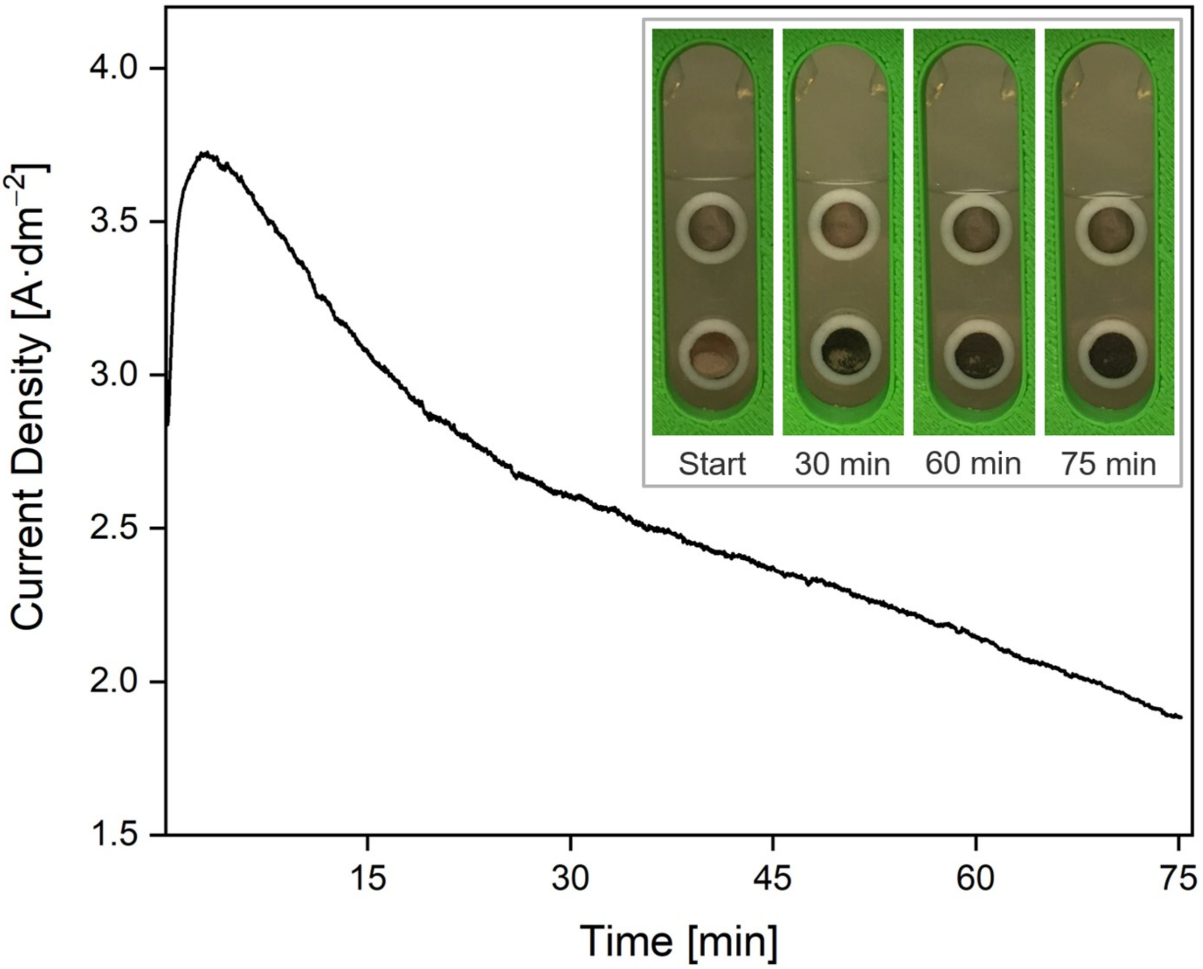

Figure S42: Chronoamperogram of a preliminary test for the generation of a black film on the nickel anode at a cell potential of +8.0 V in the in-situ XAS cell setup and corresponding photos of the nickel anode (bottom) and cathode (top) in liquid *a*HF. The complete covering with a black film on the anode was achieved after 75 min. The greyish shadow on the cathode and slightly brownish shadow on the anode in the beginning stem from previous experiments with a reverse polarization of the electrodes.

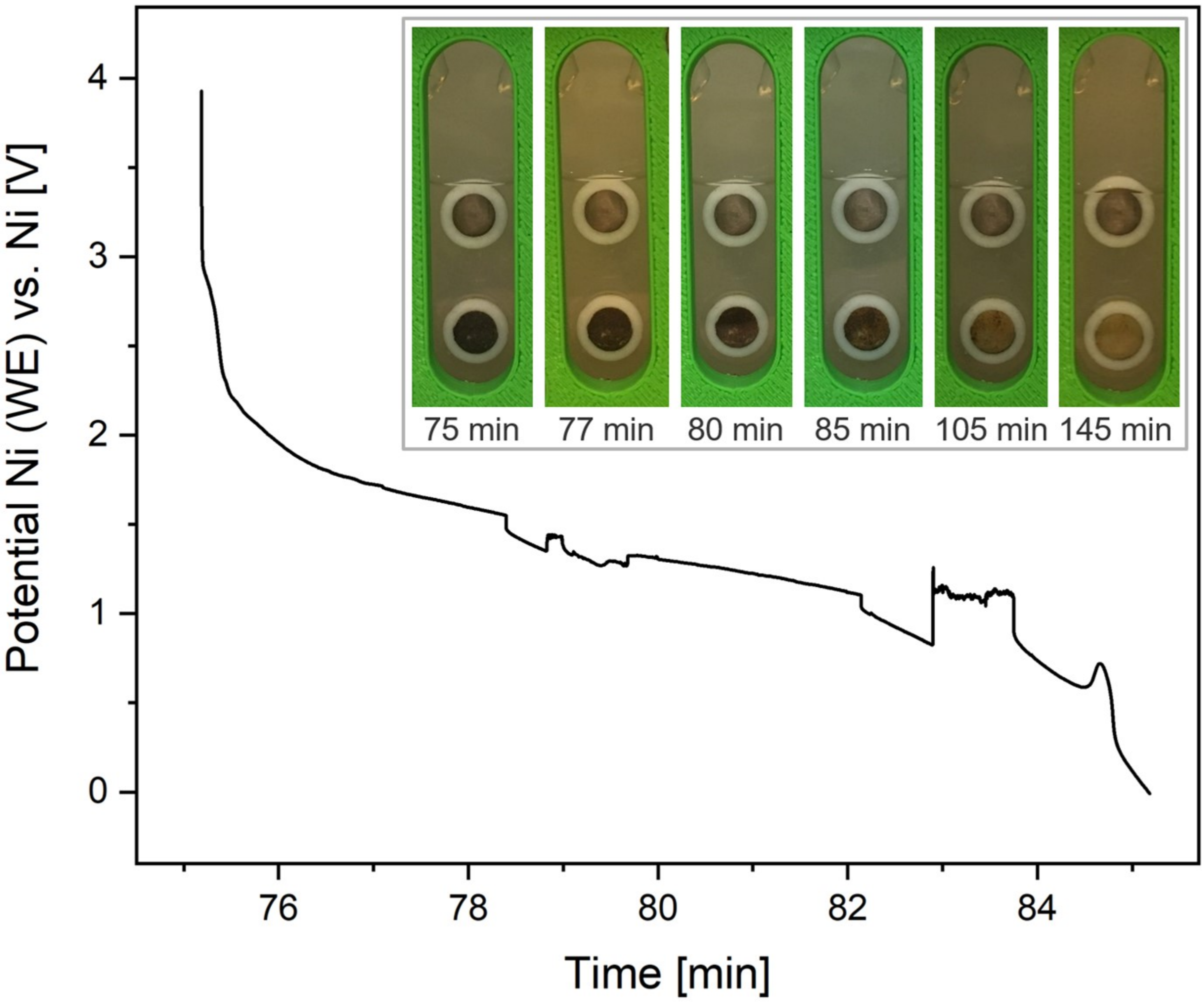

Figure S43: OCV scan until 10 min after the generation of a black film on the nickel anode at a cell potential of +8.0 V in liquid *a*HF during a preliminary test for the in-situ XAS cell setup and corresponding photos of the nickel anode (bottom) and cathode (top). The time scale refers to the absolute run time of the experiment, including the conditioning phase (Fig. S42). The black color of the generated anodic film bleached during 10 min at open circuit conditions, leaving a brown and later colorless decomposed film on the anode. The greyish shadow on the cathode stems from previous experiments with a reverse polarization of the electrodes (see also Fig. S42).

4 XAS cells – construction and functional principles

4.1 In-situ XAS cell

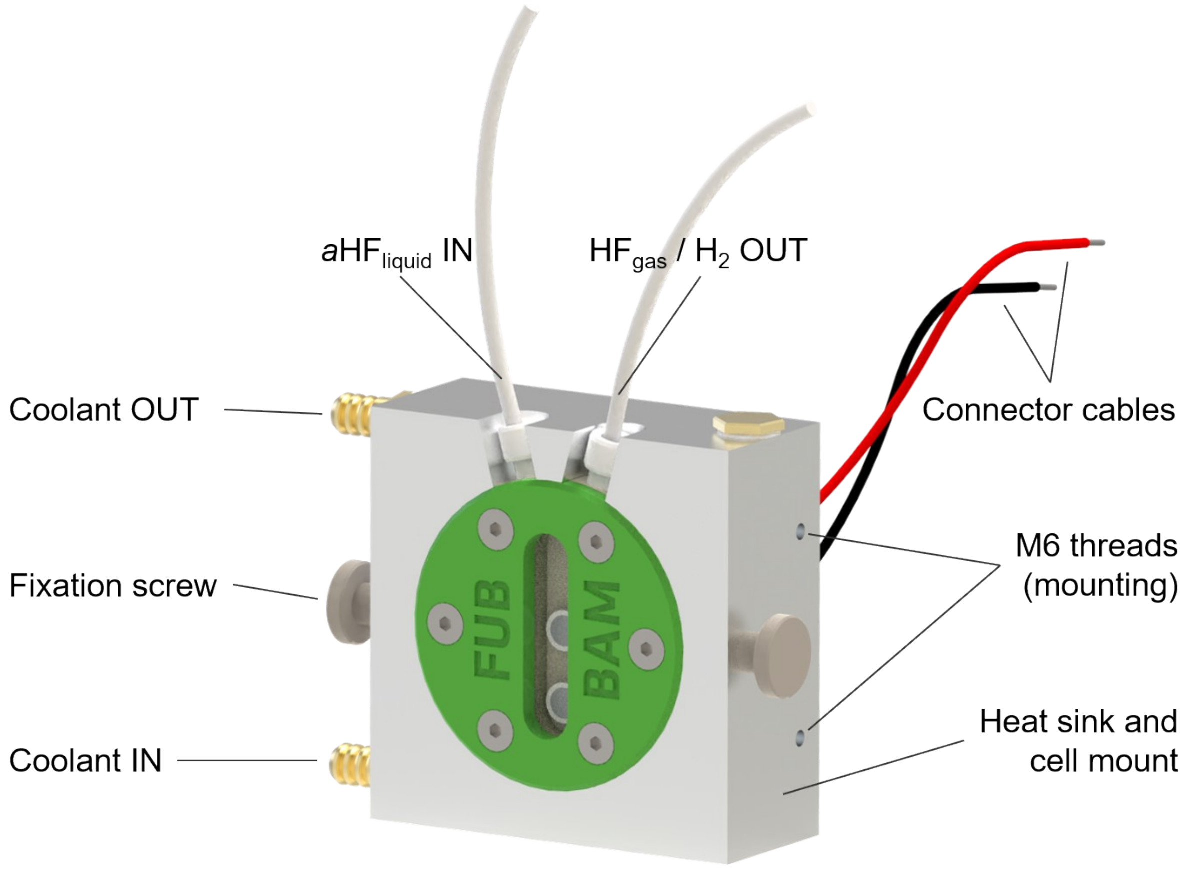

Figure S44: Schematic of the in-situ XAS cell with aluminum heat sink. The fixation screws are used for fixing the electrochemical cell within the aluminum body and the M6 threads are used for connecting the whole setup to a stable mount.

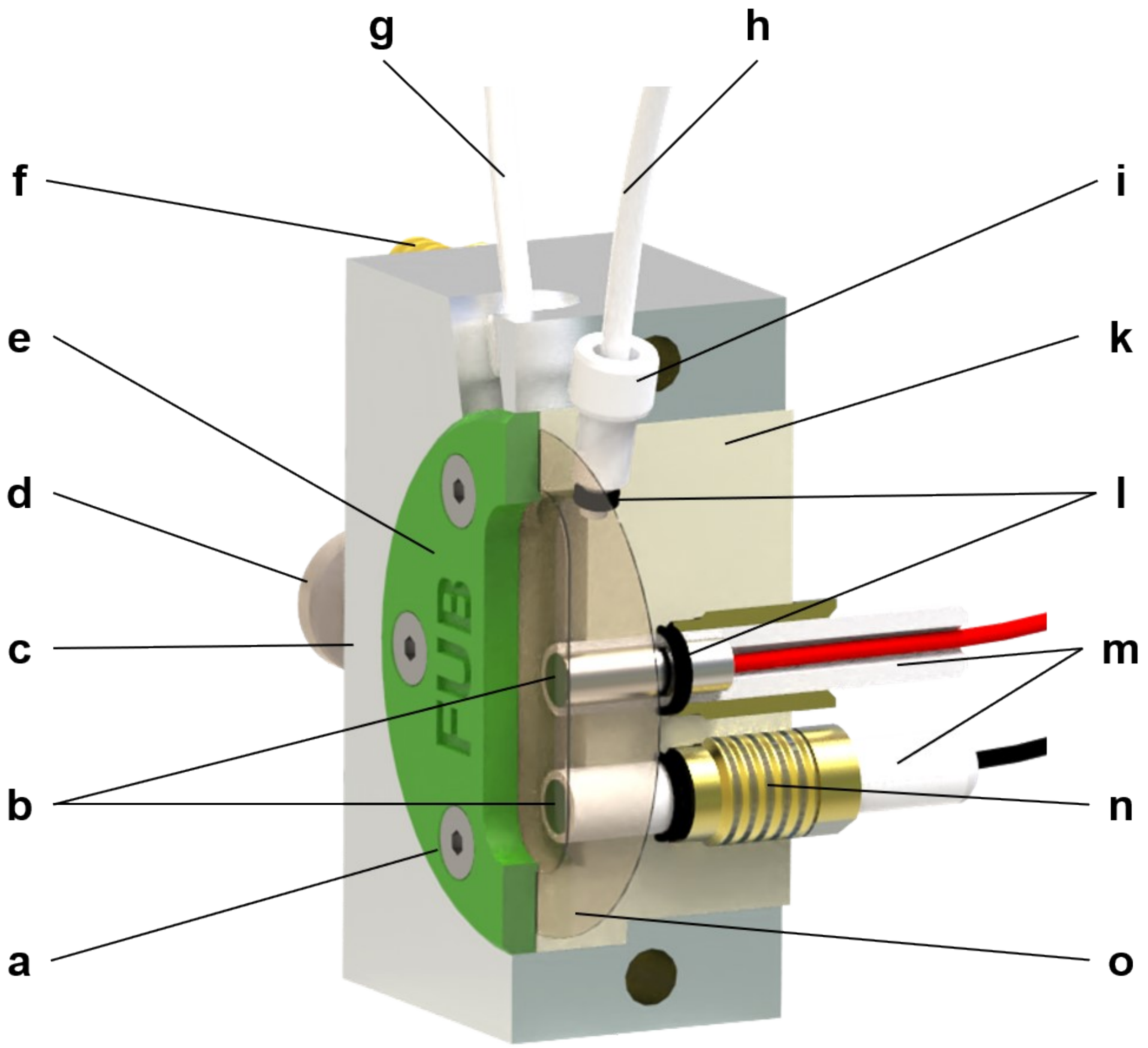

Figure S45: Cutaway drawing of the in-situ XAS cell with its components and the materials thereof (in parentheses): **a**) countersunk bolt DIN 7991 M4 (stainless steel), **b**) planar electrodes (nickel, 99.99 %), **c**) heat sink and mount (aluminum), **d**) knurled-head screw (PVC) for fixation of the cell, **e**) cell cap/pressure piece (3D-printed ABS), **f**) hose connector (brass), **g**) inlet for liquid *a*HF (PFA), **h**) outlet for exhaust gases H_2 and (gaseous) HF (PFA), **i**) compression fitting (PTFE), **k**) cell body (PCTFE), **l**) O-rings (FKM), **m**) coating of the planar electrodes (PTFE), **n**) compression fitting (brass), and **o**) window (FEP, 50 µm). For increased safety, two O-rings were used for sealing electrodes as well as *a*HF inlet and exhaust gas tubings.

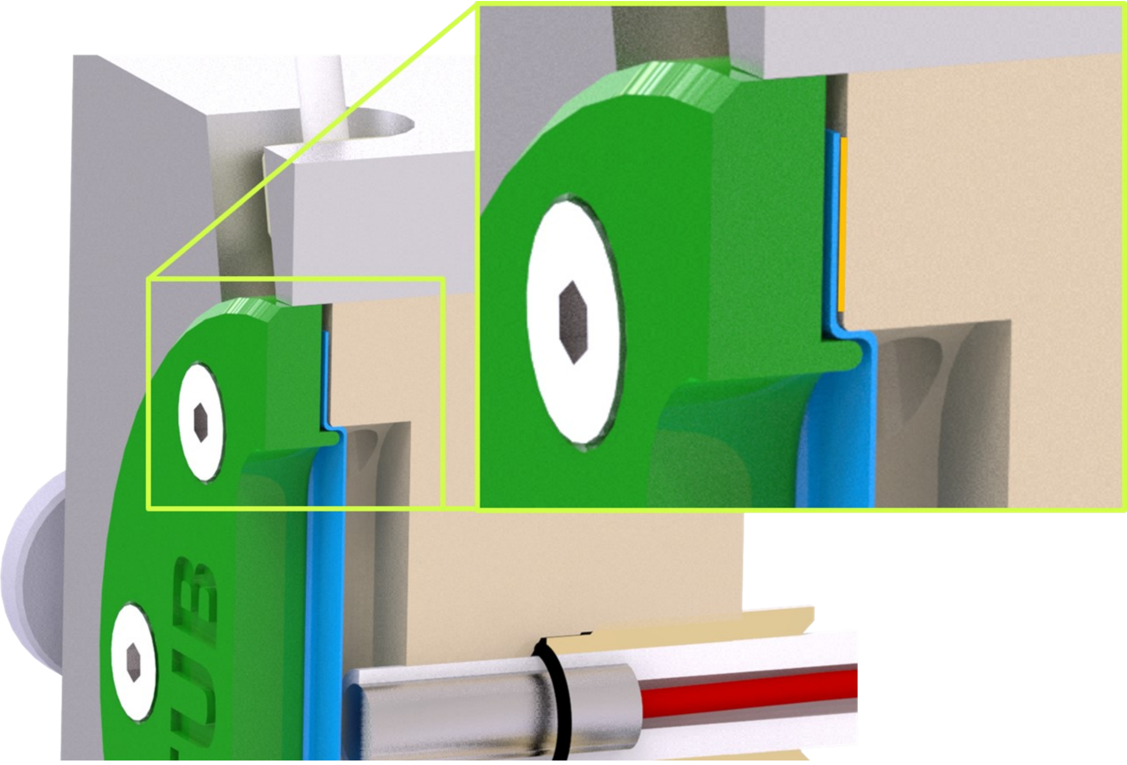

Figure S46: Sealing principle of the in-situ XAS cell with the FEP foil highlighted blue. By adjusting the 3D-printed green pressure piece to the oblong hole of the liquid *α*HF-container of the cell, a tight contact between cell body and FEP foil was achieved and maintained by six M4 screws with corresponding washers and nuts on the backside of the cell (see also Fig. S47). Due to the low surface tension of *α*HF,[12] halocarbon wax (25-20M Grease, *Halocarbon*®) was used as an additional sealant on the surrounding contact plane between the FEP foil and the PCTFE cell body (highlighted orange).

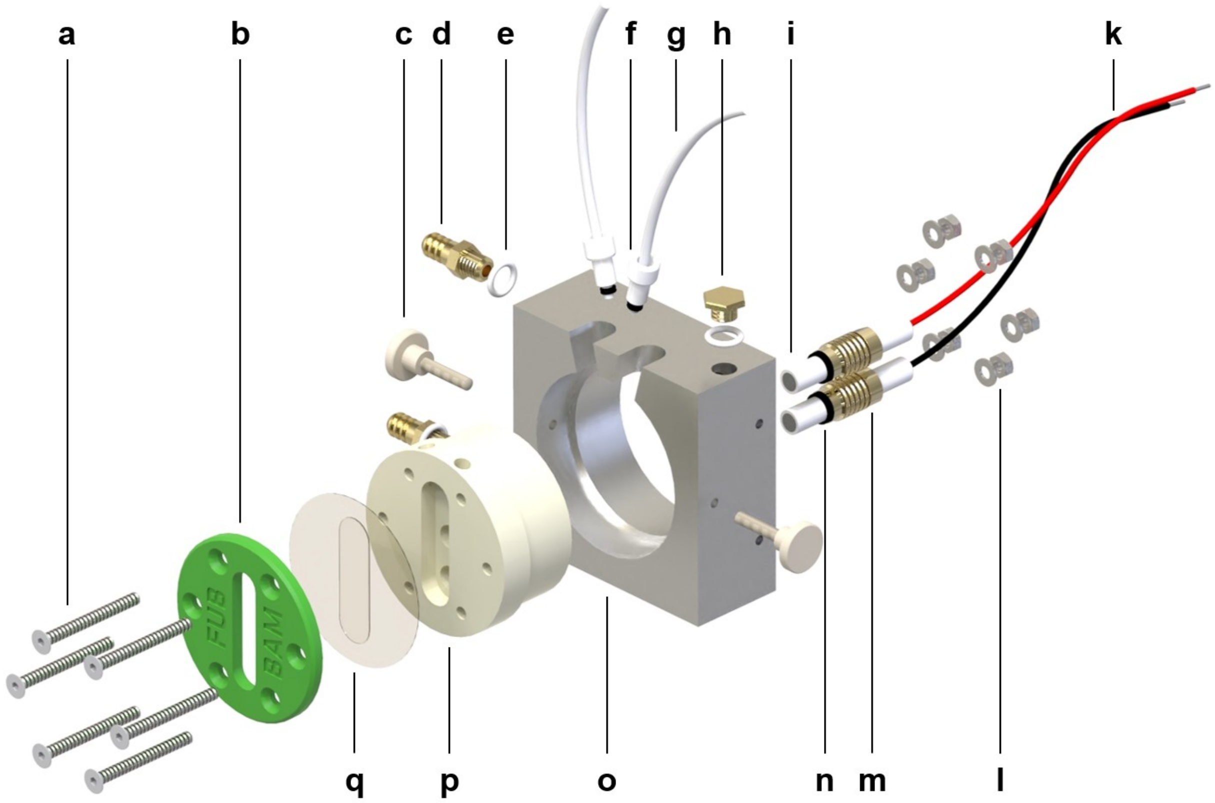

Figure S47: Exploded assembly drawing of the in-situ XAS cell. Analogous parts have been singly highlighted for clarity: **a**) countersunk bolt DIN 7991 M4 (stainless steel), **b**) cell cap/pressure piece (3D-printed ABS), **c**) knurled-head cell fixation screw (PVC), **d**) hose connector (brass), **e**) flange seal (PTFE), **f**) compression fitting (PTFE), **g**) tubing for *a*HF inlet and exhaust gases (PFA), **h**) seal plug (brass), **i**) planar nickel electrodes (PTFE coated Ni (99.99 %)), **k**) connector cables, **l**) M4 nuts and washers (stainless steel), **m**) compression fitting (brass), **n**) O-rings (FKM), **o**) heat sink and mount (aluminum), **p**) cell body (PCTFE), and **q**) window (FEP, 50 µm). The FEP foil is depicted with the fold edge resulting from mounting the cell cap/pressure piece. For increased safety, two O-rings were used for sealing electrodes as well as *a*HF inlet and exhaust gas tubings.

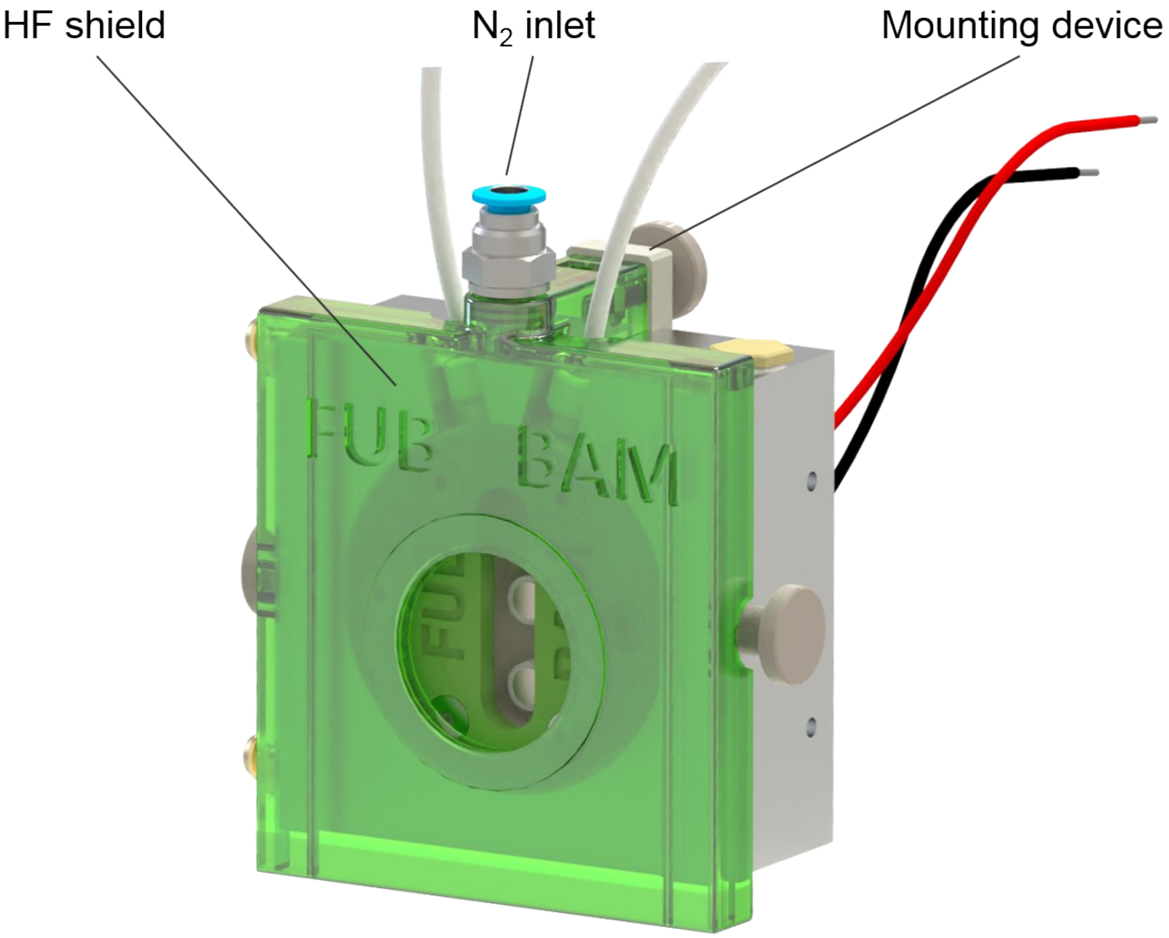

Figure S48: Scheme of the in-situ XAS cell assembled with an additional HF shield. Only parts in addition to the fundamental parts of the electrochemical cell (Fig. S45-S47) are highlighted. The HF shield allowed for the application of a slight, constant stream of nitrogen (see also Fig. S49) in order to transport traces of HF exiting the window (HF diffuses through FEP)[13] to a deactivating KOH/ice-mixture (separate container not shown) below the cell.

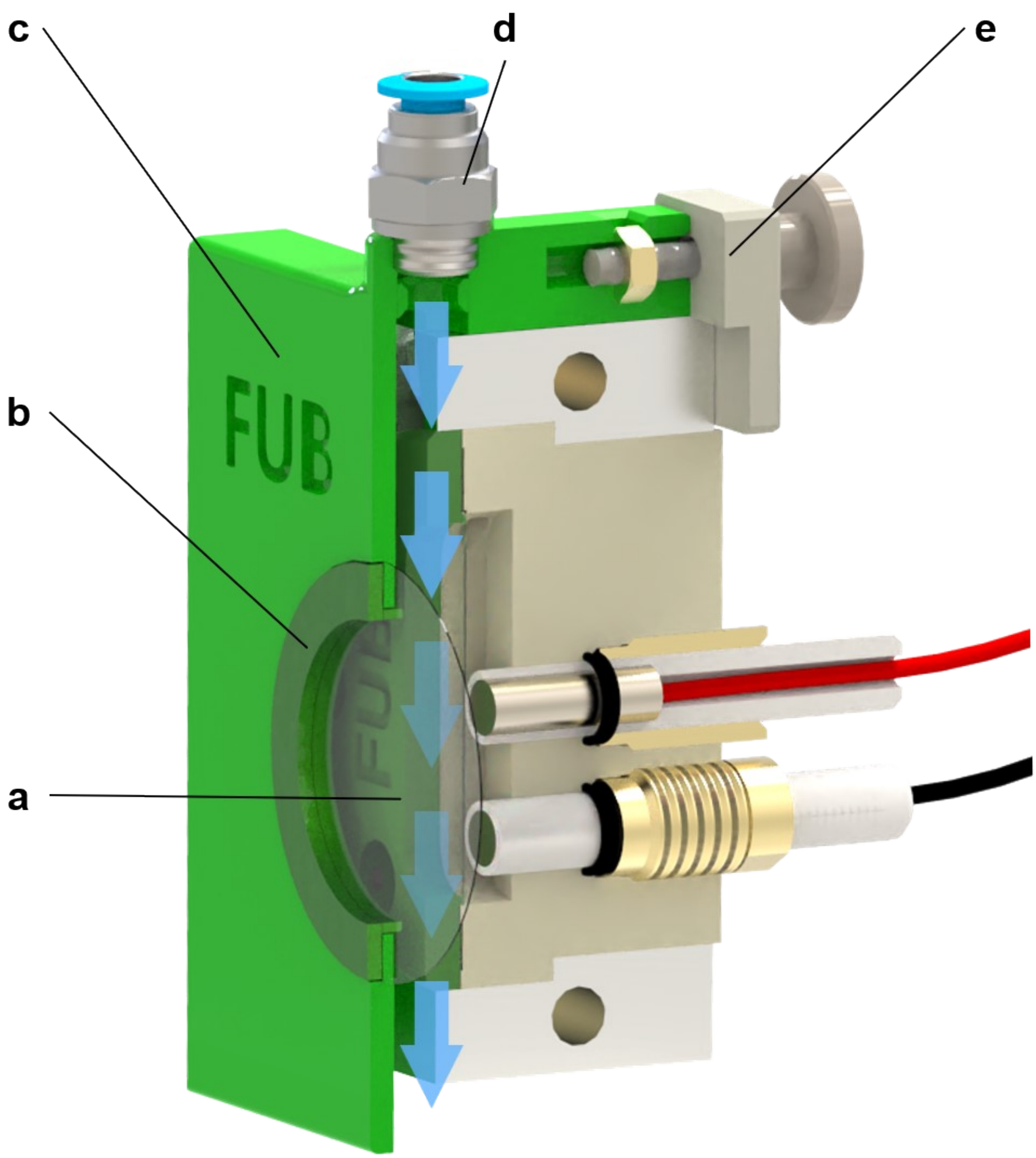

Figure S49: Functional principle of the in-situ XAS cell/HF shield assembly. Only parts in addition to the fundamental parts of the electrochemical cell (Fig. S45-S47) are highlighted: **a**) window (FEP, 25 µm), **b**) foil fixation ring (3D-printed ABS), **c**) HF-shield with applicable N_2 stream (3D-printed ABS), **d**) push-in fitting (Festo QS-G1/8-6 (186096)), and **e**) mounting device consisting of screw (M3, stainless steel), knurled screw cap (3D-printed ABS) and nut (M3, stainless steel). The window **a** was glued to the shield **c** with halocarbon wax (25-20M Grease, *Halocarbon®*) and fixed with the fixation ring **b**. The blue arrows depict the direction of the N_2 stream.

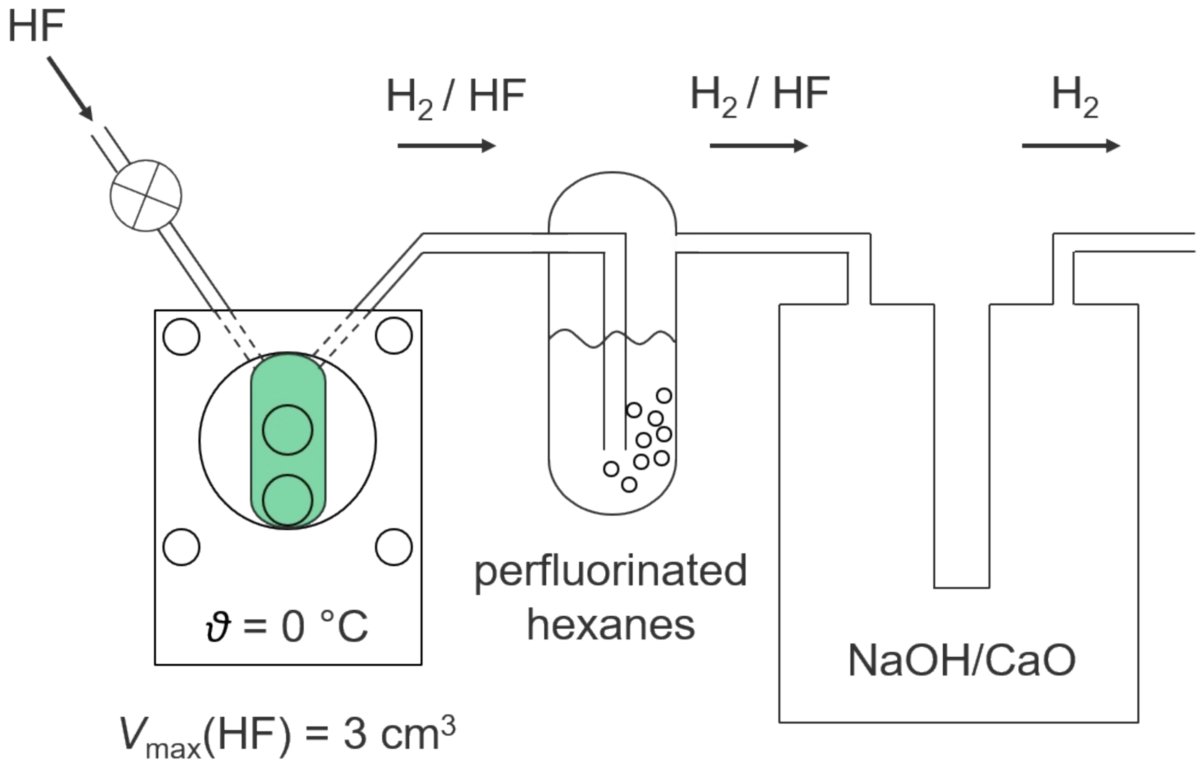

Figure S50: Schematic of an in-situ XAS cell with a mobile HF deactivation. The green highlighted area depicts the *a*HF container of the electrochemical cell with a maximum filling volume of 3 cm^3. Gases (H_2, HF_{gas}) exiting the cell pass a bubbler filled with perfluorinated hexanes (98 %, 85 % n-isomer, *ABCR*), maintaining a closed system. Gaseous HF is then deactivated in a U-tube filled with soda lime (NaOH/CaO).

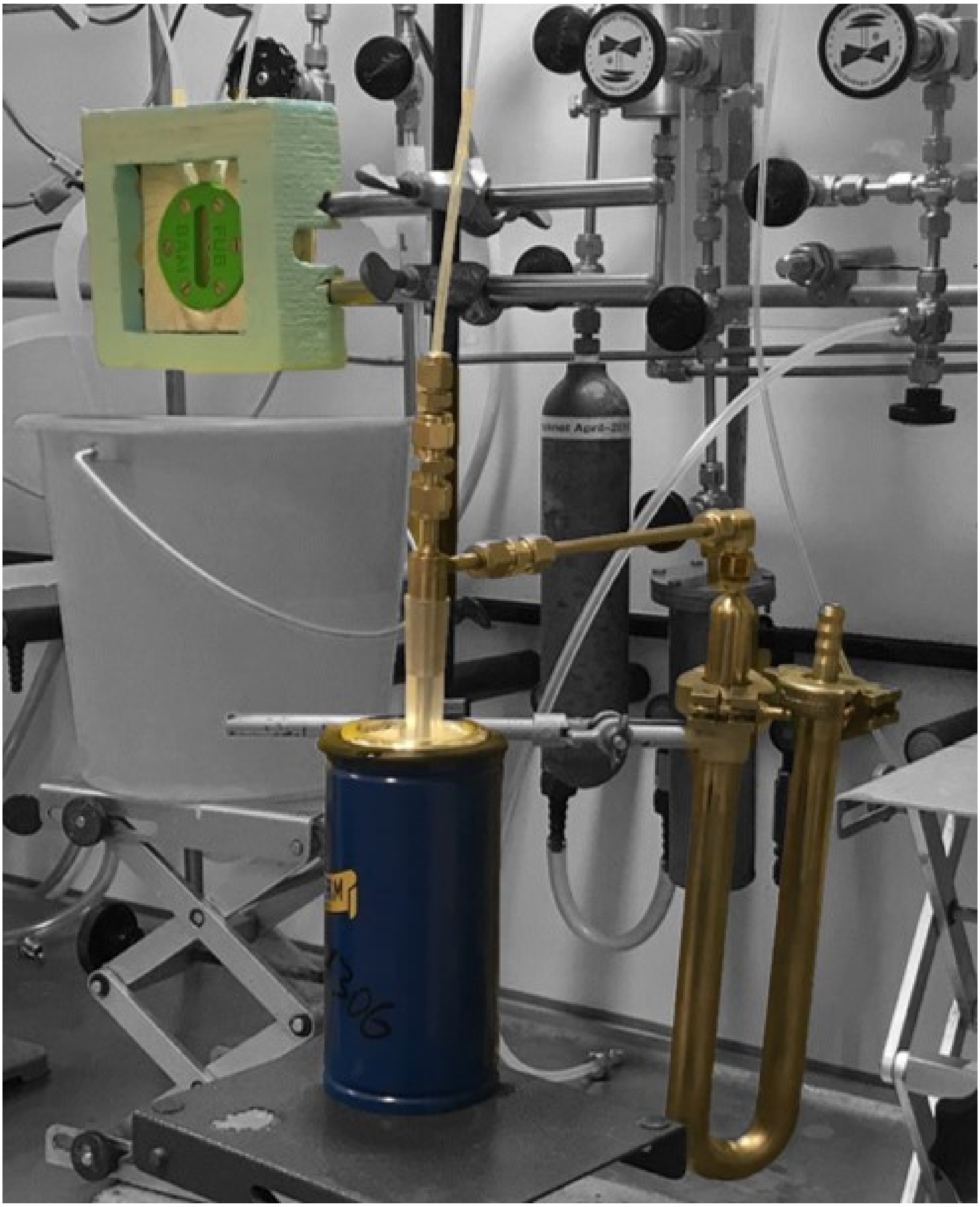

Figure S51: Setup of the in-situ XAS cell with a mobile HF deactivation (brass-colored highlights; see also schematic in Fig. S50). The authors recommend flame sealing of the HF inlet after filling of the cell and cooling the bubbler containing the perfluorinated hexanes (98 %, 85 % n-isomer, *ABCR*) at ϑ = 0 °C. All parts in contact with HF were made from fluoroplastics or stainless steel. The mobile HF deactivation was used for the low temperature XAS cell for reference measurements of NiF_4 and NiF_3 as well (see Section **4.2**).

4.2 Low temperature XAS cell for reference measurements of NiF_4 and NiF_3

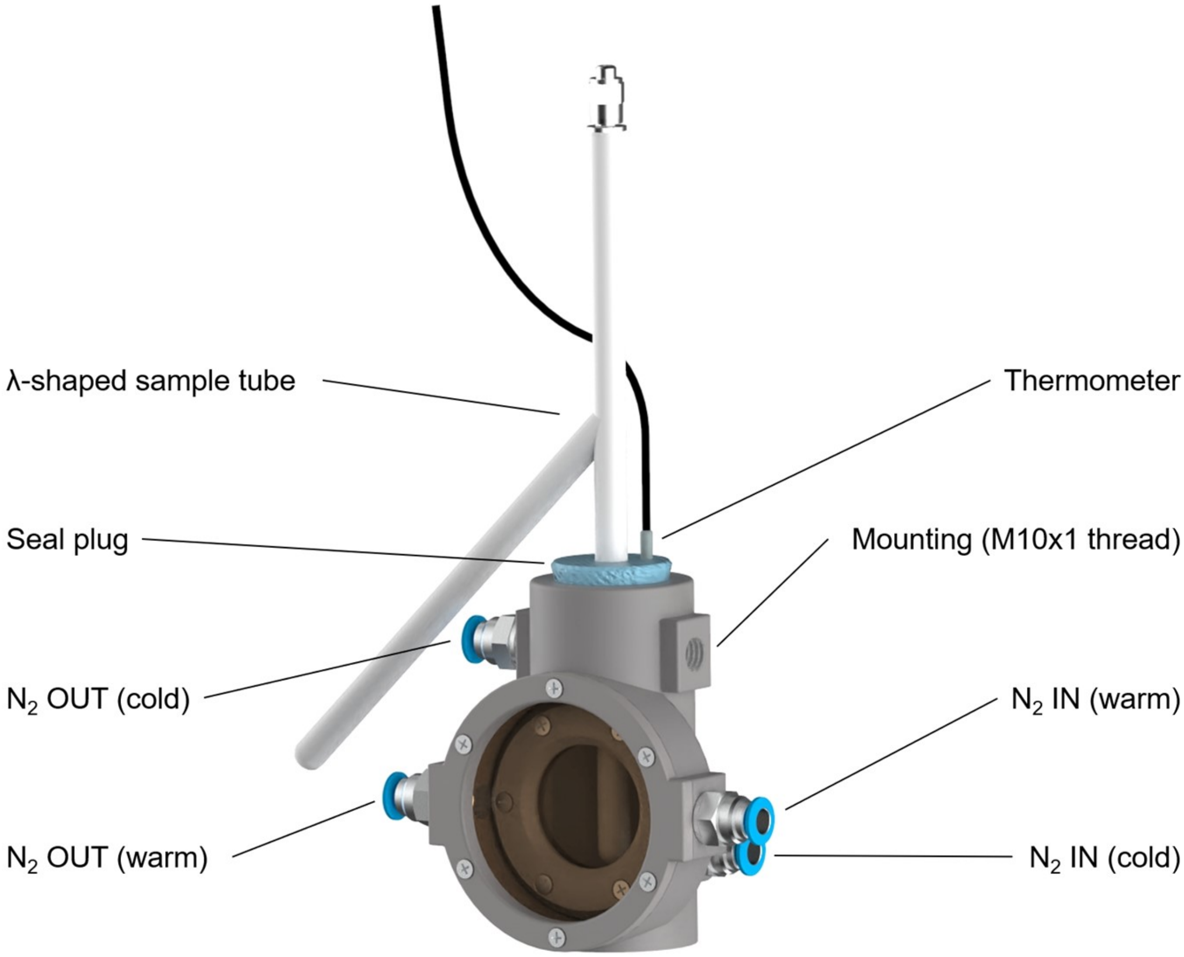

Figure S52: Functional principle of the low temperature XAS cell for reference measurements of NiF_4 and NiF_3 with a coolable inner sample container (cylindrical) and an outer, warm layer to prevent icing of the windows. A cold N_2 stream was obtained by passing a warm (r.t.) nitrogen stream through a Dewar vessel (set up like a bubbler) filled with liquid nitrogen. The desired temperature (e.g. $\vartheta < -60$ °C) was maintained by controlling the N_2 flow rate, leading to a lower temperature at a higher flow rate and vice versa. Due to the large window sizes, the cold and warm streams depend on each other, which necessitates independent flow rate controls. A second thermal sensor (not shown) was inserted into the warm N_2 outlet, and the temperature was maintained at temperatures not lower than +10 °C. (During preliminary tests of the cell, the condensation of water was observed at temperatures below +10 °C in the outer layer, depending on the humidity of the surrounding atmosphere).

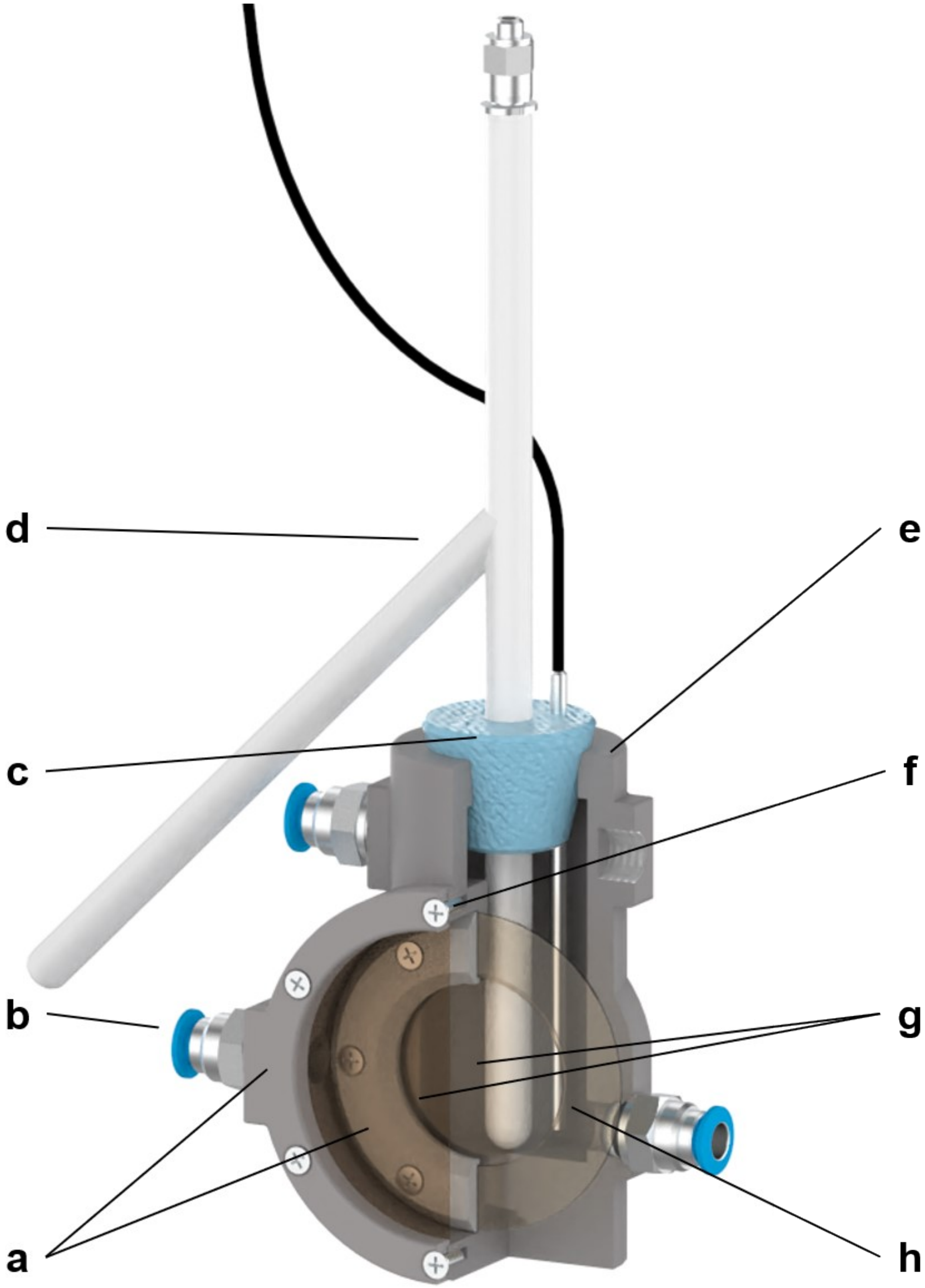

Figure S53: Cutaway drawing of the low temperature XAS cell for reference measurements of NiF_4 and NiF_3 with a coolable inner sample container (see also schematic in Fig. S52) with the components and their respective materials: **a**) pressure pieces (3D-printed ABS), **b**) push-in fitting (Festo QS-G1/8-6 (186096)), **c**) seal plug with drilled holes for thermal sensor and the sample tube (polystyrene), **d**) sample tube (FEP), **e**) cell body (3D-printed ABS), **f**) countersunk bolt ISO7046-1-M2, 5x6 (stainless steel), **g**) windows (FEP, 25 µm), and **h**) thermal sensor.

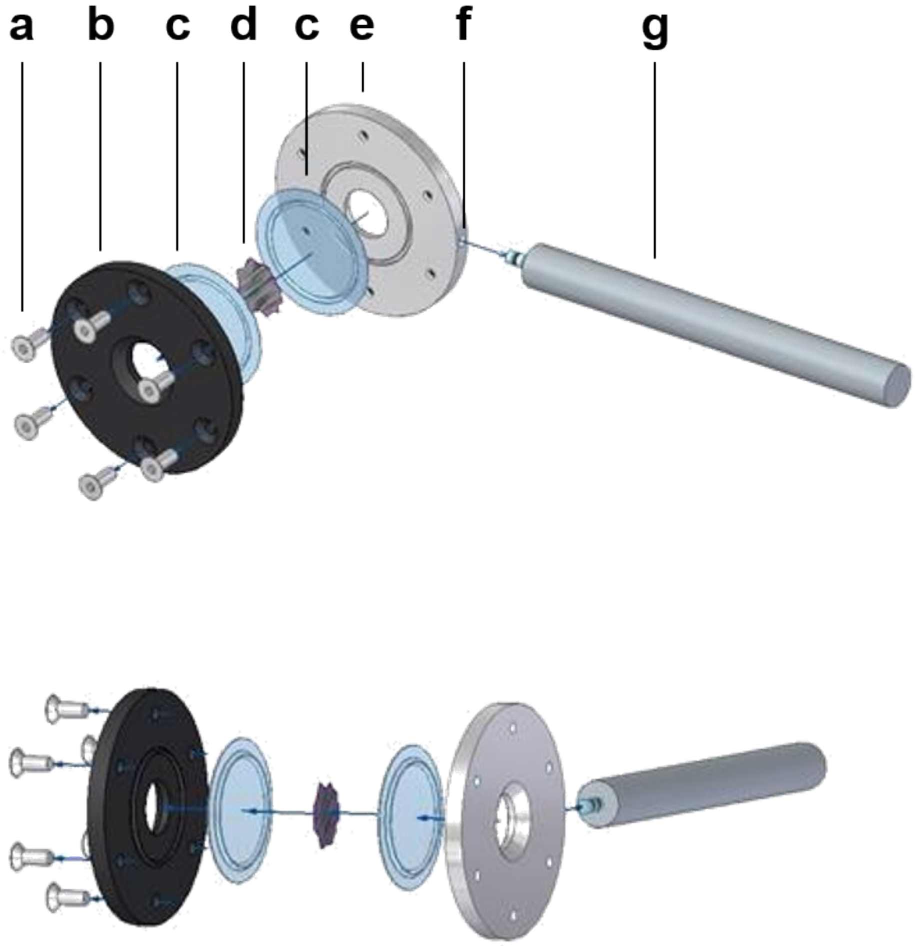

Figure S54: Exploded assembly drawings of the XAS cell for the measurement of spectra in transmission of NiF_3 and K_2NiF_6 at room temperature with the components and their respective materials: **a**) countersunk bolt DIN 7991 M3 (stainless steel), **b**) cell cap/pressure piece with a circumferential bead (3D-printed ABS), **c**) window (FEP, 100 µm), **d**) sample (NiF_3 or K_2NiF_6 powder), **e**) cell body/counter piece with a groove as a socket for **b** (aluminum), **f**) M4 thread, **g**) Mounting rod with an M4 thread (stainless steel). The samples were handled in a glovebox under argon atmosphere and portioned by the use of a self-made PTFE spatula. The cell was assembled within the glovebox.

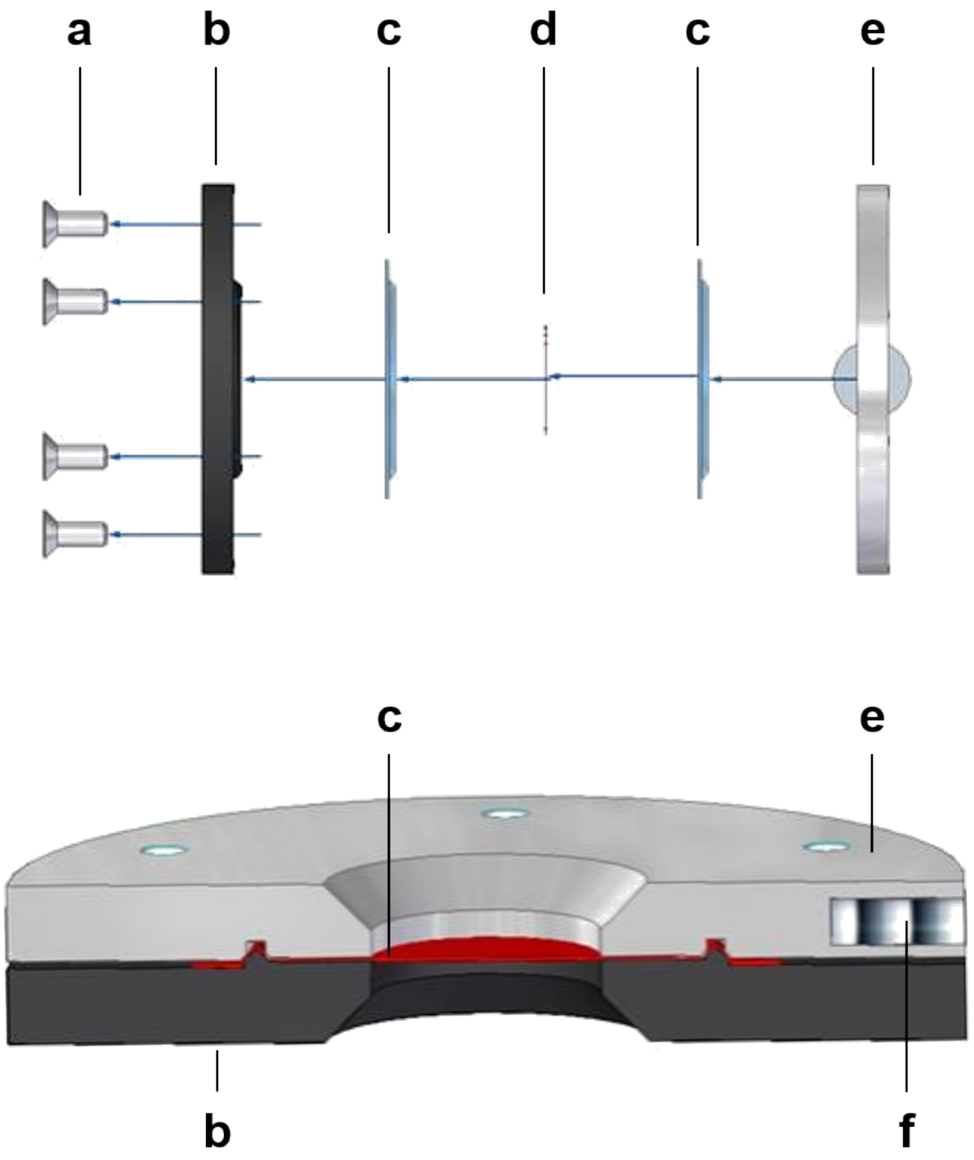

Figure S55: Exploded assembly drawing (top) and cutaway drawing (bottom) of the XAS cell for the measurement of spectra in transmission of NiF_3 and K_2NiF_6 at room temperature with the components and their respective materials: **a**) countersunk bolt DIN 7991 M3 (stainless steel), **b**) cell cap/pressure piece with a circumferential bead (3D-printed ABS), **c**) window (FEP, 100 µm), **d**) sample (NiF_3 or K_2NiF_6 powder), **e**) cell body/counter piece with a groove as a socket for **b** (aluminum). **f**) M4 thread. The samples were handled in a glovebox under argon atmosphere and portioned by the use of a self-made PTFE spatula. The cell was assembled within the glovebox.

5 Computational details

All the calculations were done within the framework of periodic DFT as implemented in the *Vienna ab initio simulation package* (VASP), version 5.4.4.[14] The plane wave basis set with the projector augmented potentials (PAW),[15] as supplied by VASP, was used, including the plane waves of up to the kinetic energy of 700 eV. Ni-3d and -4s, F-2s and -2p as well as, when applicable, K-3s, -3p and -4s electrons were treated explicitly, with others kept in core. The exchange-correlation functional was used by Perdew-Burke-Ernzerhof (PBE)[16] approach, which was previously shown to give results close to the experimental values for nickel fluorides.[10,17] To account for the strongly correlated d-electrons of nickel, a Hubbard correction in the framework of the Dudarev approach[18] was added on top of the PBE. The values of the effective Hubbard *U* parameters 5.3 eV and 6.7 eV, for Ni^{2+} and Ni^{3+}, respectively, were taken from the literature.[19] To the best of our knowledge, the *U* value for Ni^{4+} has not been previously reported in the literature, and we determined it to be 7.7 eV by benchmarking different *U* values for the structure of K_2NiF_6 (vide infra). For all structures a gamma-centered, automatically generated Monkhorst-Pack k-point grid was used to sample the first Brillouin zone – 2x4x2 for mixed-valent NiF_3, 4x4x4 for NiF_4, and 6x6x6 for K_2NiF_6. Gaussian smearing with the smearing-width of 0.1 eV was used for all the investigated structures. Convergence tests for the plane wave cut-off energy, k-grid density and the smearing-width were carried out and the values stated above all gave the total energy converged to an accuracy of 1 meV or below.

Blocked Davidson algorithm with the convergence criterion of 10^{-6} eV was employed for the electronic minimization. For ionic relaxation, the conjugate gradient algorithm was used with the convergence criterion said to be reached, when the forces on the atoms were smaller than 0.01 eV/Å.

5.1 Benchmarking the Davidson *U* value for Ni^{4+}

To the best of our knowledge, the Davidson *U* value for Ni^{4+} has not been reported in the literature. Since not many binary nickel(IV) compounds are known,[20] and even to a lesser extend well characterized,[2,21] K_2NiF_6, which is well known and its crystal structure well characterized,[4] was chosen as the benchmark for the determination of the Hubbard *U* parameter for Ni^{4+}. The *U* values were varied from 6.7 eV up to 14.2 eV with increments of 0.5 eV. It was found that by increasing the *U* value both the lattice parameters and the Ni−F bond distance changed significantly. Since the *U* value mostly determines the local environment of the atoms, it was decided to fit the *U* value to the experimental Ni−F bond distance. The best fit was found for a *U* value of 7.7 eV with a calculated Ni−F bond distance of 1.777 Å close to the experimental value of 1.776 Å[4].

5.2 Calculating the structure of mixed-valent NiF_3 ($Ni^{II}[Ni^{IV}F_6]$)

Recently, our group published a calculated solid state structure of $R3c$ symmetry, necessitating all Ni−F distances to be identical and the nickel centers to be in the equal formal oxidation state of +III.[17] In contrast, the experimentally determined solid state structure of NiF_3 contains two different Ni−F bond distances and was described as mixed-valent $Ni^{II}[Ni^{IV}F_6]$.[5] Since the experience with other nickel fluorides show that the most stable magnetic ordering is antiferromagnetic,[10,17,22] it can be assumed that such ordering would be the most stable magnetic phase in the mixed-valent NiF_3. The unit cell of NiF_3 contains 6 nickel atoms, wherein three of each should have the same formal oxidation state. This could only produce the ferrimagnetic coupling. In our investigation, we thus created two possible supercells by extending the unit cell by one in either a or b direction with 12 nickel atoms. For each of them, we investigated the 30 most likely arrangements of magnetic moments. Since we could not

predict the oxidation state of the individual nickel atoms and consequently could not assign the correct Hubbard *U* values, we used the *U* value of Ni^{3+} as a starting point. After finding the most stable magnetic phase and assigning the oxidation states to the specific nickel atoms, we then re-calculated it with the appropriate *U* values for Ni^{2+} and Ni^{4+}.

5.3 Calculation of the solid state structure of NiF_4

Despite the synthesis of NiF_4, its solid state structure is not known.[2] In order to elucidate the structure computationally, we investigated the known solid state structures of PdF_4, ZrF_4, SnF_4, RuF_4, OsF_4 and CrF_4. The results show that the most stable and therefore most probable solid state structure of NiF_4 is of the same type as RuF_4. Here we only present the Ni−F distances of the most stable NiF_4 polymorph in the space group $P2_1/c$, relative to NiF_2. A detailed investigation on the solid state structure of NiF_4 is going to be published in the near future.

5.4 Calculation of the NiF_2-normalized energies

In order to compare the thermodynamic stability of different nickel fluorides, we have calculated their energies per formula unit and then subtracted the amount of fluorine in such a way that all the energies correspond to the composition of NiF_2, i.e. NiF_3−½F_2, (Ni_2F_5−½F_2)/2, NiF_4−F_2. Since the Hubbard *U* value has a significant influence on the energy, and the energies calculated with different *U* values are not comparable, we have used the average *U* value of 6.6 eV for Ni^{2+}, Ni^{3+} and Ni^{4+} to calculate the energies. With these obtained NiF_2-normalized energies, the thermodynamic stability of different nickel fluorides can be compared.

6 References

[1] A. Guilherme Buzanich, M. Radtke, K. V. Yusenko, T. M. Stawski, A. Kulow, C. T. Cakir, B. Röder, C. Naese, R. Britzke, M. Sintschuk, F. Emmerling, *J. Chem. Phys.* **2023**, *158*, 244202.
[2] B. Žemva, K. Lutar, L. Chacón, M. Fele-Beuermann, J. Allman, C. Shen, N. Bartlett, *J. Am. Chem. Soc.* **1995**, *117*, 10025.
[3] B. Ravel, M. Newville, *J. Synchrotron Rad.* **2005**, *12*, 537.
[4] J. C. Taylor, P. W. Wilson, *J. Inorg. Nucl. Chem.* **1974**, *36*, 1561.
[5] C. Shen, L. C. Chacón, N. Rosov, S. H. Elder, J. C. Allman, N. Bartlett, *C. R. Acad. Sci., Paris, Ser. II C* **1999**, *2*, 557.
[6] L. Tröger, D. Arvanitis, K. Baberschke, H. Michaelis, U. Grimm, E. Zschech, *Phys. Rev. B* **1992**, *46*, 3283.
[7] OriginLab Corporation, *Origin(Pro), Version 2020*, Northhampton, MA, USA.
[8] W. Jauch, A. Palmer, A. J. Schultz, *Acta Cryst. B* **1993**, *49*, 984.
[9] E. R. Jette, F. Foote, *J. Chem. Phys.* **1935**, *3*, 605.
[10] S. Mattsson, G. Senges, S. Riedel, B. Paulus, *Chem. Eur. J.* **2020**, *26*, 10781.
[11] N. Watanabe, *J. Fluorine Chem.* **1983**, *22*, 205.
[12] a) J. H. Simons, J. W. Bouknight, *J. Am. Chem. Soc.* **1932**, *54*, 129; b) J. J. Jasper, *J. Phys. Chem. Ref. Data* **1972**, *1*, 841.
[13] Z. Mazej, E. A. Goreshnik, *J. Fluorine Chem.* **2017**, *196*, 3.
[14] a) G. Kresse, J. Hafner, *Phys. Rev. B* **1993**, *47*, 558; b) G. Kresse, J. Furthmüller, *Comput. Mater. Sci.* **1996**, *6*, 15; c) G. Kresse, J. Hafner, *Phys. Rev. B* **1994**, *49*, 14251.

[15] a) G. Kresse, D. Joubert, *Phys. Rev. B* **1999**, *59*, 1758; b) P. E. Blöchl, *Phys. Rev. B* **1994**, *50*, 17953.
[16] J. P. Perdew, K. Burke, M. Ernzerhof, *Phys. Rev. Lett.* **1996**, *77*, 3865.
[17] S. Mattsson, B. Paulus, *J. Comput. Chem.* **2019**, *40*, 1190.
[18] S. L. Dudarev, G. A. Botton, S. Y. Savrasov, C. J. Humphreys, A. P. Sutton, *Phys. Rev. B* **1998**, *57*, 1505.
[19] F. Zhou, M. Cococcioni, C. A. Marianetti, D. Morgan, G. Ceder, *Phys. Rev. B* **2004**, *70*, 235121.
[20] A. F. Holleman, N. Wiberg, *Lehrbuch der anorganischen Chemie*, de Gruyter, Berlin, **2007**.
[21] J. M. Tarascon, G. Vaughan, Y. Chabre, L. Seguin, M. Anne, P. Strobel, G. Amatucci, *J. Solid State Chem.* **1999**, *147*, 410.
[22] T. Lindič, S. Sinha, S. Mattsson, B. Paulus, *Z. Naturforsch. B* **2022**, *77*, 469.
